# Experience modifies the synaptome architecture in the developing and adult mouse brain

**DOI:** 10.1038/s41467-026-78702-7

**Published:** 2026-11-11

**Authors:** Hanan Woods, Ülkü Günar, Sarah Catherine Gillard, Beverley Notman, Kunhao Yuan, Digin Dominic, Emily Robson, Gabor Varga, Noboru H. Komiyama, Zhen Qiu, Frank Sengpiel, Seth G. N. Grant

**Affiliations:** 1 Genes to Cognition Programme, Institute for Neuroscience and Cardiovascular Research, University of Edinburgh, Edinburgh, UK; 2 Simons Initiative for the Developing Brain (SIDB), Institute for Neuroscience and Cardiovascular Research, University of Edinburgh, Edinburgh, UK; 3 The Patrick Wild Centre for Research into Autism, Fragile X Syndrome & Intellectual Disabilities, Institute for Neuroscience and Cardiovascular Research, University of Edinburgh, Edinburgh, UK; 4 Muir Maxwell Epilepsy Centre, Institute for Neuroscience and Cardiovascular Research, University of Edinburgh, Edinburgh, UK; 5 Department of Biomedical Engineering, University of Strathclyde, Glasgow, UK; 6 School of Biosciences, Cardiff University, Cardiff, UK; 7 Euan MacDonald Centre, University of Edinburgh, Edinburgh, UK

**Keywords:** Synaptic plasticity, Development of the nervous system

## Abstract

Synaptome architecture describes the spatiotemporal distribution of highly diverse excitatory synapses throughout the brain. Whether and how this architecture is impacted by experience and sensory input is unknown. We found that environmental enrichment and monocular visual deprivation drive large-scale reconfiguration of excitatory synapse populations in more than one hundred regions of the mouse brain. Different experiences modify distinct subsets of synapse types and subtypes, with patterns aligned with protein turnover rates and connectome architecture. Similarity in experience-dependent reconfiguration during development and adulthood indicates shared mechanisms of synaptome adaptation across the lifespan. Our findings support a population-selection model in which experiences drive adaptation by selectively modifying synapse types and subtypes distributed across the brain. Experience-driven reconfiguration of synaptome architecture shares features with the response to aging, sleep deprivation, brain injury and genetic mutations, supporting a unifying principle of brain adaptation.

## Abstract (web summary)

The effect of experiences on brain-wide synapse populations is unknown. Here, the authors show that environment and visual input drive changes in excitatory synapse types and subtypes across the mouse brain during development and adulthood.

## Introduction

The vast majority of brain synapses are excitatory, and their proteomes are highly complex^1–12^ and extraordinarily diverse^13–16^. Individual synapses differ in their protein composition^13,16^, rate of protein turnover^17^ and their nanoarchitecture^18–20^. Systematic, data-driven analysis of individual synapse proteins has led to the concept of the synaptome—the complete population of diverse synapse types in the brain—and its spatial and temporal organization, known as the synaptome architecture^16^. Single-synapse resolution mapping of excitatory synapse types and subtypes across the mouse brain has revealed that the synaptome architecture changes continuously across the lifespan^21,22^ and in response to genetic mutations^16,22^ and that sleep is essential for its maintenance^23^. Together, these findings indicate that the synaptome and synaptome architecture are genetically programmed and flexible.

Whether experience alters the synaptome architecture remains an open question. To investigate environmental influences, we used the environmental enrichment (EE) paradigm^24,25^, comparing mice in complex environments with those housed in standard cages (SC). We explored the effects of raising mice from birth until maturity in these two environments (referred to as Developmental EE) as well as the effects of shifting adult mice raised in an SC to an EE environment (referred to as Adult EE) (Supplementary Fig. S1A). To assess the impact of specific sensory input during development, we also examined mice subjected to one week of monocular deprivation (MD) during the postnatal critical period^26,27^ (Supplementary Fig. S1A).

Synaptome mapping technology enables systematic single-synapse analysis of protein composition, turnover and morphology across the mouse brain by imaging endogenously tagged synaptic proteins in tissue sections^16,17,21–23^ (Supplementary Fig. S1B). Fluorescent^16^ or self-labelling^17^ tags report protein abundance and turnover, allowing classification of excitatory synapses into types and subtypes, revealing extensive molecular diversity across brain regions. In this study, we applied an established synaptome mapping pipeline to classify excitatory synapses based on the expression of the postsynaptic scaffold proteins PSD95 and SAP102 tagged with eGFP and mKO2, respectively (Supplementary Fig. S1B). These abundant MAGUK proteins interact with NMDA and AMPA receptors and play central roles in synaptic transmission, plasticity, and cognitive function^28–32^. Their combinatorial expression defines three excitatory synapse types: Type 1 (PSD95 only), Type 2 (SAP102 only), and Type 3 (dual labelled). Within this framework, synapses are further resolved into 37 subtypes based on quantitative features including protein intensity, size, and morphology^16^. Of these, 30 PSD95-containing subtypes have been stratified according to PSD95 turnover kinetics into short (SPL), medium (MPL), and long (LPL) protein lifetime classes^17^. This data-driven classification was based on systematic studies of excitatory synapses from over 100 brain regions and provides a basis for asking how populations of molecularly diverse synapses are distributed and regulated.

We show that distinct experiences during development induce selective and spatially distributed alterations in specific excitatory synapse types and subtypes. We also compare the effect of experience in mature animals with developing animals and examine the roles of protein turnover and the connectome architecture. These findings demonstrate that experience and sensory input result in modifications to defined synapse populations, revealing a crucial role for synapse diversity in core brain function.

### Experience shapes synaptome architecture

**Fig. 1:**
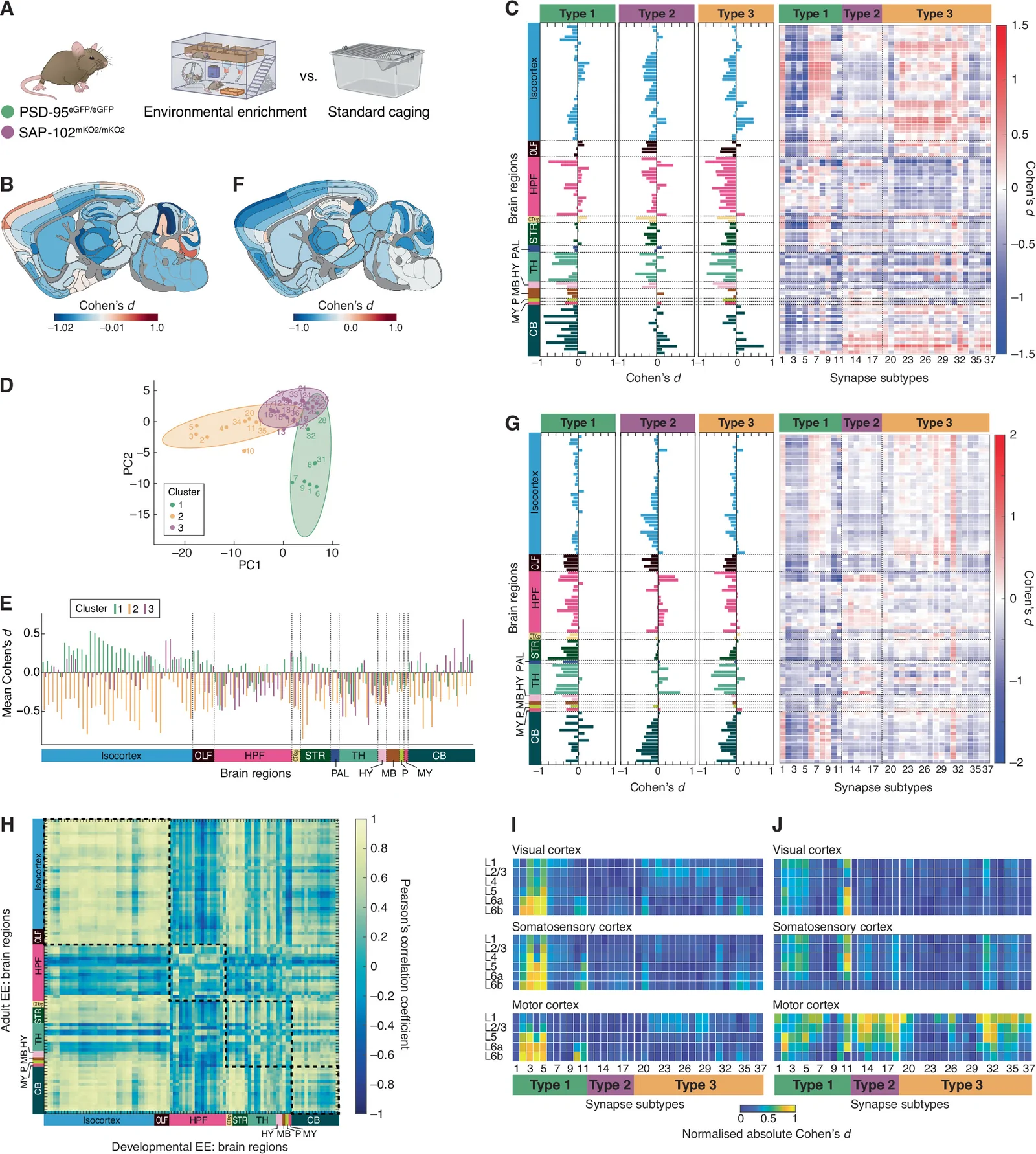
Developmental and adult environmental enrichment remodel brain synaptome architecture. **A** Experimental groups. *Psd95*^*eGFP/eGFP*^ and *Sap102*^*mKO2/mKO2*^ mice were reared under environmental enrichment (EE; *n* = 23, 12 females) or standard cage (SC; *n* = 29, 15 females) conditions for 3 months from birth. In a separate experiment, adult SC-housed mice were transferred to EE (*n* = 20, all female) or remained in SC (*n* = 20, all female) for 1 month. Parasagittal maps of Cohen’s *d* for total synapse density in developmental (**B**) and adult (**F**) cohorts. Positive values indicate higher density in EE. Statistical testing and FDR correction are reported in the Source Data file; region labels are in Supplementary Fig. S32. Regional Cohen’s *d* values for three synapse types (bars) and 37 subtypes (heatmaps), in developmental (**C**) and adult (**G**) paradigms. Effect sizes > 0 reflect enrichment-related increases. Expanded heatmaps with region labels and significance markers are in Supplementary Figs. S2, S10. **D** Robust PCA biplot of subtype effect-size profiles across regions. Colours indicate clusters defined by hierarchical clustering of PCA scores; proximity denotes similar anatomical effect-size profiles. **E** Mean regional Cohen’s *d* stratified by subtype cluster. Values were averaged across subtypes within each cluster. Expanded panel in Supplementary Fig. S3. **H** Pearson correlation matrix comparing developmental and adult EE subtype-by-region effect-size matrices. Normalized absolute Cohen’s *d* for subtype-layer combinations in visual, somatosensory and motor cortex in developmental (**I**) and adult (**J**) EE. Values were divided by the maximum effect size across layers and subtypes within each cortical area. Brain region abbreviations are listed in Supplementary Information, ‘Brain region list’.

As a first step toward establishing whether synaptome architecture is shaped by experience we reared mice expressing PSD95-eGFP and SAP102-mKO2 from birth to 3 months in enriched (EE; *n* = 23, 12 female/ 11 male) or standard (SC; *n* = 29, 15 female/ 14 male) housing (Fig. 1A, Supplementary Fig. S1A). This developmental exposure window was chosen to span the major phase of synaptome maturation previously reported to extend to 3 months of age^21^. Using our synaptome mapping pipeline, we analysed an average of 23.9 million excitatory synaptic puncta across 100 brain regions from each mouse and mapped the location of three types and 37 subtypes of excitatory synapse. The systematic nature of our analysis contrasts with prior studies that have typically sampled fewer than 5000 synapses per mouse in a single brain region in very few animals. Because subtype labels were assigned using a common predefined classification framework applied consistently across all samples, the resulting subtype densities could be compared across animals, brain regions, and experimental conditions. We used Bayesian group comparisons with FDR correction to identify statistically supported differences in total synapse density, while Cohen’s *d* was used throughout the figures to show the magnitude and direction of effects.

Surprisingly, unlike some earlier studies^33–39^, we found EE did not lead to a greater density of excitatory synapses across the whole brain, major brain regions, or subregions (Bayesian analysis with FDR correction; Fig. 1B). Instead, a modest trend toward lower synapse density emerged across many regions, with a median decrease of 3% observed across subregions. These findings suggest that this EE paradigm does not broadly promote global synaptogenesis, but instead may drive more subtle, selective remodelling within specific excitatory synapse populations.

We therefore examined whether the density of each synapse type and subtype in each brain region differed between mice raised in SC and EE environments. The regional differences (Cohen’s *d*) are shown as bar graphs for synapse types and as heatmaps for subtypes (Fig. 1C, Supplementary Fig. S2). This revealed a striking differential pattern in the synaptome across the whole brain. EE resulted in a reduction in the density of Type 1 synapses in the molecular layer of the cerebellum, thalamus, and dentate gyrus (Fig. 1C, Supplementary Fig. S2). A subset of Type 1 subtypes (2–5, 10, 11) were consistently reduced in EE mice across diverse structures, including the deep cortical layers, cerebellar layers, thalamus, hypothalamus, striatum, pallidum, olfactory regions, and cortical subplate (Fig. 1C, Supplementary Fig. S2). By contrast, the remaining Type 1 subtypes (1, 6–9) were increased in several regions, most notably in the cortex. Moreover, the strongest synaptic changes in the cortex were found in the deeper layers for subtypes 2, 3, 4 and 5. Differences in Type 3 synapse subtype densities showed similar trends across cortical regions, including differential effects in deep and superficial layers, and between the hippocampus, thalamus, and hypothalamus, whereas Type 2 synapses were largely unaffected (Fig. 1C, Supplementary Fig. S2).

To identify coordinated patterns in groups of subtypes that might not be evident from individual subtype-region comparisons, we applied robust Principal Component Analysis (PCA) to the subtype-by-region Cohen’s *d* matrix. In this analysis, each subtype is represented by its effect-size profile across brain regions, so subtypes that occupy similar positions in PCA space show similar anatomical patterns of change (Fig. 1D). The first three components accounted for 43.7%, 19.3%, and 12.0% of the variance, respectively, with the stability of PC1 confirmed by bootstrap resampling (95% CI: 37.4–67.6%) and permutation testing (*p* < 0.001). Hierarchical clustering of PCA scores yielded three stable clusters (Jaccard indices: 0.81, 0.68 and 0.79), which showed robust separation along the first two principal components (Kruskal–Wallis, PC1: χ² = 24.9, df = 2, *p* = 3.9 × 10⁻⁶; PC2: χ² = 23.4, df = 2, *p* = 8.1 × 10⁻⁶). These clusters therefore describe groups of synapse subtypes with similar spatial patterns of enrichment-related change, rather than new molecular subtype categories. Notably, Cluster 2 contained most subtypes shown above to decrease with EE (subtypes 2–5, 10, 11, 20, 34, 35; Fig. 1D) and was maximally distinct from Cluster 1 (subtypes 1, 6–9, 28, 31, 32) along PC1. We then projected these PCA-derived subtype clusters back onto brain anatomy by averaging Cohen’s *d* values within each cluster for each region. EE-dependent divergence in these clusters was most evident in the isocortex, striatum, olfactory areas, putamen, and cortical subplate (Fig. 1E, Supplementary Fig. S3). Cluster 3 subtypes largely correspond to those that showed little change in response to EE – all of the Type 2 subtypes (12–18) and the remaining Type 3 subtypes (19, 21-27, 29, 30, 33, 36, 37). Evaluating sex as a factor revealed region- and subtype-specific trends in synaptome architecture with moderate effect sizes (Supplementary Figs. S17–S22); however, no sex-dependent differences survived strict false discovery rate (FDR) correction (Supplementary Figs. S4–S9). Together, these findings support a model in which sensory experiences during development drive selective, type- and subtype-specific shifts in synapse populations across brain regions.

### EE remodels the synaptome architecture of the adult brain

It is generally thought that the developing brain is more plastic than the mature brain^40,41^. To explore this issue, we asked whether exposing mature mice (6–12 months of age) for one month to the same EE used in the aforementioned experiments would induce a change in synaptome architecture. This age window was selected because prior lifespan synaptome mapping showed that synaptome architecture is relatively stable between 3 and 12 months of age^21^. Strikingly, despite this more stable baseline period, adult EE induced the same broad pattern of synaptome reprogramming observed in the developmental EE experiment. Total synapse density was modestly reduced, and subtype-specific decreases were detected in subtypes 2–5, 10, 11, 20, 34, and 35, closely matching the EE-sensitive cluster identified in the developmental cohort (Fig. 1F, G, Supplementary Fig. S10). To establish whether developmental and adult EE affected similar subtype-region combinations, we correlated the two Cohen’s *d* matrices across corresponding anatomical domains. The similarity matrix shows highest similarity in the isocortex, with hippocampal, subcortical and cerebellar regions showing differential responses between developmental and adult brain (Fig. 1H). This regional divergence of adult and developmental EE effects was also apparent at the level of synapse types, including a lack of Type 3 changes in the hippocampus and an overall decrease of SAP102+ synapses in the cerebellum with adult EE (Fig. 1C, G). In the isocortex the main effects were in the deep layers with developmental EE, whereas they were in the superficial layers with adult EE (Fig. 1I, J). Moreover, Type 2 and 3 subtypes in the motor cortex and orbital cortex were differentially affected between developmental and adult EE paradigms (Fig. 1C, G, I, J).

### MD drives global synaptome changes

**Fig. 2:**
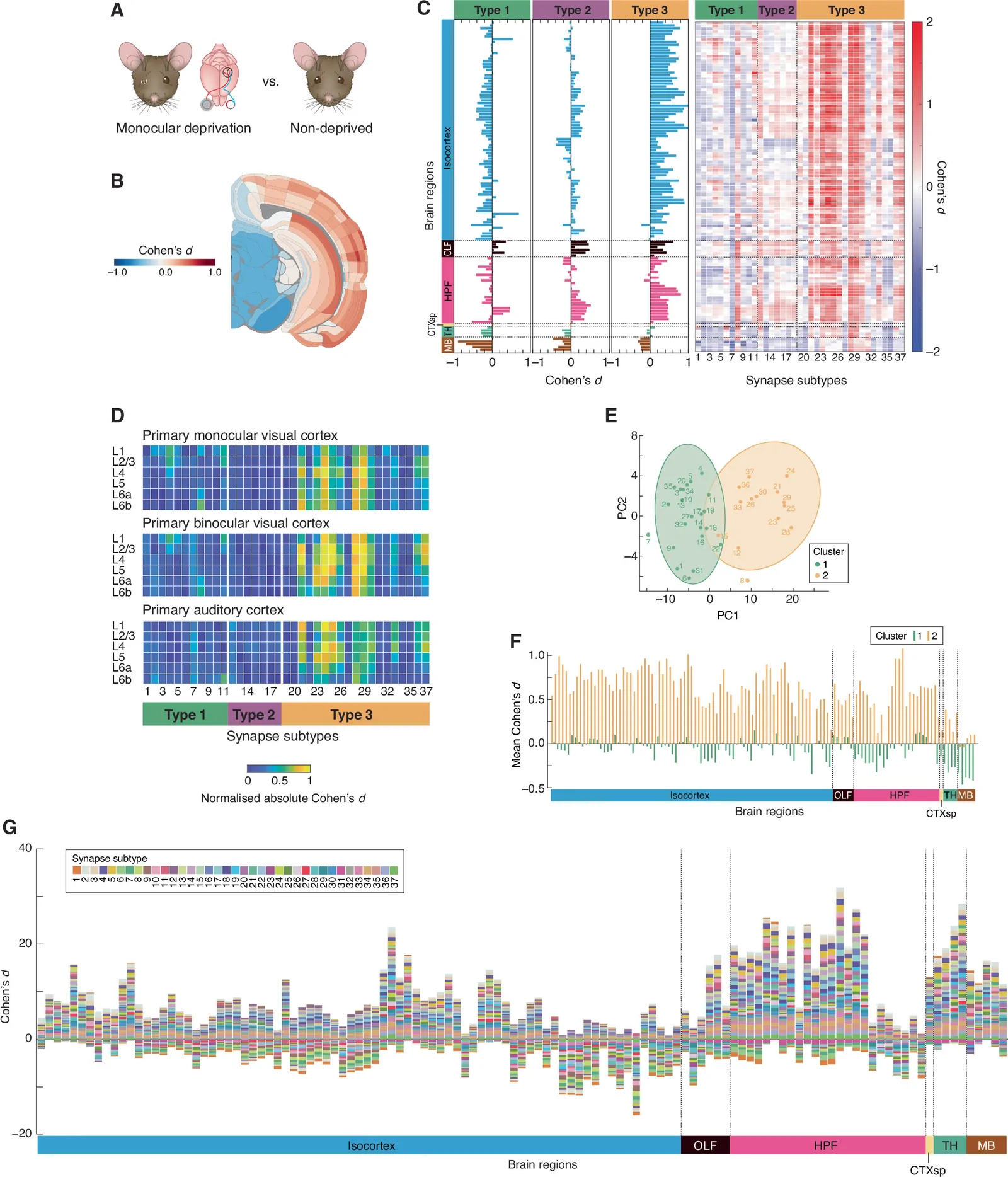
Monocular deprivation remodels synaptome architecture in visual and non-visual cortical regions. **A** Experimental design. Mice homozygous for *Psd95*^*eGFP*^ and *Sap102*^*mKO2*^ underwent monocular deprivation (MD) via right eyelid suture from postnatal day (P) 25/26 to P31 (*n* = 13, 7 female) and were compared with non-deprived controls (CTRL; *n* = 14, 7 female). Deprivation primarily targeted the contralateral (left) monocular primary visual cortex and partially targeted binocular visual cortex bilaterally. **B** Coronal map of regional Cohen’s *d* for total synapse density, MD versus CTRL. Positive values indicate higher density in MD. Region labels are in Supplementary Fig. S31. **C** Regional Cohen’s *d* for three synapse types (bars) and 37 subtypes (heatmaps), MD versus CTRL. Positive values indicate MD-associated increases. Expanded panels with region labels and significance markers are in Supplementary Fig. S11. **D** Normalized absolute Cohen’s *d* for subtype-layer combinations in monocular and binocular primary visual cortex and primary auditory cortex. Values were divided by the maximum effect size across layers and subtypes within each region. **E** Robust PCA biplot of subtype effect-size profiles across regions. Colours indicate clusters defined by hierarchical clustering of PCA scores; proximity denotes similar anatomical response profiles. **F** Mean regional Cohen’s *d* stratified by subtype cluster. Expanded panel in Supplementary Fig. S13. **G** Cohen’s *d* for 37 synapse subtypes in the contralateral hemisphere after normalization of contralateral density to summed bilateral density. Positive values indicate higher relative density in MD. Expanded panel in Supplementary Fig. S16. Brain region abbreviations are listed in Supplementary Information, ‘Brain region list’.

The brainwide changes in synaptome architecture induced by EE might have arisen because of input to multiple sensory pathways. We therefore explored the impact of a limited sensory deprivation, namely MD, in mice from postnatal day 25/26 to 31 (treated *n* = 13, 7 females/6 males; control *n* = 14, 7 females/7 males) (Fig. 2A, Supplementary Fig. S1). At the end of the deprivation period, coronal sections of both hemispheres, which included the primary and secondary visual regions among a total of 119 regions, were analysed. In each section we mapped an average of 32.5 million excitatory synaptic puncta, which contrasts with earlier studies analysing fewer than 5000 synapses in only visual regions^42–44^.

Although one might anticipate that MD would produce synaptome changes primarily in the visual system, we observed distributed impacts broadly similar to those in response to EE. MD did not significantly alter total excitatory synapse density across the whole brain or within major brain regions (Bayesian analysis with FDR correction; Fig. 2B) but there were striking changes in the densities of particular synapse types and subtypes on a brainwide scale (Fig. 2C, Supplementary Figs. S11, S12). Type 3 synapses were consistently elevated across nearly all isocortical and hippocampal subregions in the deprived hemisphere (Fig. 2C, Supplementary Fig. S11). Type 1 and 2 synapse populations exhibited smaller, region-specific changes. These effects were not confined to the visual cortex or even the deprived contralateral hemisphere. Type 3 synapse increases were mirrored in the non-deprived, ipsilateral hemisphere and extended into multiple sensory cortices, suggesting a distributed cortical response to the MD paradigm (Supplementary Fig. S12). Synapse subtype analysis further revealed that specific Type 3 subtypes (21, 23–26, 28–30, 33, 36, 37) were elevated across both hemispheres in MD mice, including in auditory, somatosensory, and hippocampal regions (Fig. 2C, D, Supplementary Figs. S11, S12).

We next asked whether MD-affected subtypes formed coordinated groups by applying the same robust PCA approach to the subtype-by-region Cohen’s *d* matrix. As in the EE analysis, each subtype was represented by its anatomical effect-size profile, allowing subtypes with similar regional responses to MD to cluster together. PC1 explained 79.6% of the variance (95% CI: 60.7–86.3%; *p* < 0.001), indicating a dominant and structured response to MD. Hierarchical clustering in PCA space identified two clusters (Jaccard indices: 0.524, 0.635), differing significantly in PC1 scores (Kruskal–Wallis, PC1: χ² = 25.4, df = 1, *p* = 4.6 × 10^−7^; PC2: χ² = 0.09, df = 1, *p* = 0.75). These clusters summarise coordinated MD-response profiles across regions that aid the interpretation of subtype-level effect-size patterns. These subtype groupings differed from those identified for the EE paradigm. Here, Cluster 2 encompassed the MD-sensitive Type 3 subtypes (21, 23–26, 28–30, 33, 36, 37) as well as subtypes 8, 12 and 15, supporting their coordinated elevation across multiple regions (Fig. 2E). Mapping these clusters back onto anatomical regions showed where the coordinated subtype responses were strongest. Whereas MD was associated with localized increases in Type 3 subtypes, particularly within isocortical and hippocampal regions, EE produced a subtle downscaling of Type 1 and 3 subtypes (Fig. 2C, F, Supplementary Fig. S13, Fig. 1C, E). Thus, EE and MD paradigms reprogram the synaptome through selective and distributed changes in distinct synapse types and subtypes.

To assess hemisphere-specific effects of MD, we developed an asymmetry score, quantifying the proportion of synapse density in the contralateral hemisphere relative to the summed bilateral density in any given region (see methods). MD increased the relative representation of specific synapse subtypes in the contralateral hemisphere, particularly in the anteromedial and monocular visual cortex, ventral retrosplenial cortex, hippocampal formation, thalamus, and midbrain (Fig. 2G, Supplementary Figs. S14, S16). In monocular visual cortex, this shift was most pronounced for subtype 2 in layers 1 and 2/3, although subsets of Type 1, Type 2, and Type 3 subtypes also showed increased contralateral representation. We also calculated an inter-hemispheric difference score to further highlight effects specific to the deprived hemisphere, relative to the internal control hemisphere. Brain-wide changes were observed, with a trend of reduced absolute inter-hemispheric differences across several subtypes and regions in MD compared to control groups (Supplementary Figs. S15, S16). The widely reported reduced asymmetry of responses to stimulation of the two eyes in the visual cortical hemisphere contralateral to the deprived eye^45,46^, could potentially be explained by those changes in synaptome architecture. By contrast, the inter-hemispheric difference increased some subtypes in the CA1 and ventral subiculum, indicating that MD drives region-specific patterns of inter-hemispheric reorganisation (Supplementary Figs. S15, S16). As observed for EE, evaluating sex as a factor in MD revealed region- and subtype-specific trends with moderate effect sizes, though no sex-dependent differences survived false discovery rate (FDR) correction (Supplementary Figs. S17–S22; Bayesian analysis and FDR correction).

### Protein lifetime constrains synaptome plasticity

Our findings point to a key question: why do only certain synapse subtypes undergo structural change following EE or MD? We hypothesized that the capacity for change may be governed, in part, by the rate of protein turnover in synapses. In this setting, synapse subtypes built from long-lived proteins may respond differently to experience than those composed of proteins that turn over more rapidly.

**Fig. 3:**
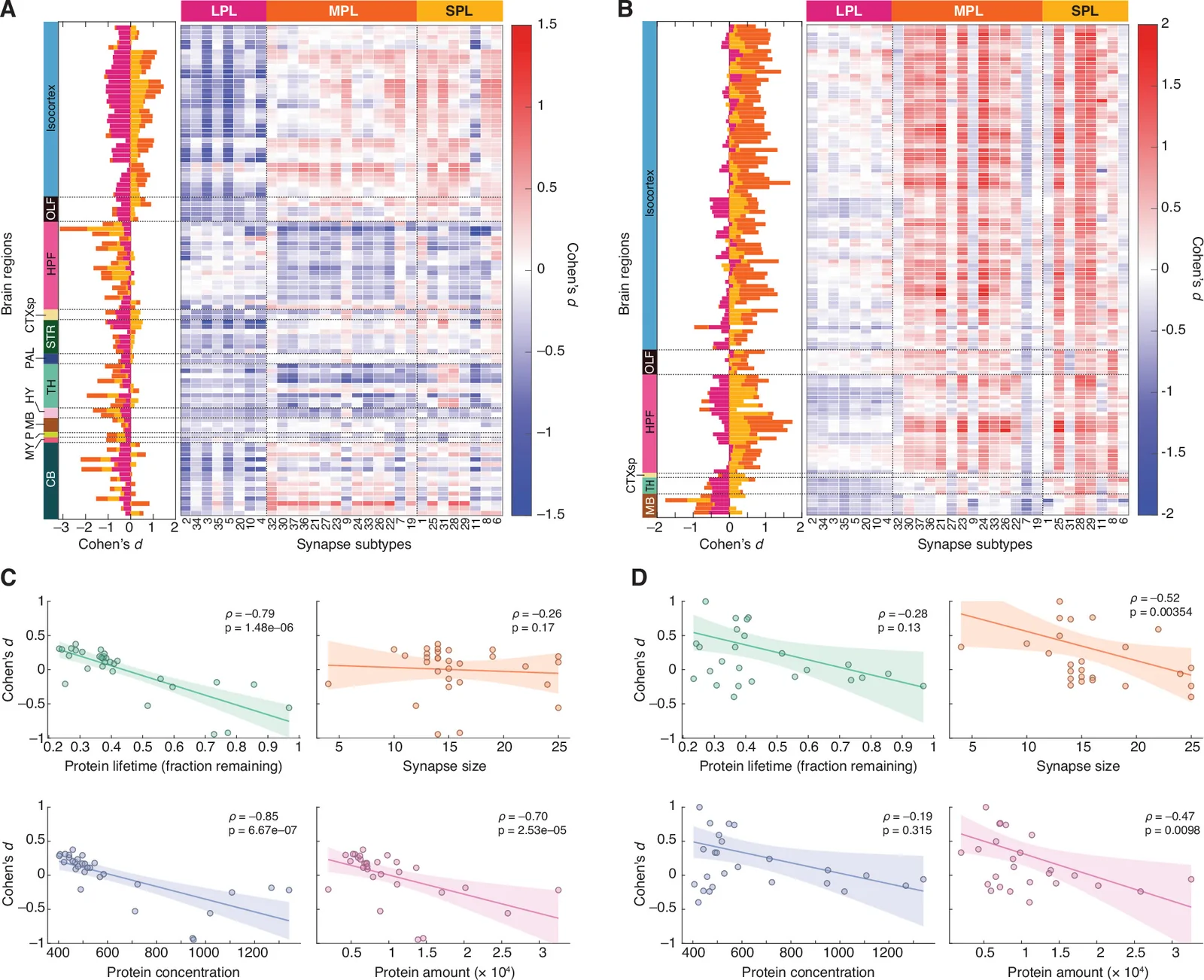
Experience-dependent plasticity selectively targets synapse subtypes according to their protein lifetime. Regional Cohen’s *d* values for 30 PSD95⁺ synapse subtypes, ordered by protein lifetime, comparing developmental environmental enrichment (EE) with standard cage (SC) controls (**A**) and monocular deprivation (MD) with non-deprived controls (**B**). Stacked bars show aggregate effect sizes for short (SPL; 8 shortest-lifetime subtypes), medium (MPL; 14 intermediate-lifetime subtypes), and long (LPL; 8 longest-lifetime subtypes) protein-lifetime groups. Subtype effect-size data are from Figs. 1C and 2C and were reordered using independent PSD95 lifetime annotations. Lifetime classes were defined from previously measured PSD95 turnover and were independent of the EE and MD datasets. Expanded heatmaps are in Supplementary Figs. S23, S24. Relationships between subtype effect size and independently measured protein turnover or structural properties across 30 PSD95⁺ subtypes in the isocortex for developmental EE (**C**) and MD paradigms (**D**). Protein lifetime is expressed as fraction remaining after 7 days; synapse size as punctum area (pixels); protein concentration as mean PSD95⁺ punctum intensity; and total protein amount as summed PSD95⁺ punctum intensity. Spearman’s ρ and associated *p*-values are shown.

To test this hypothesis, we aligned experience-sensitive effect-size data for each of the 30 PSD95-positive Type 1 and Type 3 subtypes with independent PSD95 lifetime estimates reported previously^17^. We then reordered the subtype heatmaps according to these prior lifetime annotations and grouped subtypes into short (SPL), medium (MPL), and long (LPL) protein-lifetime classes (Fig. 3A, B, Supplementary Figs. S23, S24). This ordering revealed distinct subtype-level patterns that were less apparent when subtypes were arranged by numerical label alone. In the EE datasets, the most prominent changes were concentrated among LPL subtypes, which were preferentially reduced. By contrast, MD was associated with increased density in several MPL and SPL subtypes across both hemispheres, with an additional decrease of LPL subtypes in the non-deprived hemisphere (Fig. 3A, B, Supplementary Figs. S23, S24). For MD, these patterns were broadly consistent across isocortical and hippocampal regions, whereas in EE the hippocampus showed additional suppression of SPL subtypes. In a separate study of sleep deprivation, we previously observed a different lifetime-ordered striping pattern, in which LPL subtypes were preferentially increased (Supplementary Fig. S25)^23^. Interestingly, this pattern was the opposite of that observed for EE, where LPL synapses were downregulated (Supplementary Fig. S25). Similarly, SPL and MPL synapses were upregulated with MD but were downregulated with sleep deprivation (Supplementary Fig. S25).

In addition to protein lifetime, synapse subtypes also vary in protein amount, protein concentration and synapse size^16,17^. We asked which of these parameters correlates with the effects induced by EE and MD. EE-induced changes were strongly and negatively correlated with protein lifetime (*p* = 1.48 ×10^−6^), protein concentration (*p* = 6.67 ×10^−7^), and protein amount (*p* = 2.53 ×10^−5^) but not with synapse size (*p* = 0.17) (Fig. 3C). By contrast, MD-related effect sizes showed only weak associations, with moderate negative correlations to synapse size (*p* = 3.54 ×10^−3^) and protein amount (*p* = 9.8 ×10^−3^), but no significant relationship to protein lifetime or concentration (Fig. 3D).

Together, these analyses suggest that a component of experience-associated subtype remodelling is related to subtype-level proteostatic properties. In particular, EE preferentially targeted subtypes with longer PSD95 lifetimes and higher PSD95 content for downscaling, whereas MD was more strongly associated with changes in medium-sized subtypes with more dynamic protein content. These findings support the idea that synapse proteostasis is an important correlate of experience-associated synaptome reprogramming and may influence how different synapse subtypes respond to environmental and sensory perturbation.

### Synaptome changes align with the connectome

In addition to a role of synapses with different protein turnover rates, we hypothesized that the connectivity between brain regions might contribute to the spatial reorganisation of synaptome architecture in response to experience. To explore this, we integrated our synaptome data with connectome data from the Allen Mouse Brain Connectivity Atlas^47,48^. From tracer injections across 129 source regions, we extracted projection densities to 73 parasagittal or 91 coronal target sites, aligning these to our synaptome architecture regions (Supplementary Fig. S26). We then computed Spearman’s correlations between projection density and either control subtype density (ρᴰ) or absolute effect size following enrichment or deprivation (ρᶜᵈ), applying FDR correction across all comparisons. Thus, ρᴰ asks where baseline subtype distributions align with known projection architecture, whereas ρᶜᵈ asks where experience-induced subtype changes align with the same connectivity framework. We treated these analyses as spatial association analyses rather than evidence of direct anatomical connectivity between individual synapses and tracer-labelled projections.

Hierarchical clustering of ρᴰ and ρᶜᵈ revealed coherent subtype groupings with shared projection profiles in both control mice and mice exposed to EE or MD. When baseline subtype densities in control mice were correlated with baseline projection architecture (ρᴰ), LPL synapse subtypes showed their strongest positive correlations with sensory pathways, including visual, auditory, somatosensory, and motor thalamo-cortical circuit regions, across all ages tested (1 to 12 months) (Supplementary Figs. S27–S29).

**Fig. 4:**
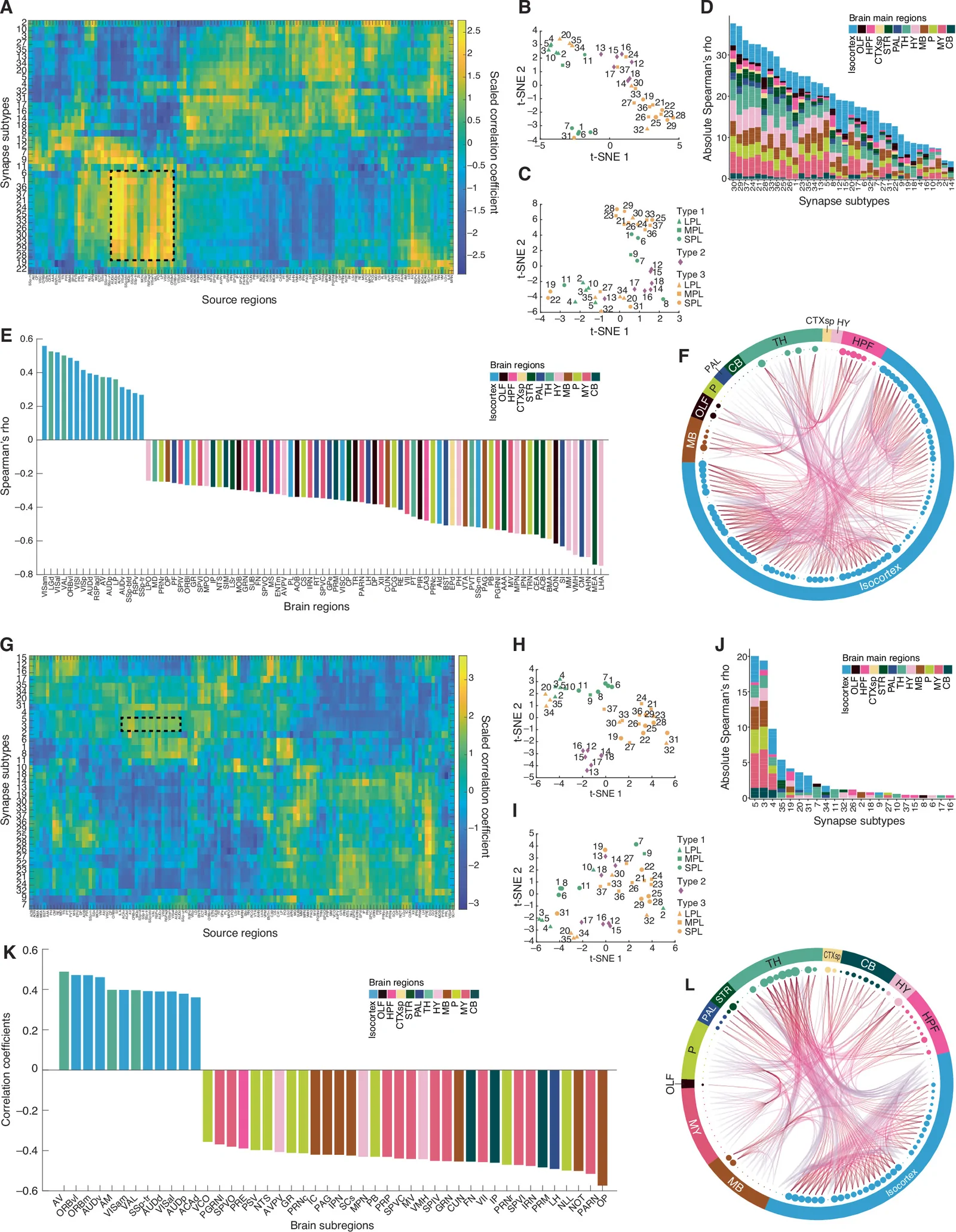
Synapse subtype remodelling under MD and EE aligns with mesoscale sensory connectivity. **A**, **G** Clustered heatmaps of scaled Spearman correlation coefficients (ρ^**cd**^) between synapse subtype effect-size profiles (Cohen’s *d*) and projection densities from 129 source regions to 91 target regions, following monocular deprivation (MD; **A**) or 73 target regions following developmental environmental enrichment (EE; **G**). Rows show 30 PSD95⁺ subtypes; columns show source regions. Both axes were hierarchically clustered using Pearson correlation distance. Boxes highlight positive correlations involving MD- or EE-responsive subtype clusters and thalamocortical sensory regions. ρᶜᵈ measures spatial alignment between subtype effect-size maps and mesoscale projection maps, not direct synaptic connectivity. Expanded heatmaps are in Supplementary Figs. S27, S28. t-SNE projections of subtype density correlation profiles (ρ^D^) in 1-month (**B**) and 3-month (**H**) control mice. Marker colour denotes synapse type and shape denotes protein-lifetime class. UMAP validation is in Supplementary Fig. S30. t-SNE projections of subtype effect-size correlation profiles (ρ^cd^) under MD (**C**) and EE (**I**); colours and shapes as in B,H. UMAP plots are in Supplementary Fig. S30. **D**, **J** Stacked bars of significant absolute ρ^cd^ values (*p* < 0.019 for MD; *p* < 0.002 for EE; FDR-corrected) by subtype, coloured by source-region domain. **E**, **K** Significant source-region ρ^cd^ values for representative subtypes: MD-responsive (subtype 29) and EE-responsive (subtype 5). **F**, **L** Anatomically structured edge bundling graphs illustrating mesoscale projection networks linked to representative MD-responsive (subtype 29) and EE-responsive (subtype 5) subtypes. Nodes represent brain subregions coloured by anatomical domain; edges reflect 500 strongest projections from significantly correlated source regions; node size indicates cumulative ρ^cd^ magnitude. These graphs summarize correlated projection pathways and do not reconstruct individual synaptic connections. Brain region abbreviations are listed in Supplementary Information, ‘Brain region list’.

We then asked a separate question: which subtype changes induced by MD were most strongly aligned with visual pathway connectivity? In this analysis, projection densities were correlated with MD-induced subtype effect sizes (ρᶜᵈ), rather than with baseline subtype densities. Here, the strongest positive correlations with sensory pathways were observed for changes in Type 3 MPL and SPL subtypes, including correlations involving the lateral geniculate nucleus and visual cortices (boxed area in Fig. 4A; Supplementary Fig. S27). Conversely, these same pathways negatively correlated with many LPL and Type 2 subtypes. We used t-SNE and UMAP as complementary visual summaries of the high-dimensional correlation profiles. The overlap between these embeddings and the PCA-derived groupings supported the presence of recurring subtype groupings across analytical approaches, consistent with experience-associated changes targeting pre-existing subsets of excitatory synapses embedded within sensory circuitry (Figs. 2D, 4B, C, Supplementary Fig. S30). We next examined subtype 29 as an example of an MD-responsive SPL subtype with strong visual pathway-associated correlations. We identified significant positive correlations with brainwide projections (*p* < 0.019, FDR-corrected; Fig. 4D), with the strongest relationships from the visual pathway, including the lateral geniculate nucleus and anteromedial visual areas (Fig. 4E). A hierarchical bundling graph, limited to the 500 strongest projection pairs from significantly correlated source regions, highlighted the dense intra-cortical network underscoring these correlations (Fig. 4F). This subtype-specific example supports the view that MD-induced synaptome changes are not distributed uniformly across the brain but correlated with defined patterns of sensory-system connectivity.

We next applied the same connectome–synaptome correlation framework to EE-induced synaptome changes to determine whether experience-dependent relationships with brainwide connectivity differed from those observed following MD. Specifically, we correlated projection densities with EE-induced subtype effect sizes (ρᶜᵈ), analogous to the analysis performed for MD (Fig. 4A, G). EE revealed a distinct correlation profile from MD (Fig. 4G, Supplementary Figs. S27–S29). Among the few subtypes significantly correlated with projection densities, subtype 5 aligned with sensory thalamo-cortical projections, including those involved in spatial navigation, attention, and motor planning (Fig. 4G, J, K and Supplementary Fig. S28). In both ρᴰ and ρᶜᵈ t-SNE and UMAP plots, LPL synapses were spatially grouped toward one end of the axis, indicating shared connectivity-correlation profiles for subtype control densities and EE effects (Fig. 4H, I and Supplementary Fig. S30). A hierarchical bundling graph highlights the brainwide connections underscoring these correlation profiles (Fig. 4L). Together, these findings indicate that there is an intrinsic spatial relationship between the connectome and the synaptome architecture, which positions sensory pathways to drive changes in synapse populations in response to experience.

## Discussion

Our study demonstrates that environmental experience and sensory input drive widespread, selective remodelling of excitatory synapses across the brain in developing and mature mice. Using single-synapse resolution synaptome mapping of 3 molecular types and 37 subtypes of excitatory synapses across more than 100 brain regions, we show that experience-induced changes are globally distributed yet type- and subtype-specific, forming regionally structured patterns in synaptome architecture. These patterns are distinct for different types of sensory experience and are correlated with large-scale connectivity and proteostasis mechanisms. Importantly, we find that this synaptome remodelling operates both during early developmental periods, including classical critical periods, and in the mature adult brain, indicating a conserved but flexible capacity for experience-dependent adaptation in populations of synapses across the lifespan.

EE and MD induced widespread, yet selective, changes in the abundance of excitatory synapse types and subtypes across the brain resulting in a reconfiguration of synaptome architecture. Experience and sensory input induce brainwide neural activity, as evidenced by fMRI, calcium imaging^49–51^ and multiunit recording with neuropixel probes^52–54^. This activity propagates through the brain’s synaptome architecture, engaging excitatory synapse types and subtypes with distinct protein compositions, turnover rates and nanoarchitectures.^29–32,55,56^ The parsimonious explanation for the experience-dependent changes in synaptome architecture is that particular synapse types and subtypes modify their individual proteomes, changing from one type or subtype to another. Because the reconfiguration changes the abundance of PSD95- and SAP102-expressing synapses and these proteins are known to play a key role in controlling synaptic strength, short- and long-term plasticity^29–32,55,56^, this reconfiguration would alter the physiological properties of populations of synapses, which in turn could modify the behavioural responses of the animal. Activity-dependent synaptome reconfiguration could explain the observations that EE and MD modify basal synaptic transmission, short- and long-term plasticity^57,58^, as does alteration of the expression of PSD95 or SAP102^28–30^. Encoding behavioural representations through distributed changes in the synaptome endows the brain with substantial capacity to record experiences.

This capacity for experience- and environment-dependent selective effects on synapse types and subtypes can be viewed as a population-selection model analogous to adaptive processes in evolutionary biology and immunology. In evolution, selective pressures act on genetically diverse populations to shift allele frequencies over generations^59^. In the immune system, antigens selectively expand particular lymphocyte clones from a pre-existing diverse repertoire^60^. Similarly, in the brain, environmental experience and sensory input may selectively act on molecularly distinct synapse populations, altering the relative frequencies of synapse types and subtypes. Unlike genetic selection, which occurs across generations, synaptic and immunological selection operate within the lifespan of an organism and can thereby support rapid adaptation to changing environments.

### Synaptome-connectome-proteostasis nexus

The distributed and selective nature of synaptome remodelling led us to explore its relationship with protein turnover and with the organization of the connectome. The experience-dependent changes in synapse types and subtypes reflect reorganisation of the protein composition and nanoarchitecture of individual synapses. Consistent with a role for mechanisms regulating protein turnover, we found^17^ that the synapse subtypes most affected by EE were characterized by long PSD95 protein lifetimes and high protein concentrations. This suggests that proteostasis may provide one mechanistic context for understanding why some synapse subtypes appear more experience-sensitive than others^61,62^.

In addition to subtype-level proteostatic properties, we asked whether synaptome remodelling showed large-scale spatial alignment with baseline connectome architecture. We found that the spatial distribution of lifetime-defined synapse classes was anatomically structured with the connectome, with the control densities of LPL subtypes showing their strongest associations with sensory pathways. When we examined the SPL and MPL subtypes upregulated in response to MD, we found that the regions receiving strong thalamo-cortical visual inputs, such as the lateral geniculate nucleus and visual cortices, showed larger effect sizes for MD-associated density changes. These MD-induced synaptome changes were more strongly correlated with projection strength than those triggered by EE, which showed an enhancement in existing, control projection-subtype relationships. This dichotomy might reflect the brain’s evolved sensitivity to the multimodal input in EE compared with the more extreme and unnatural manipulation of MD that drives changes in more specific sensory pathways. Furthermore, this contrast underscores the difference between modulating normal sensory experience and responding to its abrupt loss: whereas EE fine-tunes existing synaptic patterns, MD necessitates more radical reconfiguration, echoing known circuit-level rewiring in visual cortex following deprivation^49,63–66^. The capacity to partially decouple from typical projection-subtype alignments allows the engagement of alternate or latent circuits.

### Genetic and experience mechanisms converge

Synaptome architecture is established during development through a hierarchical cascade of genome-encoded molecular mechanisms—including transcriptional, translational, and post-translational processes—that regulate the synthesis and spatial distribution of protein complexes across diverse synapse types within dendritic arbors ^16,17^. Consistent with the central role of genetic programs in shaping this architecture, we have previously shown that mutations associated with intellectual disability and schizophrenia, affecting both transcription factors^22^ and synaptic protein genes^16,17^, produce large-scale alterations in synaptome organisation. Importantly, several levels of this regulatory hierarchy are themselves modulated by neural activity. For example, activity-dependent transcription of immediate-early genes^67^ and regulation of proteostasis^61,68–71^ provide mechanisms through which experience could influence synaptome composition. Such processes are likely to contribute to synaptome remodelling during development, ageing^21,22^, and following sleep deprivation^23^. Together, this hierarchical molecular framework enables the convergence of intrinsic genetic programs and extrinsic environmental influences on the synaptome. The spatial organisation of molecularly heterogeneous excitatory synapses into a dynamic synaptome architecture therefore constitutes a flexible and integrative substrate through which genetic and experiential factors shape brain function.

### Environmental enrichment and deprivation

A growing body of evidence demonstrates that early sensory, cognitive, and physical experiences shape brain development, influence cognitive resilience, and modulate vulnerability to neuropsychiatric disease across the lifespan^72–80^. Against this backdrop, EE paradigms in animal models have frequently been interpreted to support enhanced synaptogenesis as a cellular mechanism underlying experience-driven cognitive and behavioural benefits^33–39^. In the EE paradigms examined here, however, we observed no significant increase in total synapse density across the brain in young or mature animals. Because previous studies examined much smaller numbers of structures in highly localized regions, often using different readouts such as dendritic spines, ultrastructural synaptic contacts, puncta intensity averages, or immunolabelled puncta for other markers, as well as different EE conditions and duration, we cannot exclude the possibility that other EE paradigms might lead to increases in overall excitatory synapse density. Nor can we exclude the possibility that EE-induced synaptogenesis produces excitatory synapses that do not express PSD95 or SAP102 and were therefore not detected in our experiments.

Rather than an overall increase in synapse density, we found that EE restructured the synaptome by downregulating specific subsets of Type 1 and Type 3 synapses across cortical, thalamic, and cerebellar regions. This fine-scale remodelling underscores that EE restructures existing populations of synapses rather than expanding excitatory connectivity, and that cognitive improvements associated with EE likely arise from selective reorganization of synapse populations rather than generalized synapse proliferation.

Synaptome changes were enriched in the deep layers of the cortex and in sensorimotor and subcortical regions, consistent with known enrichment-induced behavioural gains in learning and sensorimotor coordination^24,81,82^. This reorganization was not limited to early development: adult mice exposed to one month of EE displayed overlapping subtype shifts, indicating that the capacity for synaptome reprogramming persists into maturity^64–66^, although the regional focus of plasticity shifted away from deep cortical layers.

The MD paradigm confirmed that modality-specific sensory deprivation can elicit global synaptome remodelling. MD led to brainwide increases in Type 3 synapses, notably in both contralateral and ipsilateral isocortex and hippocampus, as well as in auditory and somatosensory regions. Our findings counter earlier assumptions that MD-induced plasticity is confined to the visual system and instead reveal a cortex-wide redistribution of excitatory synapse subtypes. Consistent with our conclusions, recent evidence showing altered neuronal activity beyond the visual cortex in mice subjected to MD^49^ supports the idea that synaptome changes may underlie broader functional reorganization of brain connectivity.

### Limitations and future perspectives

We selected EE and MD paradigms because they are well-established approaches for inducing activity-dependent changes in the nervous system. The developmental EE paradigm encompasses multiple sensory modalities and includes complex social and maternal interactions and future studies could disentangle the relative contributions of these components. Future studies could also compare Marlau cage and other EE cage configurations. We chose the Marlau system in part because EE protocols have historically been highly heterogeneous, and this standardized commercial design was intended to improve reproducibility within the field while preserving the hallmark effects of EE, including increased cortical thickness, reduced anxiety behaviour and improved learning^83–86^. A limitation of the adult-onset EE experiment is that it was performed in females only, as transfer of adult males into Marlau-cage EE produced marked aggression in pilot work. Sex-specific effects of adult-onset EE therefore remain an important question for future studies using EE paradigms suitable for adult males. However, our developmental EE experiment included both males and females, allowing sex to be assessed as a biological variable in developmental EE, thereby providing a brain-wide analysis of sex differences in synapse-density responses to EE.

For MD, we followed a widely used protocol that did not include sham anaesthesia in control animals. Although the eyelid-suture procedure was brief (<15 min) and tissue was collected 6 days later, we cannot fully exclude the possibility that some of the observed widespread synaptome changes were influenced in part by anaesthesia, surgical stress, recovery-related changes in activity, or other whole-animal procedural effects. A recent MD imaging study that included sham-surgery controls also reported brain-wide effects^49^, suggesting that distributed responses are not unique to the present design. Our contralateral-versus-ipsilateral normalisation, adjusted using internal control data, was adapted from the inter-hemispheric normalisation approaches used in classical MD studies to derive ocular dominance indices^87,88^. This metric isolates deprivation-biased effects and revealed changes across multiple brain regions beyond the visual cortex. However, because it relies on an internal inter-hemispheric comparison, it cannot resolve bilateral changes shared equally across both hemispheres, which therefore remain potentially conflated with procedural effects. While such internal-control approaches are well established for classical, region-focused MD analyses, brain-wide interrogation of both unilateral and bilateral networks would benefit from explicit sham controls. Future experiments incorporating sham-operated controls will therefore be required to distinguish deprivation-specific from procedural contributions more definitively.

While the results of manipulations such as EE and MD are traditionally interpreted in the context of Hebbian synaptic plasticity, our findings do not rule out homoeostatic mechanisms downstream of changes in excitability or inhibition. Secondary changes in the distribution of excitatory synapse types and subtypes might occur if EE/MD produced non-synaptic changes (e.g. changes in action potential thresholds, resting membrane potential). Arguably, this would still be a form of “experience-dependent plasticity”^46,89^, but not of the Hebbian type.

The observed associations between synaptome and connectome may reflect indirect network organisation, shared regional properties, or other unmeasured factors. Nevertheless, they remain biologically informative because they suggest that pre-existing connectome architecture may contribute to the regional sensitivity of synaptome remodelling. To determine whether synaptome changes reflect changes in the connectome, such as new synapse formation and the deletion of pre-existing connections, it will be necessary to obtain condition-specific whole-brain connectome data from EE- or MD-exposed animals, which is currently unfeasible.

Future studies combining measurements of electrophysiological properties at individual synapses with simultaneous identification of synapse type and subtype, across time points before, during, and after EE or MD, will be required to fully resolve the functional properties of synapses reorganised by experience. Experiments examining the stability and reversibility of synaptome architecture, for example by reopening the deprived eye or transitioning animals from EE to SC, will provide further insight into its dynamics. Expanding molecular labelling to additional synaptic proteins will also enable interrogation of the much greater combinatorial diversity of synapses beyond the three types and 37 subtypes analysed here. An important future direction will be to test whether punctum- and subtype-level synaptome features can classify individual animals according to prior experience, sex, or disease state. Such analyses would help determine how different biological conditions are represented at the synaptome level and would provide a useful quantitative test of the distinctiveness of synaptome signatures.

More broadly, synaptome mapping can be applied across diverse learning, sensory and experience-dependent paradigms to determine where in the brain synaptic modifications occur and how they selectively affect synapse types and subtypes. Given that synaptome architecture is altered by gene mutations, including those associated with learning impairments and schizophrenia, this approach provides a framework to investigate how experience interacts with genetic background to generate distinct synaptome architectures.

## Methods

### Animals

#### Mouse lines

Male and female mice used for the comparison of environmental enrichment (EE) and standard cage (SC) during development were of mixed background from crossing C57BL/6 mouse lines expressing *Psd95* ^*eGFP/eGFP*^*;Sap102* ^*mKO2/mKO2*^ with a 129S5 strain expressing *Glun1*^*TAP/TAP*^ ^16,90^. Male and female mice used for monocular deprivation (MD) and control groups were C57BL/6 colonies expressing *Psd95* ^*eGFP/eGFP*^*;Sap102*^*mKO2/mKO2*^. Female mice from both strains were used for the adult EE experiment. The EE animal experiments underwent review by the University of Edinburgh Animal Welfare and Ethical Review Body (AWERB). They were subsequently approved by the UK Animals in Science Regulation Unit (ASRU) under the Animals (Scientific Procedures) Act 1986 and authorised by Project Licence [PPL # PF3F251A9, PP6386243] in strict accordance with the Home Office Code of Practice. The experiments were conducted following an authorised experimental protocol reviewed by both the AWERB and the Bioresearch and Veterinary Services (BVS) department at the University of Edinburgh. The MD experimental procedures were carried out at Cardiff University; they underwent local ethical review and were approved by ASRU (PPL PP9138610); they were carried out in accordance with the UK Animals (Scientific Procedures) Act 1986 and European Commission Directive 2010/63/EU.

#### Monocular deprivation

Mice were housed in standard caging in groups of 2–5 mice per cage. Mice were weaned at P21 and divided into male or female cages. Mice were assigned to undergo MD surgery to achieve balanced male-female ratios for MD and control groups. MD surgery entailed suturing of the right eye from P25-26 to P31. Anaesthesia was induced with 3% isoflurane in 100% oxygen and maintained at 2% isoflurane. An injection of Metacam (1 mg/kg subcutaneous) was given to alleviate post-operative pain. The lid margins of the right eye were trimmed, and the lids sutured shut with three mattress sutures. A topical antibiotic ointment, chloramphenicol, was applied before animals were allowed to recover and were returned to the home cage. Control mice were not subjected to sham anaesthesia or sham surgery. MD animals remained co-housed with their littermate controls throughout the experiment; mice were not switched to single housing after surgery. All animals were checked daily to ensure that the sutured eye remained closed and to minimise differences in handling between MD and control groups. At P31, mice were sacrificed by perfusion. Tissue collection was performed in random order across groups rather than in a fixed condition-specific sequence, so that any transient arousal effects on remaining cage-mates would not be expected to bias one condition systematically over the other. Fixed brains were stored in 30% sucrose solution (w/v in 1× PBS) for up to 6 days at ice-box temperature. Final sample sizes were *n* = 13 for the MD group (7 female) and *n* = 14 for the non-deprived control group (7 female).

#### Environmental enrichment

For the developmental enrichment study, mice were bred in-house to generate litters for enriched environment (EE) or standard condition (SC) housing. Pregnant dams were distributed 7 days before parturition into EE (*n* = 4 per cage) or SC (*n* = 2 per cage) cages to facilitate social enrichment. Litters remained undisturbed until postnatal day 5. At weaning (P19–P28), pups were sex-separated and housed according to their assigned condition. EE mice were housed in groups of 12–13 per sex-matched cage; SC mice were housed in groups of 2–7, across multiple sex-matched cages. Ear-notching enabled litter identification. Dams were removed at weaning. Mice were perfused between P81 and P96. Final sample sizes were EE *n* = 23 (12 female), SC *n* = 29 (15 female). This time window was chosen because previous lifespan synaptome mapping showed that synaptome architecture continues to undergo marked developmental change up to approximately 3 months of age, after which it becomes comparatively more stable^21^. We therefore reasoned that EE rearing through this interval would maximize the likelihood of detecting effects on the developing synaptome architecture.

For the adult enrichment study, a separate cohort of female C57BL/6J mice (aged 6–12 months) were randomly assigned to either enriched or standard housing for 1 month prior to perfusion. EE mice (*n* = 20) and SC mice (*n* = 20) were housed in same-sex groups under the conditions detailed below. Male mice were excluded due to the aggression typically observed upon late transfer into group-housed enriched environments. The adult EE exposure was applied within the 3–12 month age window, during which previous lifespan synaptome mapping indicates that synaptome architecture is relatively stable^21^. This allowed us to test whether 1 month of EE could induce synaptome remodelling outside the major developmental phase of synaptome change.

EE mice were housed in Marlau cages (Viewpoint, Lyon, France) following established protocols^83^. These cages provide a two-level structure including a living area with three running wheels, a red plastic shelter, and access to food through a maze that was reconfigured every two days from twelve preset designs. SC mice were housed in standard open-top cages (floor area: 501 cm²) with a cardboard shelter and clear plastic tunnel for handling. Both EE and SC cages received the same bedding and nesting material. Food and water were provided ad libitum, and all mice were kept under a 12 h light/dark cycle. Cages were cleaned weekly. Bedding and nests were partially transferred between old and new cages to maintain familiar olfactory cues. EE mice were temporarily transferred to standard cages during cage cleaning. All mice were handled using plastic tunnels, and handling was restricted to two experimenters to minimise variability.

### Sample preparation

#### Tissue collection and processing

Mice were deeply anaesthetised via intraperitoneal injection of sodium pentobarbital (20% w/v; Pentobarbitone Sodium, Animalcare; dose: 0.05–1.5 mL according to body size). Upon loss of pedal reflexes, mice underwent transcardial perfusion with 10 mL of phosphate-buffered saline (PBS, 1X; Oxoid), followed by 10 mL of 4% paraformaldehyde (PFA; Alfa Aesar) in PBS.

Brains were dissected and post-fixed in 4% PFA at 4 °C for 3–4 h, then cryoprotected in 30% sucrose (w/v in PBS) at 4 °C for 3–6 days. Once equilibrated, brains were embedded in optimal cutting temperature (OCT) compound (CellPath) within cryomolds, snap-frozen in liquid nitrogen-cooled isopentane, and stored at −80 °C until sectioning.

#### Sectioning

Frozen brains were sectioned at 18 μm thickness using a cryostat. Sections were collected in either the coronal or parasagittal plane depending on the study. For the monocular deprivation experiment, coronal sections were collected from Bregma −3.07 mm to −3.27 mm (corresponding to images 85–86 of the Allen Mouse Brain Atlas). For synaptome mapping in enriched and standard cage mice, parasagittal sections were collected centred on the region corresponding to image 11 of the Allen Mouse Brain Atlas sagittal reference^47^. Two adjacent sections were mounted per slide (Superfrost Plus, Thermo Scientific), dried overnight at room temperature and then stored at −20 °C until imaging.

#### Mounting for imaging

Slides were thawed at room temperature for 5 min before mounting. Sections were rehydrated in 1X PBS (Sigma-Aldrich) for 5 min in a light-protected humidity chamber. A volume of 12 μL of homemade Mowiol mounting medium (Calbiochem) was applied per section. Coverslips (13 mm or 18 mm × 1.5 mm; VWR International) were selected based on section orientation (coronal or parasagittal, respectively). Mounted slides were dried overnight in the dark at room temperature. For short-term use (<5 days), slides were stored at 4 °C; for longer-term storage, they were kept at −20 °C.

### Imaging and data acquisition

Whole-brain section imaging was performed using a Nikon Eclipse Ti2 inverted microscope equipped with a Yokogawa CSU-W1 spinning-disk confocal scanner and a Photometrics Prime BSI camera. Image acquisition was controlled via Nikon NIS-Elements software (v5.20.1–5.21.1). Each slide was first scanned at 4× magnification (NA 0.2) to acquire an overview of the brain sections and define coordinates for high-resolution imaging. Whole-section imaging was then performed at 100× magnification (NA 1.45, oil immersion), using automated tile scanning protocols with consistent acquisition settings across all experiments.

For high-magnification imaging of PSD95-eGFP and SAP102-mKO2, the 561 nm laser (SAP102-mKO2) was triggered first, followed by the 488 nm laser (PSD95-eGFP). Excitation was delivered through the L100 light path using emission filter 1. Laser power and exposure times were set to 2.33 mW for 200 ms (561 nm) and 1.06 mW for 120 ms (488 nm), respectively. Aperture was set to 1, and imaging was performed in 12-bit with Correlated Multi-Sampling (CMS) gain.

For low magnification 4× overview imaging, laser powers were reduced to minimise photobleaching: 0.61 mW for 561 nm (200 ms) and 0.71 mW for 488 nm (120 ms), with aperture set to 7. The same light path (L100), emission filter (1), gain (CMS), and 12-bit resolution were used as in the high-magnification protocol.

Each whole-brain scan contained over 10,000 tiles. Autofocus correction was performed every 400 tiles using a z-stack of 41 optical sections (0.5 µm step size). Final z-positions were calculated from the midpoint intensity value of the stack to account for focal drift along the z-plane. Autofocus points were manually selected in regions of high synaptic density.

For the adult EE dataset, we used a subsampled variant of the synaptome mapping imaging protocol to increase throughput. Instead of contiguous rectangular tiling, high-magnification (100×) imaging was performed on a systematic sparse-sampling grid: fields of view were acquired at regular offsets in X and Y with a centre-to-centre step equal to 1.5× the field of view, yielding non-overlapping images separated by half a field. Polygonal ROIs were defined from the 4× overview as above, and the grid was applied uniformly within each ROI. All other optical and acquisition parameters and the autofocus procedure matched the whole-section protocol, providing a balanced compromise between mapping speed and spatial precision.

All scans were acquired using automated JOBS routines in NIS-Elements, incorporating the optical configuration settings, autofocus correction, polygonal ROI assignment, and coordinate interpolation. Imaging settings were validated using saturation and signal-to-noise ratio (SNR) analyses. SNR was optimised using TrackMate in ImageJ (v2.0.0) with custom MATLAB scripts (available on request). To minimise variability in image background and punctum detectability between imaging sessions, all scans were acquired using this standardised automated workflow with fixed optical settings, including laser power, exposure time, aperture, emission filter, gain, bit depth, and autofocus procedure. Thus, high-magnification imaging was performed under matched acquisition settings across experiments, with pre-acquisition optimisation of saturation and SNR before large-scale data collection.

### Image processing and brain region delineation

Following whole-brain section imaging, raw image files were processed using Nikon NIS-Elements Advanced Research software (v5.21). Image metadata and individual 856 × 812-pixel tiles were extracted for each section. To evaluate image quality and anatomical positioning, upscaled overview montages were generated using custom MATLAB scripts (R2020b–R2022a; scripts available upon request). These montages were reconstructed using the extracted tiles and metadata, enabling visual inspection for imaging artefacts, tissue damage, and scan alignment along the anteroposterior and mediolateral axes. Scans with extensive out-of-focus areas—primarily due to drift in the nosepiece z-plane—were excluded and repeated using adjacent sections.

Montage images from accepted scans were used for manual delineation of brain regions, guided by the Allen Mouse Brain Atlas^47^. One imaged section was used per mouse for delineation and subsequent analysis. Delineations were performed in ImageJ (v2.14.0) using the polygon ROI tool and ROI Manager. Only visually distinguishable subregions corresponding to the reference atlas image were included. For manual delineations, synaptic marker expression (PSD95-eGFP and SAP102-mKO2), cell body density, and laminar organisation were used in conjunction with atlas references to guide boundary placement. In the monocular deprivation study, additional reference was made to cortical layer 4 morphology to distinguish visual subfields^91^. All delineated regions used in analysis are listed in the supplementary materials (Supplementary Figs. S31, S32). While anatomical landmarks and synaptic marker patterns were used to approximate regional boundaries, it is acknowledged that these may not precisely reflect functional or synapse-specific zones.

Manual brain region delineation was performed for the MD and developmental EE studies. For the adult EE study, an automated delineation tool developed in-house was employed, which had been trained using the manual delineations from existing datasets^92^. Automated delineations of each section underwent visual QC. When a boundary showed a clear deviation from the atlas or from our manual delineation criteria, the affected ROI mask PNGs were imported into napari (v0.5.5) as label layers and corrected with pixel-level paint/erase tools to realign boundaries to cytoarchitectonic landmarks and synaptic marker patterns. Edits were sparse and local: many sections required no changes, while others required adjustments to fewer than 15 subregions, most commonly laminar boundaries in PTLp and cerebellar lobules. Corrected masks were exported as updated PNGs and used for downstream ROI assignment and quantification.

As a quality-control step, overview montages and extracted regional measurements were inspected across animals to confirm broadly consistent PSD95 and SAP102 signal patterning despite expected biological inter-animal variation. We did not observe evidence of gross batch-specific distortion of the expected regional synaptome patterning, and the consistency of these region-wise patterns across animals supported inclusion of all samples in downstream analyses.

### Synaptome mapping: quantification of synapse parameters

#### Punctum detection

To identify PSD95-eGFP and SAP102-mKO2 synaptic puncta, we used a custom machine learning-based detection algorithm adapted from previously validated synaptome mapping pipelines^16,21^. Detection was performed independently for the PSD95 and SAP102 channels. Fluorescently tagged puncta were detected using a multi-scale, multi-orientation feature extraction framework combined with an ensemble-trained neural network classifier. To adapt the synaptome mapping detection algorithm for use with the Nikon Ti2 Eclipse spinning-disk confocal system, we generated a new training dataset comprising z-stacks (5 planes, 150 nm spacing) from parasagittal brain sections of *Psd95*^eGFP/eGFP^; *Sap10*2^mKO2/mKO2^ mice aged P7, P14, 3 months, and 1 year (*n* = 3 per age group). Randomised 128 × 128 pixel sub-tiles (*n* = 1000) were extracted and manually annotated using the ImageJ cell counter plugin by five independent raters blinded to study aims. Puncta were defined as bright, round or elliptical spots, and annotations were minimally guided to reduce selection bias. These manual annotations were used as training labels for the classifier. Candidate puncta were extracted using local image features, then classified as true synaptic puncta or background based on their feature vectors. Final outputs included the coordinates and extracted properties for each detected punctum. Code and trained classifiers are available on GitHub (see Key Resources Table).

#### Punctum segmentation and parameter extraction

Following detection, each synaptic punctum was segmented using a local thresholding approach based on 20% of its peak intensity, analogous to full width at half maximum (FWHM). This allowed separation of size and intensity measurements. A marker-controlled watershed algorithm was applied to resolve overlapping puncta. Puncta smaller than 4 pixels were excluded. For each segmented punctum, six parameters were calculated: mean intensity, area, circularity, aspect ratio, skewness, and kurtosis. Shape descriptors quantified the asymmetry, peakedness, and roundness of each punctum. Parameter extraction was performed using MATLAB’s *regionprops* function and custom scripts. This step enabled downstream synapse classification based on both molecular composition and morphological features.

#### Punctum colocalisation

To determine which puncta co-expressed PSD95 and SAP102 at the same synapse, we performed a spatial colocalisation analysis based on Euclidean distance between punctum centroids^16^. Pairs separated by ≤500 nm were classified as colocalised. This threshold was selected based on both synaptic ultrastructure^93^ and dual-peaked histograms of punctum distances. Due to the diffraction limit, only one punctum per protein is expected per synapse, so a one-to-one matching constraint was imposed and modelled as a linear assignment problem, solved using the Hungarian algorithm. Colocalisation was performed after independent detection and segmentation of both channels.

#### Punctum classification

To quantify synapse diversity, detected puncta were classified within the previously established synaptome framework^16^, in which excitatory synapses are organized hierarchically into major synapse types and finer subtypes. At the first level, synapses were assigned to one of three major molecular classes according to marker composition: PSD95-positive only, SAP102-positive only, or colocalised PSD95-positive/SAP102-positive puncta. At the second level, puncta within these major classes were assigned to one of 37 previously defined synapse subtypes using quantitative image features^16^.

The original 37-subtype system was derived from unsupervised weighted ensemble clustering of puncta acquired on the Andor synaptome imaging^16^, based on molecular composition together with punctum intensity, size, and shape descriptors. In the present study, we did not redefine the subtype taxonomy. Instead, we adapted this existing classification framework for Nikon-acquired images using transfer learning, such that Nikon-imaged puncta could be mapped onto the established subtype space while preserving compatibility with prior synaptome studies.

Three separate classifiers were used, corresponding to PSD95-only puncta, SAP102-only puncta, and colocalised PSD95/SAP102 puncta. Classification was based on punctum-specific features comprising mean intensity, area, skewness, kurtosis, circularity, and aspect ratio. The original classifier architecture was retained, and selected model layers were fine-tuned on Nikon-imaged puncta using transfer learning. Final classifiers were implemented in MATLAB as Compact Classification ECOC models. For each punctum, the classifier returned a subtype label together with a confidence score using the built-in MATLAB predict function. Puncta with classification confidence <10% were excluded from downstream analyses. All trained classifiers are available on GitHub (see Key Resources Table).

#### Brain mapping

Following detection, colocalisation, and classification, synaptic parameters were aggregated to anatomical brain regions. First, punctum-level features were averaged within 4 × 4 subtiles per image tile. These subtile-level summaries were then mapped to anatomical subregions delineated using the Allen Mouse Brain Atlas^47^. Regional values (e.g., intensity, size, shape, subtype density) were computed by averaging across all subtiles within each delineated mask. Puncta exceeding 140 pixels in size or with classification confidence <10% were excluded. Puncta on tile edges were also excluded to reduce border artefacts. Final outputs for each region included summary synapse parameters for individual synaptic puncta and average densities of the 37 assigned synapse subtypes. Subtype density values are reported per 4 × 4 subtile (~200 µm²). Punctum size is reported in pixels, and punctum intensity is reported in arbitrary fluorescence units.

Because subtype labels were assigned using a common predefined classification framework applied consistently across all samples, the resulting subtype densities could be compared across animals, brain regions, and experimental conditions.

### Statistical analyses

#### Data handling

Raw TIFF images, associated metadata, and delineated ROI files were stored and archived on institutional secure servers. Processed outputs from the SYNMAP and TrackMate pipelines—including punctum parameters, colocalisation metrics, and region-level summary values—were exported as tabulated files (e.g.,.csv/.xlsx) and analysed using MATLAB (R2020b–R2022a), R (v4.4.0) and Python (3.13 for connectome analyses; Python ≥3.8 required for the SYNMAP pipeline). For each animal, punctum and synapse subtype data were aggregated by anatomical subregion using an Allen Mouse Brain Atlas-based parcellation^47^. Group n values reported in the main text reflect the number of animals unless otherwise indicated. In some cases, subregions do not include data from all animals because the small size and anatomical delineation of certain areas precluded averaging in the SYNMAP pipeline, or because those regions were excluded during delineation; subregion-level n are provided in the Source Data. Bayesian statistical analysis was performed using adapted open-source MATLAB and R code (see Key Resources Table), implemented in JAGS via runjags/matjags. Figures were generated using MATLAB, RStudio and an in-house brain heatmap plotting tool (see Key Resources Table); owing to the granularity of the Allen atlas reference maps, the primary visual cortex region in the coronal heatmap tool reflects binocular zone data only. Across brain regions and synapse subtypes, we report effect sizes (Cohen’s *d*) as the primary summary statistic alongside a posterior-probability–derived ‘*p*-value proxy’ as described; posterior draws underlying this proxy are deposited (Key Resources Table).

#### Bayesian analysis

To assess experience- and sex-dependent differences in synapse parameters across brain subregions, we implemented robust Bayesian estimation models adapted from Kruschke’s hierarchical framework, consistent with previous synaptome mapping studies^16,17,21–23,94,95^. Analyses were performed separately for each synapse parameter within each brain subregion, with individual animals as the unit of observation. Separate models were fit for each region–parameter combination for the MD, developmental EE, and adult EE datasets, always relative to the matched control group.

#### Two-group comparisons

For comparisons involving only experimental versus control animals (e.g. adult EE), Bayesian estimation was used as a principled alternative to t-tests. Observations (y_i) were modelled using a Student’s *t*-distribution with group-specific means (μ₁, μ₂), group-specific standard deviations (σ₁, σ₂), and a shared degrees of freedom parameter (ν) to accommodate potential outliers. Priors for group means were normal distributions centred on the pooled sample mean and scaled to the observed data range, following the Kruschke framework. Priors on the group standard deviations were gamma distributions parameterized from empirical variance estimates in the corresponding dataset and the ν parameter followed an exponential prior with rate 1/30 and lower bound 1. Posterior samples were obtained using MCMC via matjags in MATLAB, using multiple independent chains, 2000 burn-in iterations per chain, thinning by 5, and 15,000 retained posterior samples in total across chains. Evidence for a group difference was assessed from the posterior distribution of the contrast (µ₁–µ₂).

#### Factorial comparisons

To assess condition, sex, and condition-by-sex interaction effects (for example, MD and developmental EE), we used a Bayesian two-way ANOVA implemented in JAGS via the runjags package in R. Observed values yₖ were modelled as yₖ ∼ t(μₖ, σ²₍ⱼ₁,ⱼ₂₎, ν), where μₖ comprised additive and interaction terms corresponding to sex, condition, and their interaction. Residual variability was allowed to differ across condition-by-sex cells through cell-specific scale parameters σ₍ⱼ₁,ⱼ₂₎. The baseline mean and effect terms were assigned weakly informative normal priors scaled to the observed data, and the cell-specific scale parameters were modelled hierarchically using gamma priors whose hyperparameters were themselves estimated from the empirical distribution of group-level standard deviations, following the Kruschke framework. Posterior draws were reparameterized post hoc into sum-to-zero contrasts (b₀, b₁, b₂, b₁b₂) for interpretation of main and interaction effects. Models were fit in JAGS via runjags (R) using MCMC with 4 chains, 2000 burn-in iterations per chain, thinning by 5, and 15,000 retained posterior samples in total across chains.

#### Posterior inference and significance estimation

For each contrast of interest, evidence for a group difference was assessed from the posterior distribution of that contrast (for example, *μ*1 − *μ*2), simple effects, or interaction contrasts. Posterior summaries included measures of central tendency and highest density intervals (HDIs). To assess whether a contrast was credibly different from zero by more than a small effect, we used a Region of Practical Equivalence (ROPE) centred on zero together with an HDI-based exclusion procedure^16^.

The ROPE was defined using the model-estimated residual standard deviations^16^. In the two-group models, this was based on the estimated standard deviations of the two groups being compared. In the factorial models, this was based on the relevant ySigma terms for the condition/sex cells contributing to the contrast. These sigma values were combined to give a single summary standard deviation for that comparison, and the ROPE was then defined as ±σ*/3, where σ*is that summary value. This means that the ROPE represents a small-effect threshold relative to the variability of the underlying data.

To derive a single evidence metric for each contrast, we identified the widest HDI that excluded the ROPE and recorded the posterior mass outside that interval. Smaller values indicate stronger evidence that the contrast lies outside the ROPE. For convenience in downstream multiple-comparison correction, this quantity was treated as a p-like metric. However, it should be interpreted as an HDI–ROPE exclusion measure derived from the posterior distribution, rather than as a frequentist *p*-value.

#### Multiple comparisons correction

Because each synapse subtype or parameter was tested across many anatomical regions, we controlled for multiplicity using Benjamini–Hochberg false discovery rate correction (FDR = 0.05). For each contrast and synapse parameter, correction was applied across all analysed brain subregions (97 for adult EE, 101 for developmental EE, and 256 for MD), as in previous synaptome mapping studies^16,17,21–23^. This approach was chosen to preserve power for detecting spatially localized effects while limiting false discoveries across the large number of regional comparisons. We note that anatomical regions are not statistically independent, so this correction should be interpreted as a pragmatic and conservative benchmark rather than an exact representation of anatomical dependency structure.

### Derived metrics and statistical calculations

#### Synapse type and total synapse densities

For each mouse and brain subregion, synapse densities were computed by summing the subtype-level densities from the synaptome mapping pipeline. Synapse type densities were computed by summing the densities of their corresponding subtypes: Type 1 (PSD95-only, subtypes 1–11), Type 2 (SAP102-only, subtypes 12–18), Type 3 (colocalised, subtypes 19–37), and total synapse density (subtypes 1–37).

#### Subtype-level PSD95 protein lifetime annotation and lifetime-class analyses

To test whether experience-associated synaptome remodelling was related to previously defined subtype-level proteostatic properties, we annotated each PSD95-containing synapse subtype with PSD95 protein lifetime estimates reported in a prior synaptome protein-turnover study^17^. In that study, subtype-specific average PSD95 lifetimes were derived across animals and brain regions and were found to be broadly reproducible across the synaptome architecture.

In the present study, these previously reported subtype-level lifetime estimates were mapped onto the corresponding PSD95-containing Type 1 and Type 3 synapse subtypes identified in our dataset. This yielded lifetime annotations for 30 PSD95-positive subtypes. For visualization, subtypes were ordered according to these prior PSD95 lifetime estimates and grouped into short protein lifetime (SPL), medium protein lifetime (MPL), and long protein lifetime (LPL) classes, as defined previously^17^.

These lifetime annotations were not re-estimated in the present experiments and should therefore be interpreted as prior subtype-level properties used to test whether experience-associated changes in subtype abundance were related to previously measured proteostatic characteristics.

#### Effect size estimation (Cohen’s d)

Cohen’s *d* was calculated to quantify the standardized magnitude of the difference between groups, relative to within-group variability, using the pooled standard deviation formula:

$$d=\frac{{\bar{x}}_{1}-{\bar{x}}_{2}}{{s}_{{pooled}}},{where}\,{s}_{{pooled}}=\sqrt{\frac{\left({n}_{1}-1\right){s}_{1}^{2}+({n}_{2}-1){s}_{2}^{2}}{{n}_{1}+{n}_{2}-2}}$$

Here, x_1_ and x_2_ are group means, s_1_ and s_2_ are standard deviations, and n_1_, n_2_ are group sample sizes. We used Cohen’s *d* to facilitate comparison of effect magnitude across brain regions and synapse parameters that differ substantially in their absolute scales and units. In this context, larger absolute values indicate stronger separation between groups relative to inter-animal variability. This standardized representation has been used in previous synaptome mapping studies, enabling consistency across analyses.

#### Cortical area normalisation for heatmaps

To visualize relative changes across cortical laminae, each subtype-layer effect size within a cortical area was normalized by dividing by the maximum observed effect size across all layers and subtypes of that area.

#### Inter-hemisphere difference and normalised contralateral indices

To quantify lateralized responses to monocular deprivation (MD), the following metrics were computed for each punctum and subtype parameter:

Asymmetry Index:

$${Asymmetry}=\frac{\left|{Contralateral}\right.-\left.{Ipsilateral}\right|}{{Contralateral}+{Ipsilateral}}$$

Normalised Contralateral Index:

$${Normalised\; Contralateral}=\frac{{Contralateral}}{{Contralateral}+{Ipsilateral}}$$

Both metrics were computed per individual and averaged at the group level. The asymmetry index captures magnitude of lateralisation, while the normalised index reflects directional bias.

#### Similarity matrices

To compare which subregions and subtypes were similarly affected by environmental enrichment, monocular deprivation, or between enrichment paradigms, Pearson’s correlation similarity matrices were generated for Cohen’s *d* effect size subregion x subtype matrices.

#### PCA-clustering analysis

The input to this analysis was a subtype-by-region matrix of Cohen’s *d* values. Rows corresponded to synapse subtypes and columns corresponded to anatomical subregions, so each subtype was represented by its brainwide effect-size profile. Cohen’s *d* effect size values were computed for each of 37 synapse subtypes across all anatomically defined brain subregions in each experimental condition (MD vs. non-deprived control; EE vs. SC control). Prior to analysis, variables were centred and scaled. Assumptions regarding normality, multicollinearity, and linearity were assessed; robust methods were used where appropriate. Robust PCA was performed using the PcaHubert() function (R package rrcov, v1.6), which reduces sensitivity to outliers and non-Gaussian distributions. The number of retained principal components was determined by visual inspection of scree plots and bootstrapped confidence intervals (1000 resamples). PCA scores were subjected to agglomerative hierarchical clustering using Ward’s method. The optimal number of clusters was identified via the NbClust consensus method. Cluster robustness was assessed using Jaccard stability indices (bootstrap resampling, 1000 iterations), and Kruskal–Wallis tests were used to assess significant separation between clusters along principal components. Clusters therefore indicate groups of subtypes with similar anatomical response profiles within a given paradigm and were not used to redefine the original synapse subtype classification. All analyses were conducted in R.

#### Spearman’s correlation of subtype parameters

To examine whether subtype responsiveness to environmental manipulation was related to previously defined subtype properties, Spearman’s rank correlations were computed between subtype-level Cohen’s *d* effect sizes and the following subtype annotations: PSD95 protein lifetime, protein concentration, protein amount, and synapse size. Analyses were restricted to the 30 PSD95-positive Type 1 and Type 3 synapse subtypes for which prior PSD95 lifetime estimates were available^17^.

Protein lifetime values were taken from the subtype-level annotations described above. Protein concentration was estimated as the mean pixel intensity of PSD95 puncta, punctum size was defined by pixel area, and protein amount was calculated as the product of mean intensity and area. Correlations were computed using Spearman’s rank method (ρ) via the corr function in MATLAB (R2022a). In these analyses, the prior lifetime estimates were used as subtype-level explanatory annotations and were not directly measured in the current experiments.

#### Connectivity correlation analysis

To investigate whether mesoscale axonal connectivity constrains synaptome organisation and plasticity, we correlated synapse subtype metrics with anatomical projection strengths obtained from the Allen Mouse Brain Connectivity Atlas^48^. Projection data were sourced from 129 tracer injection experiments in wild-type mice, each reporting projection densities from a single source region to multiple annotated brain targets. A metadata.csv file and associated per-experiment XML files were downloaded manually from the Allen portal using a custom Python script. XML data were converted to readable format using an R script provided by the Allen Institute.

Projection density values, injection volumes, experiment labels, and region labels from each injection experiment were extracted using custom MATLAB scripts. Projection densities were normalised by the injection volume per experiment. Where multiple experiments targeted the same source region, values were averaged to obtain a representative profile.

To align our synaptome-mapped brain subregions with available connectome targets, we manually matched delineated anatomical regions to Allen-defined targets using the Allen Reference Atlas^47^. Regional groupings (e.g., cortical layers, visual subfields) were averaged where needed to harmonise naming across datasets. This mapping step involved averaging synapse metrics across some adjacent subregions where the connectome dataset did not distinguish between them. Separate connectome matrices were produced for the monocular deprivation (MD) dataset (coronal sections; 91 target regions) and the environmental enrichment (EE) dataset (sagittal sections; 73 target regions) due to differences in imaging coverage.

For each experiment, baseline synapse subtype densities (mean of control animals) and absolute Cohen’s *d* effect sizes were compiled into 37 × N matrices, where N is the number of matched target regions per dataset. These matrices were then correlated against a normalised 129 × N matrix of projection densities using Spearman’s rank correlation (MATLAB corr function, ‘Type’,‘Spearman’). P-values were derived using MATLAB’s default large-sample approximation for Spearman’s rho, and multiple comparisons were corrected using the Benjamini–Hochberg method (built-in MATLAB function). Correlations with adjusted p-values below the critical threshold were classed as significant.

#### Clustering heatmap

These heatmaps were used as visual summaries of high-dimensional correlation profiles, analogous to clustered heatmaps commonly used in transcriptomic or proteomic analyses. We clustered correlation matrices relating projection strength to either baseline subtype density (ρᴰ) or experience-induced effect size (ρᶜᵈ). Each matrix (subtypes × source regions) was z-scored across projection regions to normalise subtype profiles. Z-scoring was applied for visual comparison of correlation patterns across source regions and does not alter the underlying Spearman correlation coefficients reported in the analyses. Hierarchical clustering was applied to both rows and columns using Pearson correlation distance and average linkage. Optimal leaf ordering was used to enhance cluster visualisation. Heatmaps were generated in MATLAB using the reordered matrices and labelled axes to identify synapse subtypes with similar projection correlation patterns.

#### T-SNE and UMAP analysis

To visualise projection associations across synapse subtypes, we applied dimensionality reduction to matrices of Spearman’s rank correlation coefficients relating projection strength to either baseline subtype density (ρᴰ) or enrichment- or deprivation-induced effect sizes (ρᶜᵈ). Each region was standardised across synapse populations using the scale() function in R (z-scoring to zero mean and unit variance), so that all regions contributed comparably to the analysis. We used both t-distributed Stochastic Neighbour Embedding (t-SNE) from the Rtsne package in R and Uniform Manifold Approximation and Projection (UMAP) from the uwot package in R, using Euclidean-based distance metrics. For t-SNE, perplexity values from 5 to 12 were tested; for UMAP, the number of nearest neighbours varied from 5 to 12. Parameter optimisation was guided by Mantel correlations between high- and low-dimensional distance matrices, reflecting global structure preservation. To evaluate local fidelity, we calculated trustworthiness and k-nearest neighbour overlap (k = 5) between high-dimensional and embedded spaces. Embedding stability was assessed by performing ten replicate runs and measuring alignment consistency via Procrustes analysis. Final embeddings used in figures were generated using a perplexity of 12 for t-SNE, and 10 (1 M/MD) or 8 (3 M/EE) nearest neighbours for UMAP. For t-SNE, the most representative embedding was selected as the run with the lowest average Procrustes residual across replicates, and this solution was used in final MATLAB visualisations. t-SNE and UMAP were used for visualisation of subtype relationships in high-dimensional correlation space, not for statistical hypothesis testing. Biological interpretation was based on recurring subtype groupings observed across PCA, hierarchical clustering, t-SNE/UMAP visualisation, and the underlying effect-size or correlation matrices.

#### Anatomically structured edge bundling of synapse–projection relationships

To visualise how experience-dependent synapse plasticity integrates with the brain’s mesoscale connectivity, we constructed a hierarchical bundling graph in a circular dendrogram layout. Nodes represented brain subregions, grouped into major anatomical domains. Edges represented projection strength between source and target regions based on connectivity data from the Allen Mouse Brain Connectivity Atlas. Projection strength and Spearman’s correlations were combined to generate a weighted edge list. Only statistically significant connections (*p* ≤ critical *p*) were retained, and the top 500 strongest source–target pairs were visualised. A custom MATLAB pipeline processed, filtered, and ranked edges based on projection strength and correlation magnitude (see Key Resources Table). The final node parameter values (summed correlation strengths) were computed per subregion for available target region data. Graph layout and plotting were performed in R using the ggraph and igraph packages. The hierarchy was defined by parent–child relationships between anatomical domains and subregions, with an artificial central “origin” node anchoring all domains. Subregion nodes were coloured by anatomical domain using a custom colour map.

**Key Resources Table**

**Table:** 

Chemicals, peptides, and recombinant proteins		
OCT embedding matrix	Cell Path	#KMA-0100-00A
Deposited data		
Allen mouse brain reference atlas	Allen Reference Atlas – Mouse Brain	http://mouse.brain-map.org/static/atlas
Mesoscale structural connectome	Allen Mouse Brain Connectivity Atlas	http://connectivity.brain-map.org/
Main figure data	This paper	Source Data
Supplementary Fig. data	This paper	Source Data Supplementary Figs.
Raw imaging data and synaptome mapping output data	BioStudies	https://doi.org/10.6019/S-BSST2147 https://ftp.ebi.ac.uk/biostudies/fire/S-BSST/147/S-BSST2147/Files/
Brain region list with abbreviations	This paper	Supplementary Material
Posterior distributions (MCMC draws)	BioStudies	https://doi.org/10.6019/S-BSST2147 https://ftp.ebi.ac.uk/biostudies/fire/S-BSST/147/S-BSST2147/Files/
PCA scores and loadings	BioStudies	https://doi.org/10.6019/S-BSST2147 https://ftp.ebi.ac.uk/biostudies/fire/S-BSST/147/S-BSST2147/Files/
Projection matrix (source × target, normalised)	BioStudies	https://doi.org/10.6019/S-BSST2147 https://ftp.ebi.ac.uk/biostudies/fire/S-BSST/147/S-BSST2147/Files/
Synaptome-connectome region mapping table	BioStudies	https://doi.org/10.6019/S-BSST2147 https://ftp.ebi.ac.uk/biostudies/fire/S-BSST/147/S-BSST2147/Files/
Edge list for bundled graph (source, target, weight, correlation)	BioStudies	https://doi.org/10.6019/S-BSST2147 https://ftp.ebi.ac.uk/biostudies/fire/S-BSST/147/S-BSST2147/Files/
Software and code		
MATLAB	MathWorks	https://uk.mathworks.com/?s_tid=gn_logo
Python	Python Software Foundation	https://www.python.org/
R for statistical computing	R projects	https://www.r-project.org/
ImageJ	NIH	https://imagej.net/ij/
Bayesian estimation	BioStudies	https://doi.org/10.6019/S-BSST2147 https://ftp.ebi.ac.uk/biostudies/fire/S-BSST/147/S-BSST2147/Files/
Just Another Gibbs Sampler	SourceForge	https://sourceforge.net/projects/mcmc-jags/files/
Brain map plotting tool	Git	https://git.ecdf.ed.ac.uk/ddominic/the-synaptome-experience-project/-/blob/main/README.md
Synaptome mapping pipeline	Git	https://git.ecdf.ed.ac.uk/ddominic/the-synaptome-experience-project/-/blob/main/README.md
PCA + hierarchical clustering	BioStudies	https://doi.org/10.6019/S-BSST2147 https://ftp.ebi.ac.uk/biostudies/fire/S-BSST/147/S-BSST2147/Files/
Connectivity correlation	BioStudies	https://doi.org/10.6019/S-BSST2147 https://ftp.ebi.ac.uk/biostudies/fire/S-BSST/147/S-BSST2147/Files/
Dimensionality reduction (t-SNE, UMAP)	BioStudies	https://doi.org/10.6019/S-BSST2147 https://ftp.ebi.ac.uk/biostudies/fire/S-BSST/147/S-BSST2147/Files/
Edge bundling visualisation (circular dendrogram)	BioStudies	https://doi.org/10.6019/S-BSST2147 https://ftp.ebi.ac.uk/biostudies/fire/S-BSST/147/S-BSST2147/Files/
Allen API parsing and XML preprocessing	BioStudies	https://doi.org/10.6019/S-BSST2147 https://ftp.ebi.ac.uk/biostudies/fire/S-BSST/147/S-BSST2147/Files/
Other		
Paper resources	Git	https://git.ecdf.ed.ac.uk/ddominic/the-synaptome-experience-project/-/blob/main/README.md

### Reporting summary

Further information on research design is available in the Nature Portfolio Reporting Summary linked to this article.

## Supplementary information

Supplementary Information

Reporting Summary

Transparent Peer Review file

## Source data

Source Data

## Data Availability

Raw imaging data, synaptome mapping output data and processed intermediate files supporting the findings of study, as well as analysis scripts, have been deposited in the BioStudies database under accession code S-BSST2147. Source data for main Figs. 1–4 and Supplementary Figs. S4–S9, S12, S15–S22, S27–30 are provided as a Source Data file. Source data are provided with this paper.

## Code availability

Code developed for synaptome mapping and analyses outlined in this study are available in the repositories cited in the Key Resources Table.

## Materials availability

This study did not generate new unique reagents.
