# Supplementary material for: Experience modifies the synaptome architecture in the developing and adult mouse brain
Source: Nat Commun. 2026 Nov 11;17:11204. doi: 10.1038/s41467-026-78702-7 (PMC13754844; doi:10.1038/s41467-026-78702-7)
Supplement: Supplementary file 1 [file 41467_2026_78702_MOESM1_ESM.pdf]

Supplementary Information

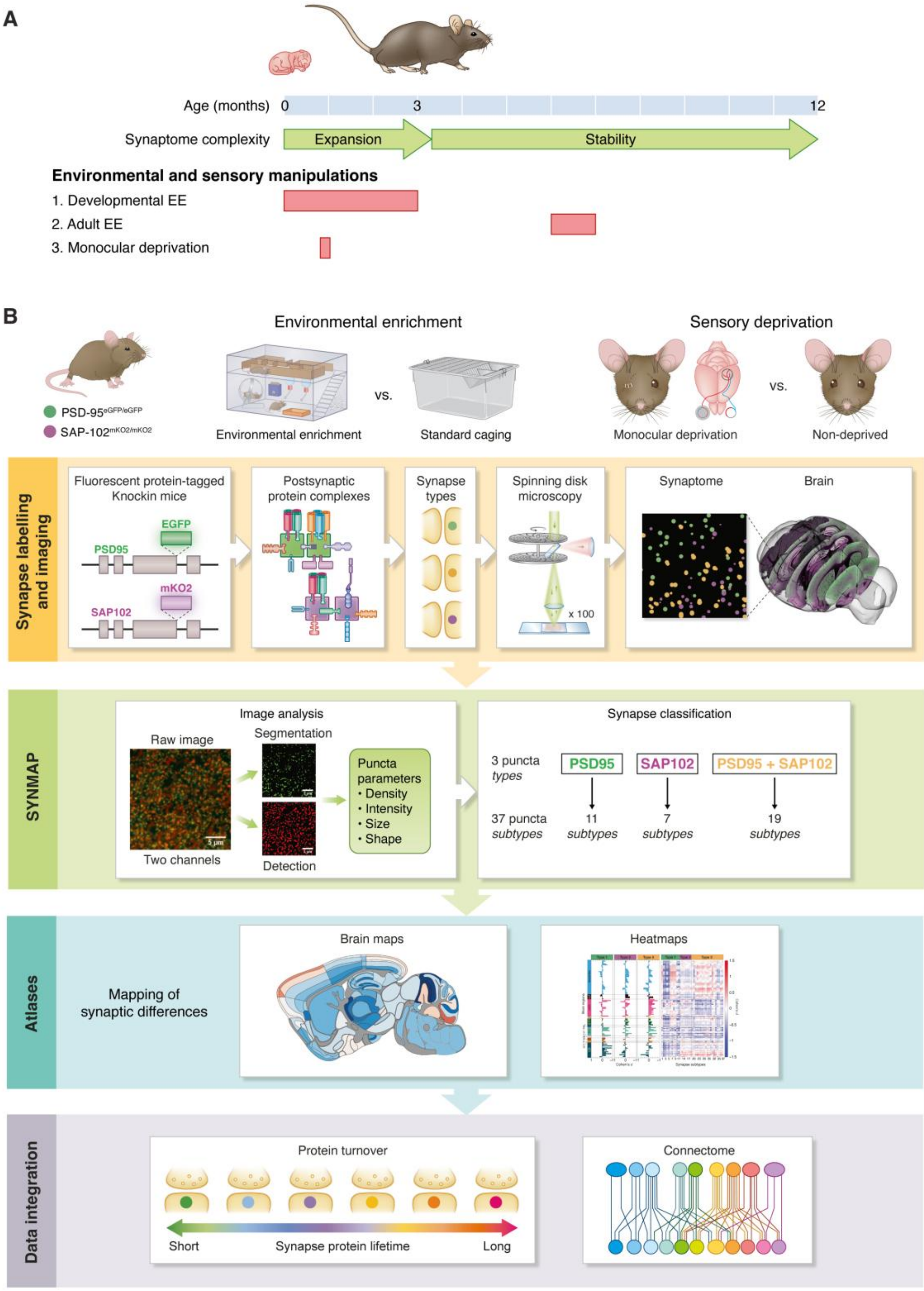

Fig. S1) A) Timeline of EE and MD paradigms aligned to shift in synaptome diversity over the lifespan. B) Schematic overview of mapping and analysis of experience-dependent change of the synaptome architecture.

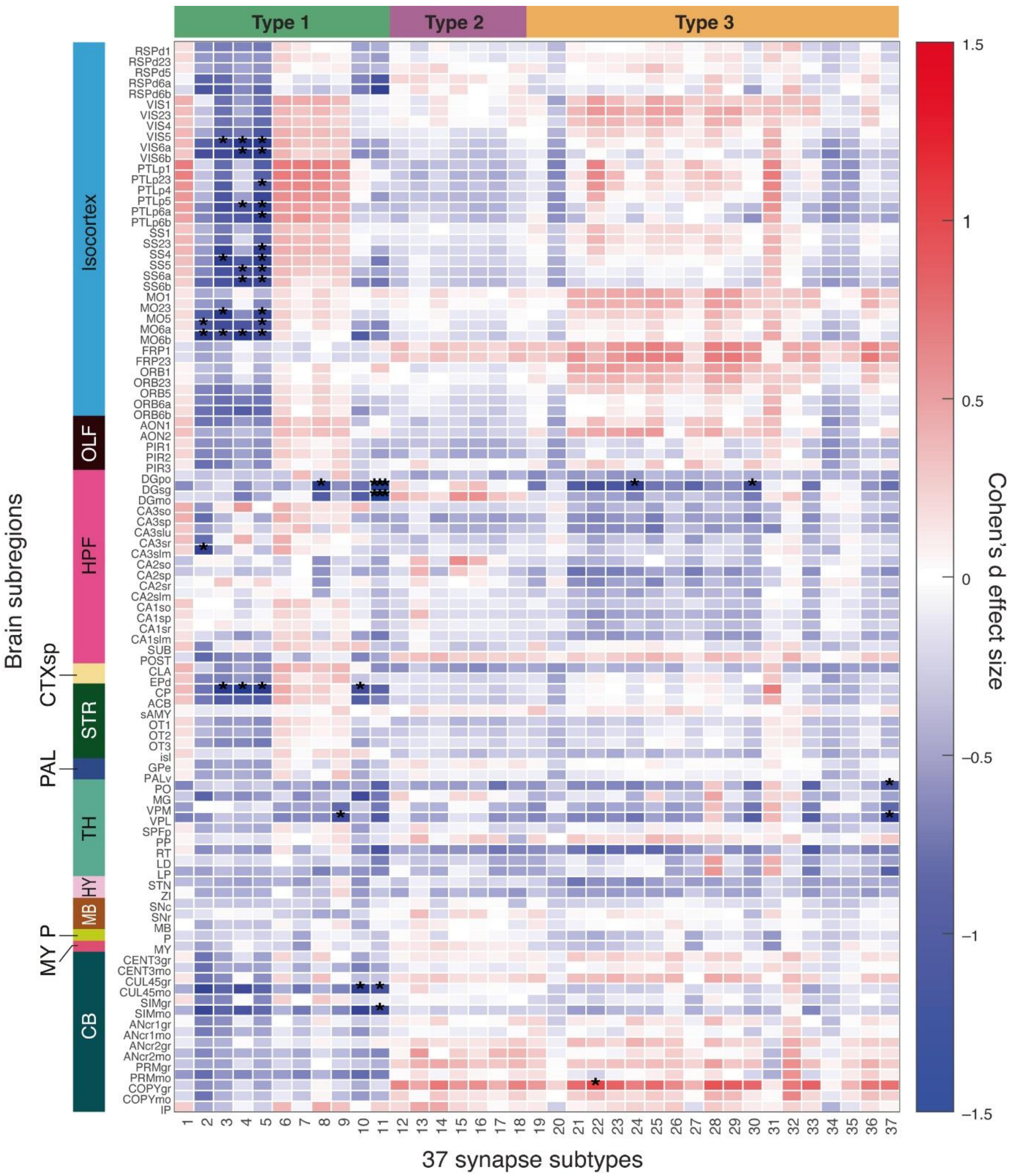

Fig. S2) Synapse subtype density differences across all brain subregions between developmental EE and SC mice (related to Fig. 1C). Heatmap shows Cohen's *d* effect size for 37 synapse subtypes; red indicates higher density in EE mice. Asterisks denote statistical significance (*p < 0.05; ***p < critical p after Benjamini–Hochberg correction applied to Bayesian posterior probabilities). Significance after correction was found for subtype 11 (p < 0.00047). For brain region abbreviations see supplementary material 'Brain region list'.

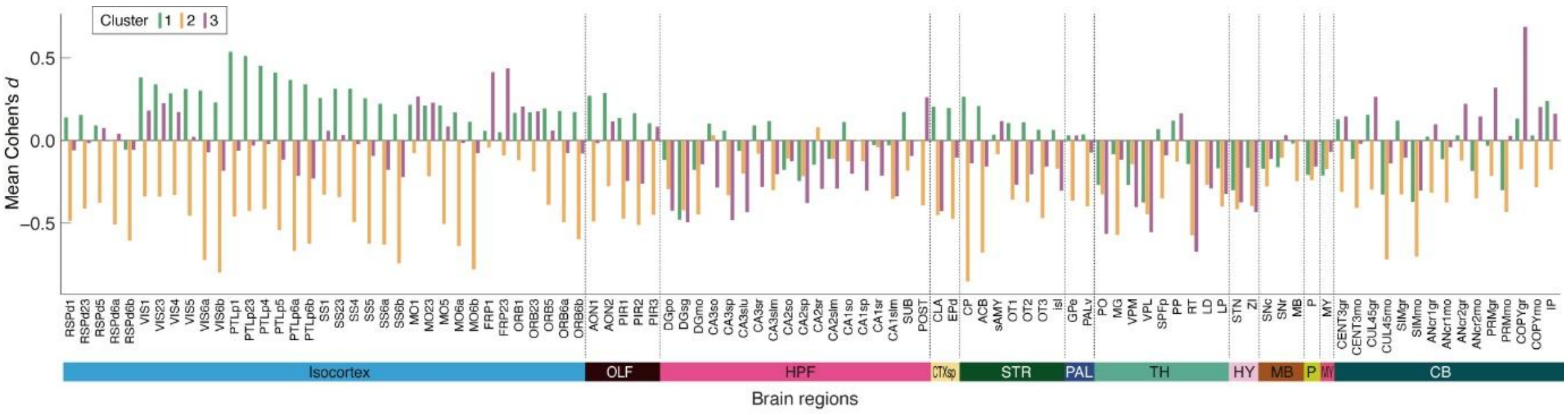

Fig. S3) Mean Cohen's *d* per brain subregion stratified by subtype cluster (see key), for developmental EE versus control comparison (related to Fig. 1E). Cluster-level regional effects were calculated by averaging Cohen's *d* values across the subtypes assigned to each PCA-derived cluster.

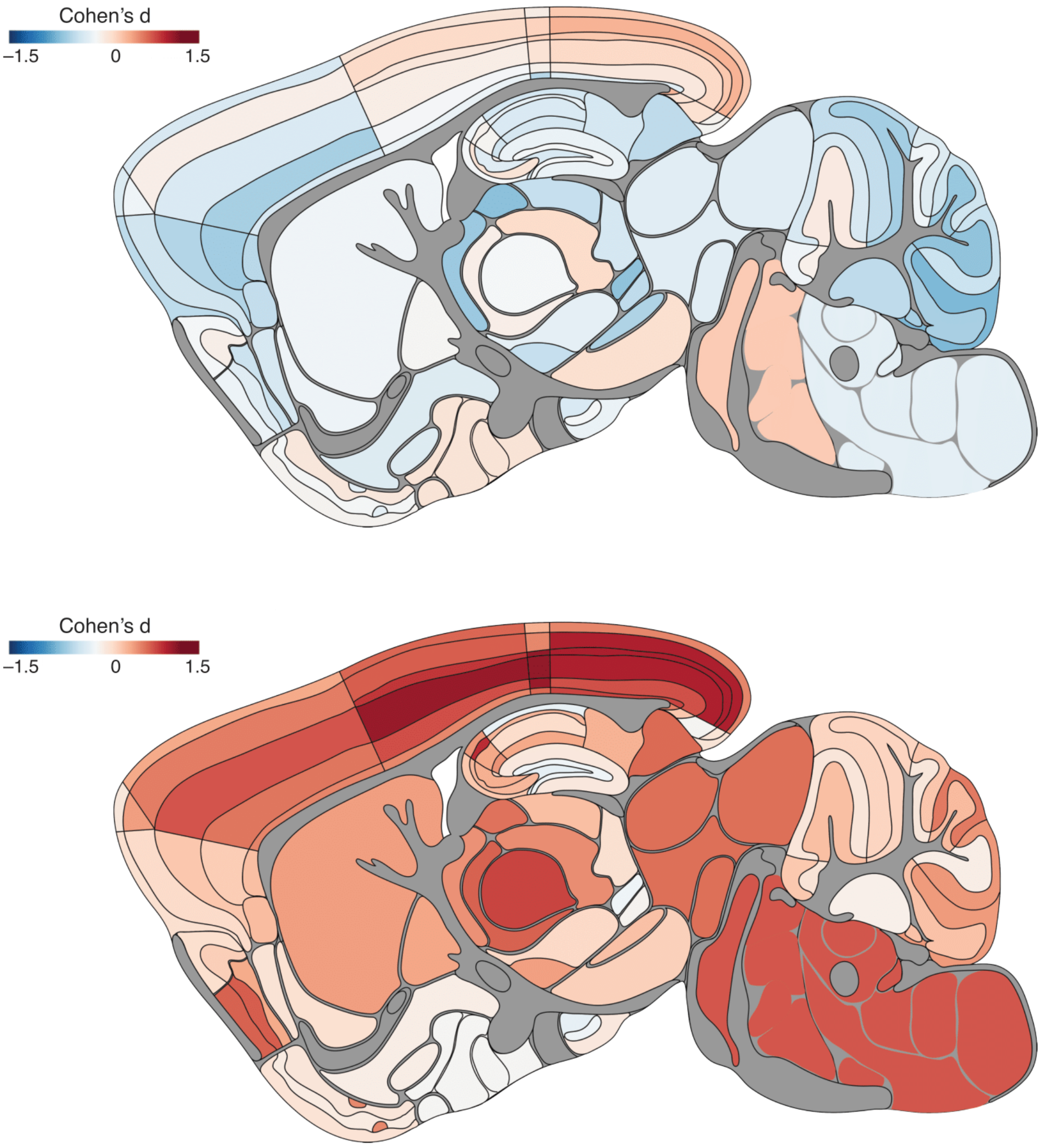

Fig. S4) Parasagittal brain maps comparing total synapse density between female and male mice in 3-month SC (top) and developmental EE (bottom) groups with Cohen's *d* effect size. Red indicates higher density in females. See Fig. S32 for region key.

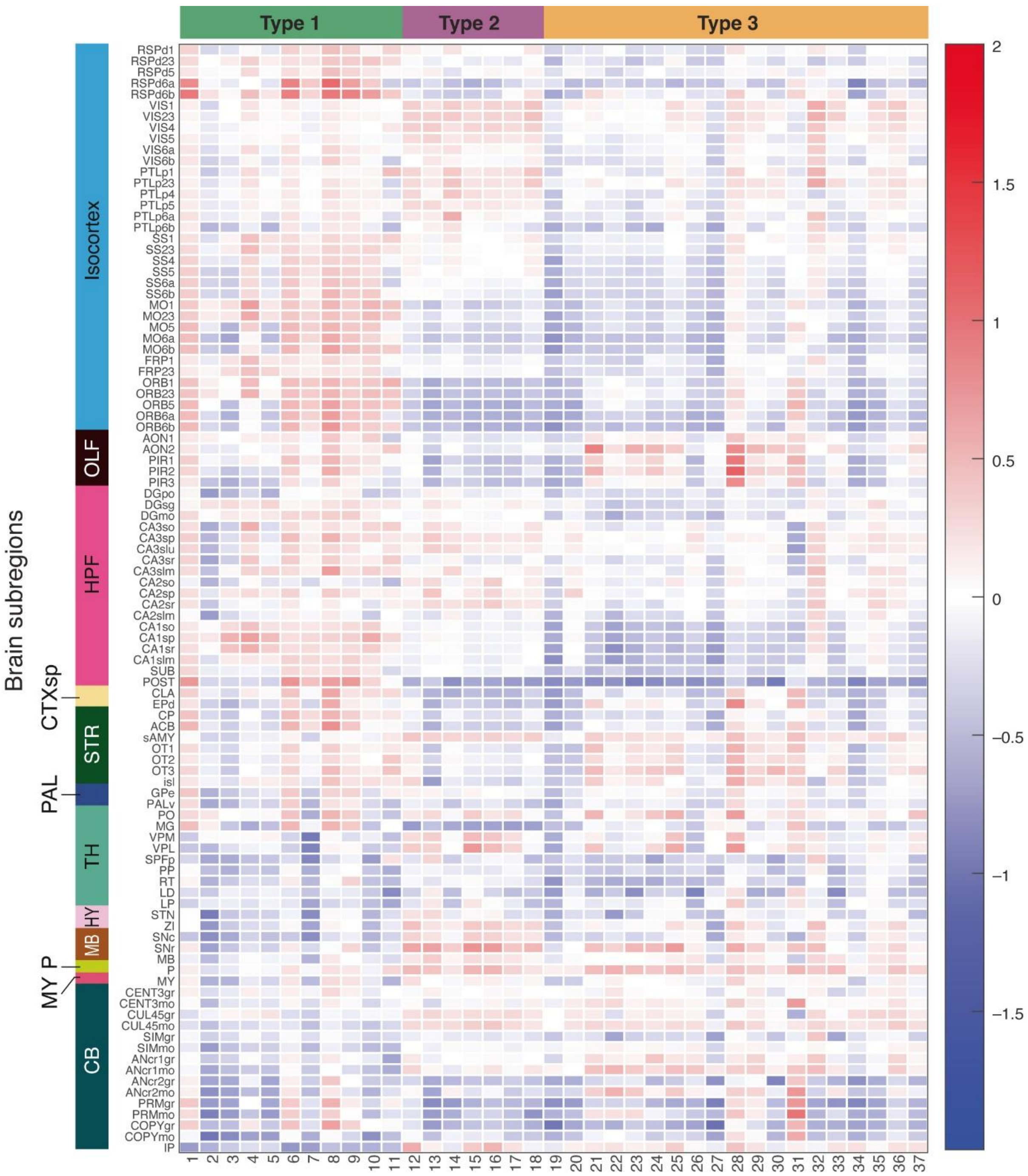

Fig. S5) Cohen's *d* effect size values comparing synapse subtype densities between 3-month SC female and male mice across all brain subregions. Red indicates higher density in females. No significance was retained after Benjamini–Hochberg correction applied to Bayesian posterior probabilities, indicating no strong sex differences in synaptome architecture under SC. For brain region abbreviations see supplementary material 'Brain region list'.

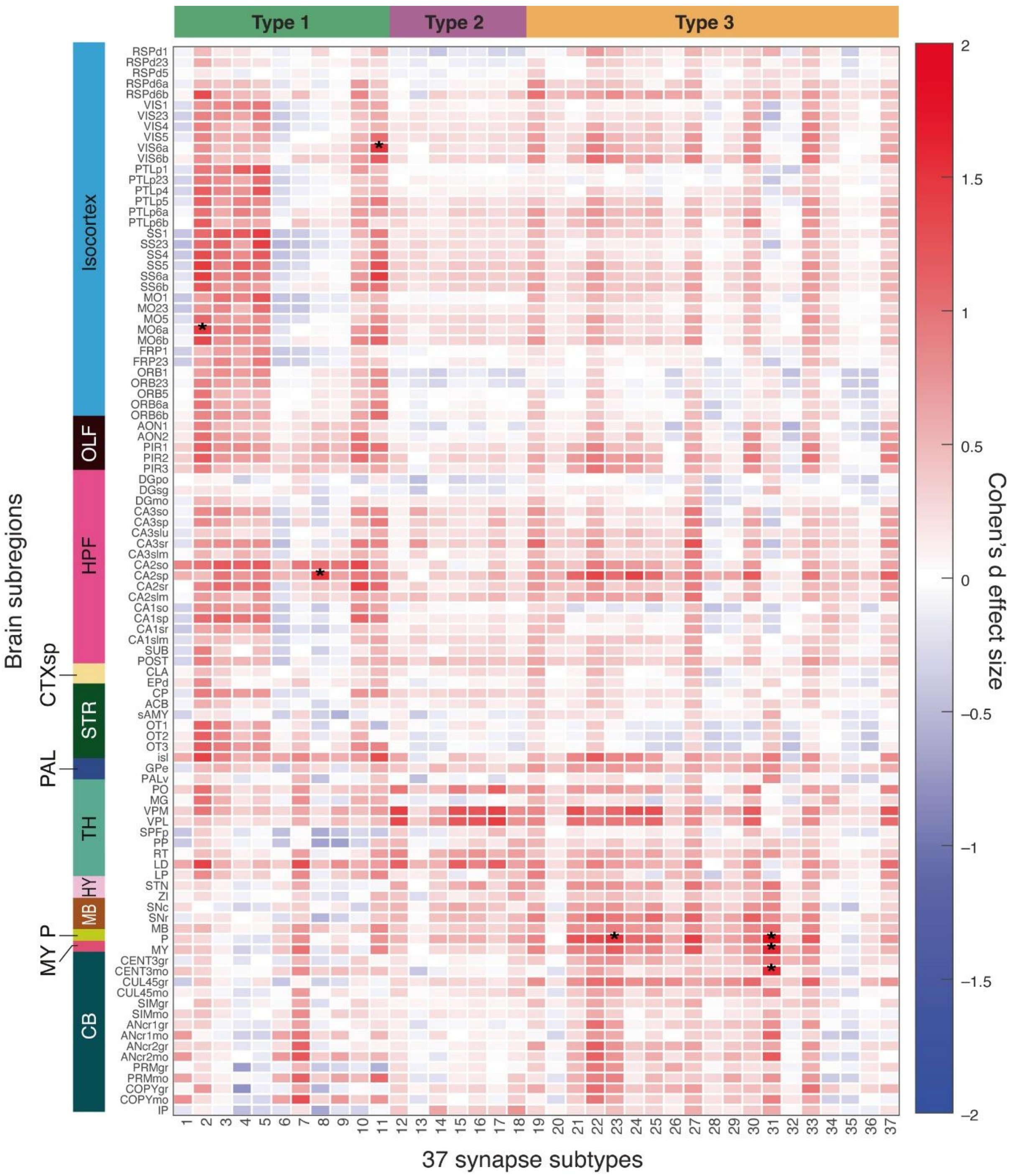

Fig. S6) Cohen's *d* effect size values comparing synapse subtype densities between developmental EE female and male mice. Red indicates higher density in females. Asterisk denotes statistical significance (*$p < 0.05$); no significance was retained after Benjamini–Hochberg correction applied to Bayesian posterior probabilities, indicating no strong sex differences in synaptome architecture under EE. However, trends indicate that developmental EE may trigger some sex-dependent changes in synaptome architecture where EE females have higher synapse densities than EE males for many subtypes. For brain region abbreviations see supplementary material 'Brain region list'.

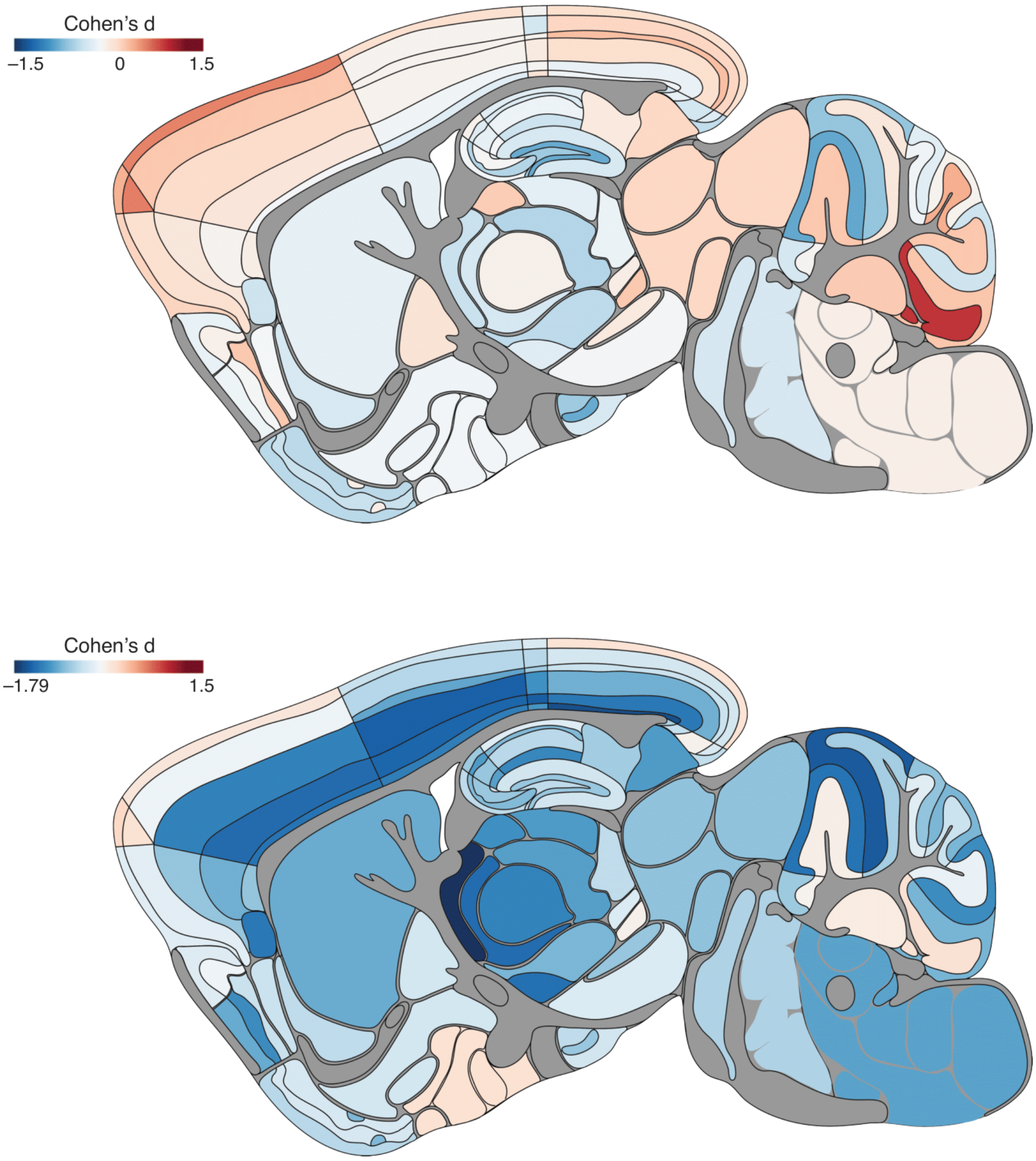

Fig. S7) Parasagittal brain maps comparing total synapse density with Cohen's *d* effect size between developmental EE and SC mice, split by sex. Top: EE vs. SC females; bottom: EE vs. SC males. Red indicates higher density in EE mice. See Fig. S32 for region key.

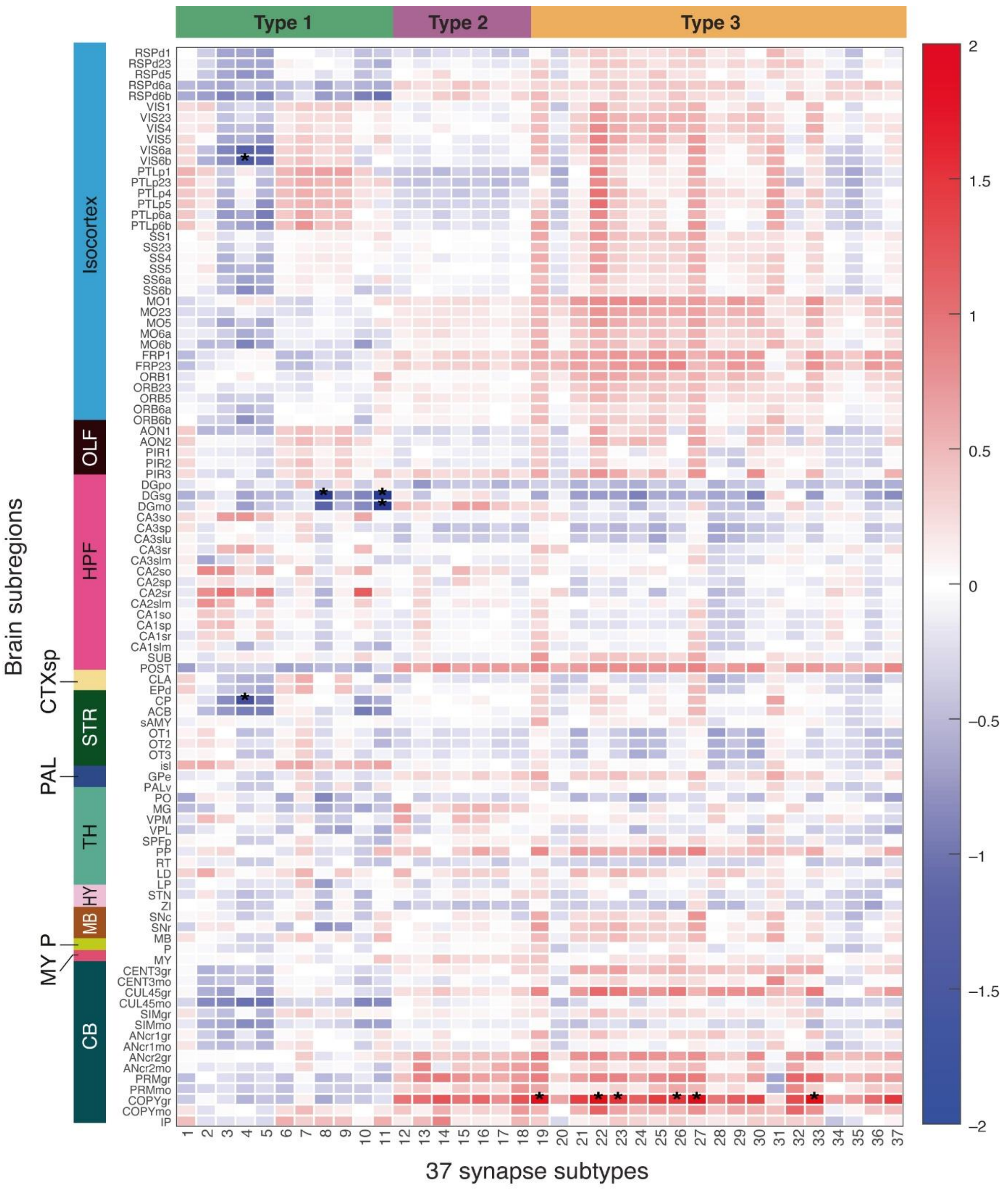

Fig. S8) Cohen's *d* effect size values comparing synapse subtype densities between developmental EE and SC females. Red indicates higher density in EE mice. Asterisks denote statistical significance (*$p < 0.05$); no significance was retained after Benjamini–Hochberg correction applied to Bayesian posterior probabilities. EE females showed non-significant trends of higher Type 3 subtypes and lower Type 1 subtypes in the isocortex and cerebellum compared to SC females. For brain region abbreviations see supplementary material 'Brain region list'.

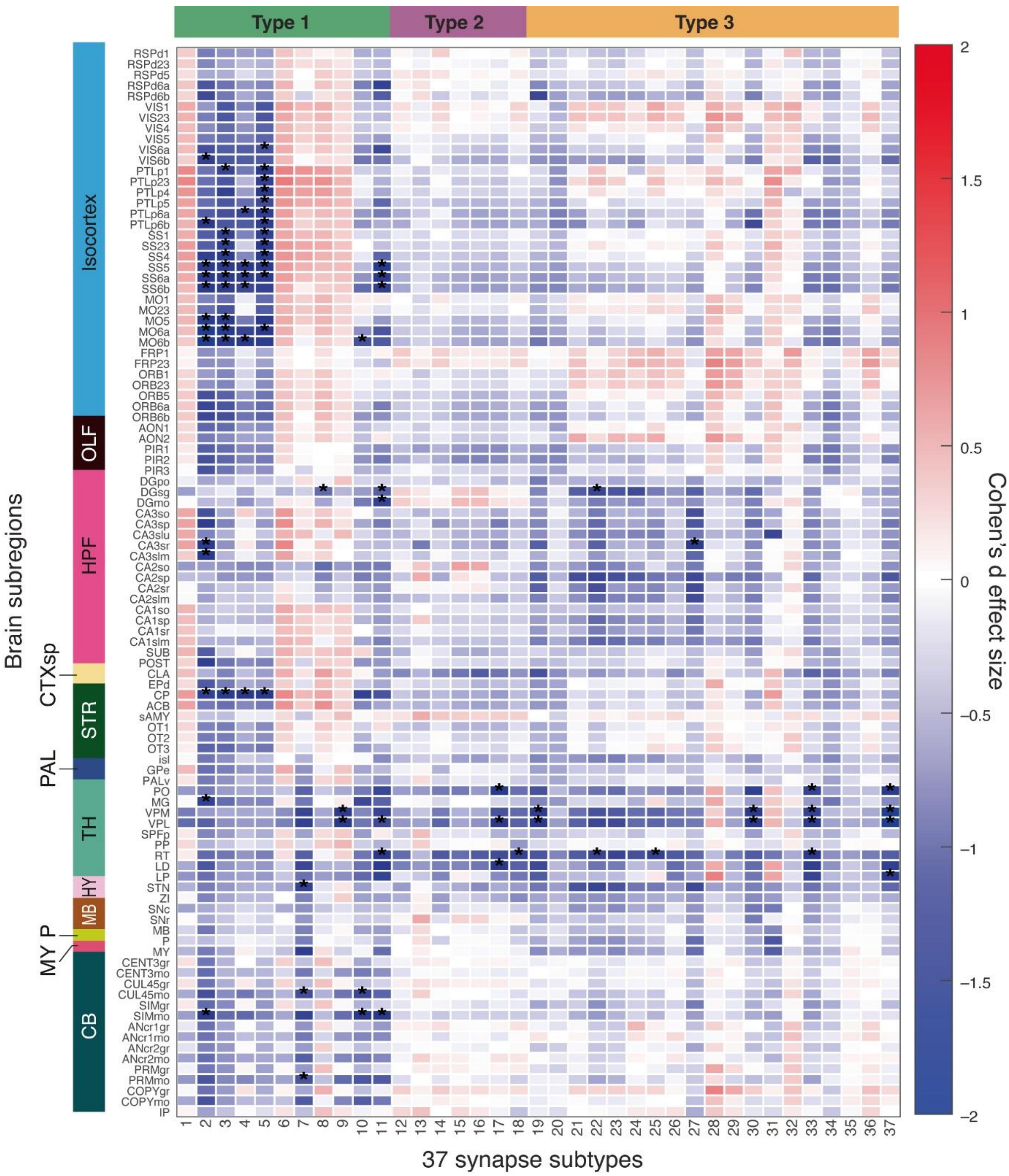

Fig. S9) Cohen's *d* effect size values comparing synapse subtype densities between developmental EE and SC males. Red indicates higher density in EE mice. Asterisks denote statistical significance (*$p < 0.05$); no significance was retained after Benjamini–Hochberg correction applied to Bayesian posterior probabilities. EE males exhibited widespread reduction in a subset of Type 1 subtypes, with additional decreases in Type 3 subtypes in hippocampus and thalamus. For brain region abbreviations see supplementary material 'Brain region list'.

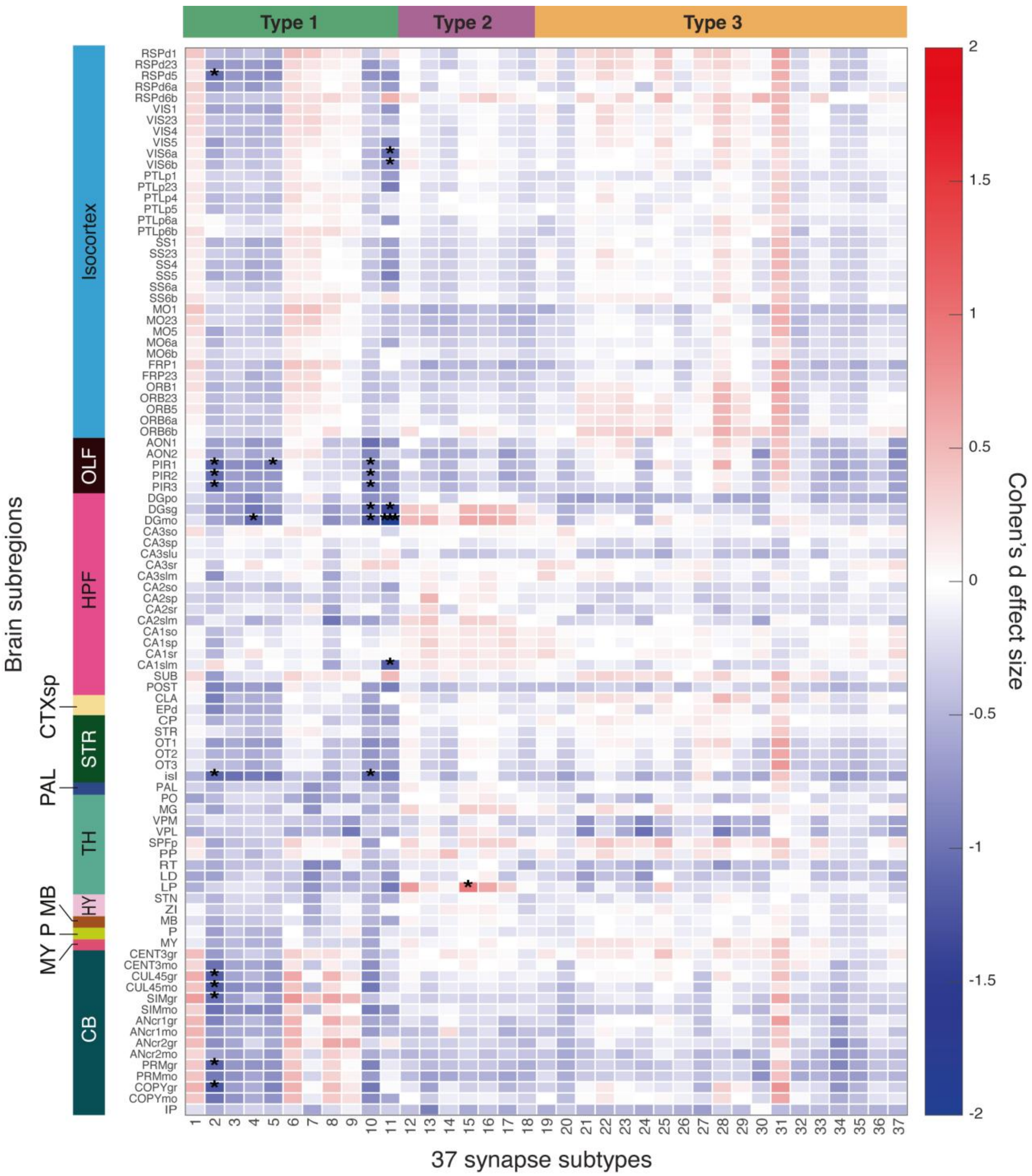

Fig. S10) Cohen's *d* effect size values comparing synapse subtype densities between adult EE and SC mice (related to Fig. 1G). Red indicates higher density in EE mice. Asterisks denote statistical significance (*p < 0.05); ***p < critical p after Benjamini-Hochberg correction applied to Bayesian posterior probabilities. Subtype11 showed significance after correction (p < 0.0004). For brain region abbreviations see supplementary material 'Brain region list'.

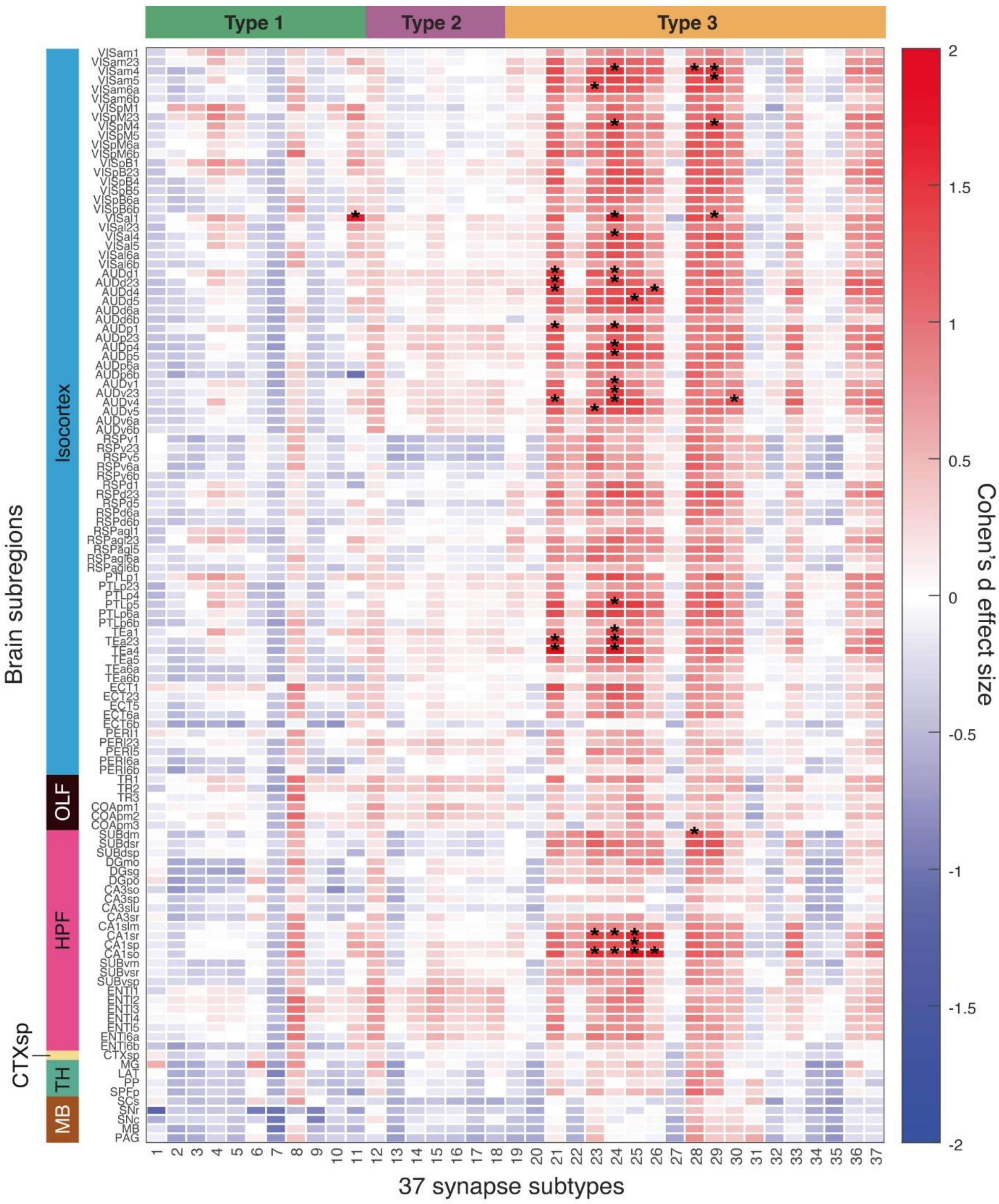

Fig. S11) Cohen's *d* effect size values comparing synapse subtype densities across the contralateral hemisphere between MD and control mice (related to Fig. 2C). Red indicates higher density in MD mice. Asterisk denotes statistical significance (*$p < 0.05$); no significance was retained after Benjamini–Hochberg correction applied to Bayesian posterior probabilities. For brain region abbreviations see supplementary material 'Brain region list'.

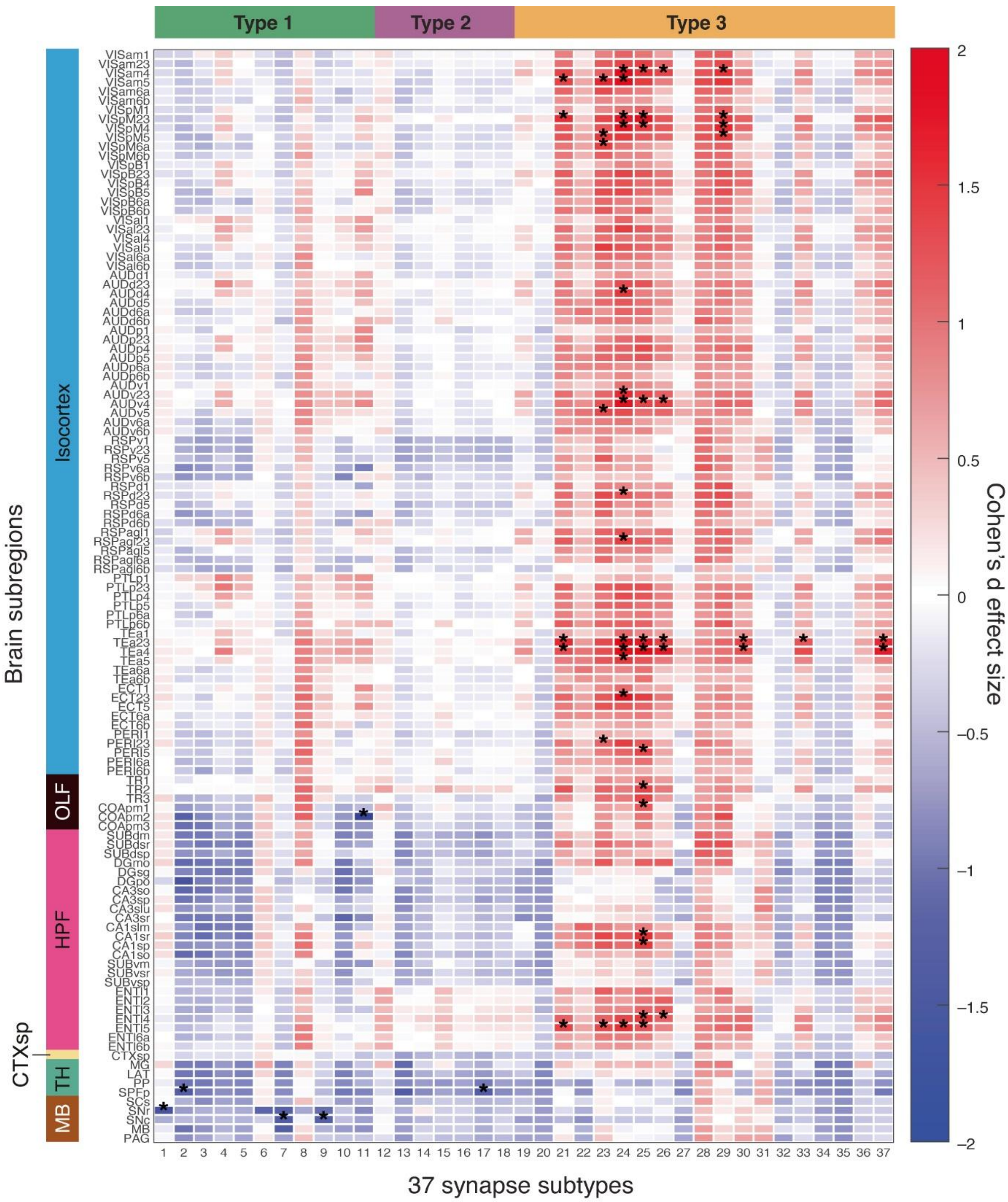

Fig. S12) Cohen's *d* effect size values comparing synapse subtype densities across the ipsilateral hemisphere between MD and control mice. Red indicates higher density in MD mice. Asterisk denotes statistical significance (*p < 0.05); no significance was retained after Benjamini–Hochberg correction applied to Bayesian posterior probabilities. A trend toward lower Type 1 and Type 2 subtype densities was observed in the hippocampus, thalamus, and midbrain—distinct from the contralateral hemisphere. For brain region abbreviations see supplementary material 'Brain region list'.

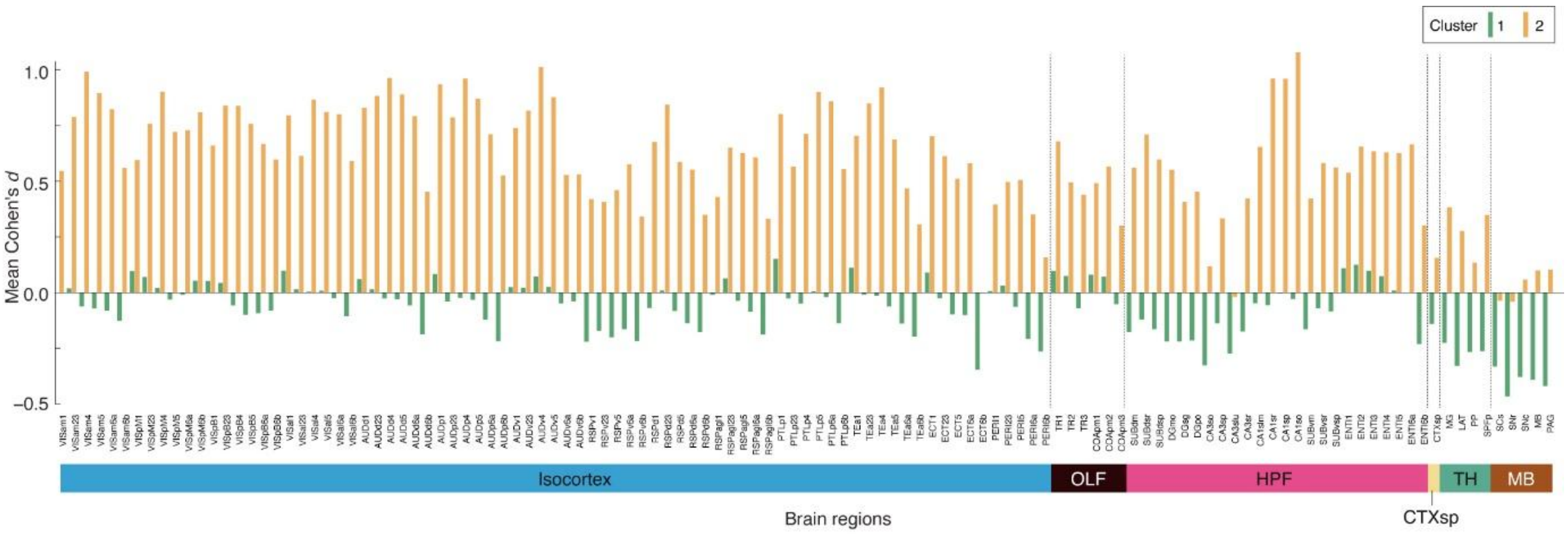

Fig. S13) Mean Cohen's *d* per brain subregion stratified by subtype cluster (see key), for MD versus control comparison (related to Fig. 2F). Cluster-level regional effects were calculated by averaging Cohen's *d* values across the subtypes assigned to each PCA-derived cluster.

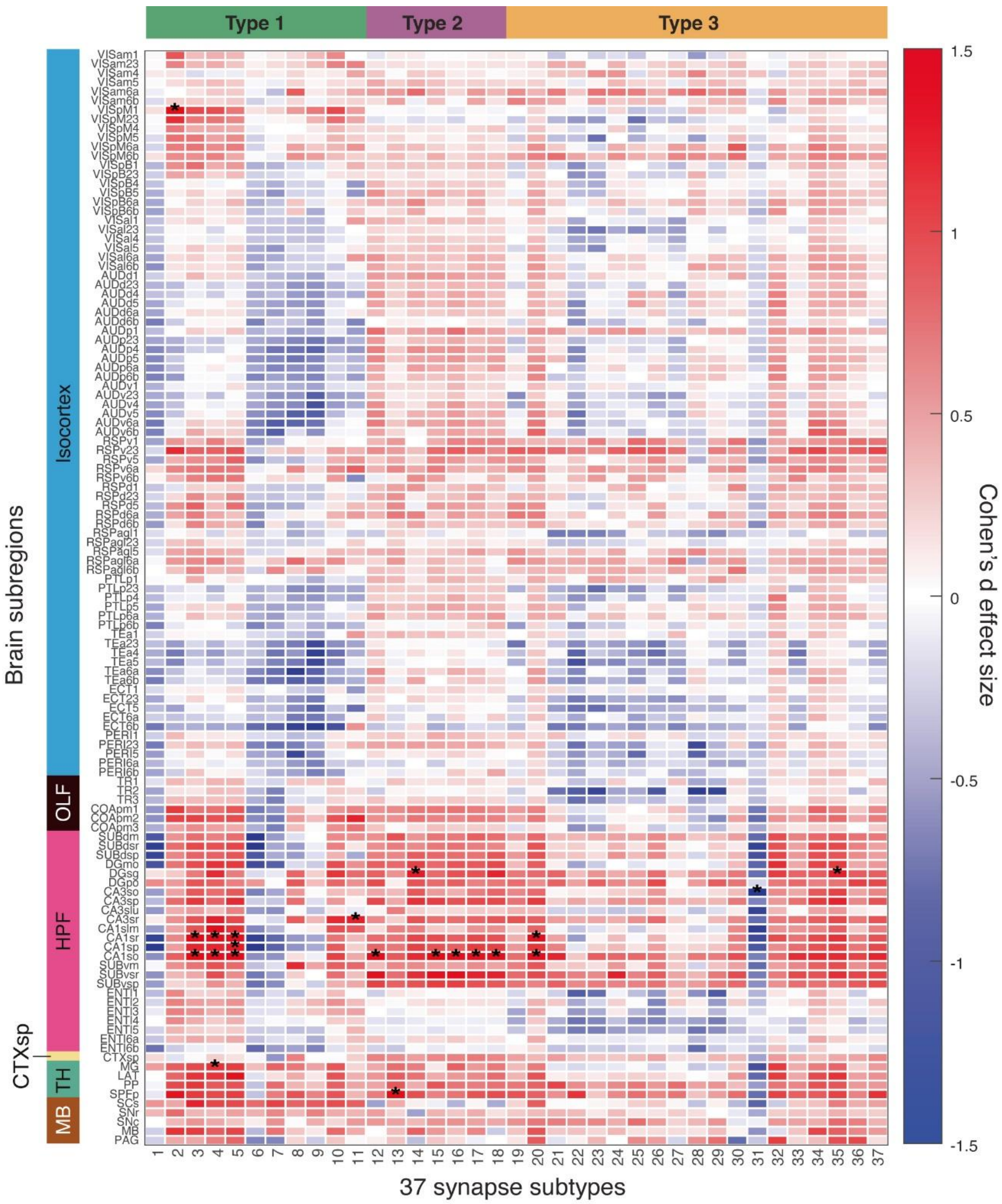

Fig. S14) Cohen's *d* effect size values comparing synapse subtype densities across the contralateral hemisphere, normalised by summed hemisphere values, between MD and control mice. Red indicates subtypes that were relatively more abundant in the contralateral hemisphere of MD mice than in controls. Asterisk denotes statistical significance (*$p < 0.05$); no significance was retained after Benjamini–Hochberg correction applied to Bayesian posterior probabilities. A subset of subtypes—unrestricted by protein class—were proportionally higher in the visual cortex, retrosplenial cortex, hippocampus, thalamus, and midbrain of the contralateral hemisphere of MD mice. For brain region abbreviations see supplementary material 'Brain region list'.

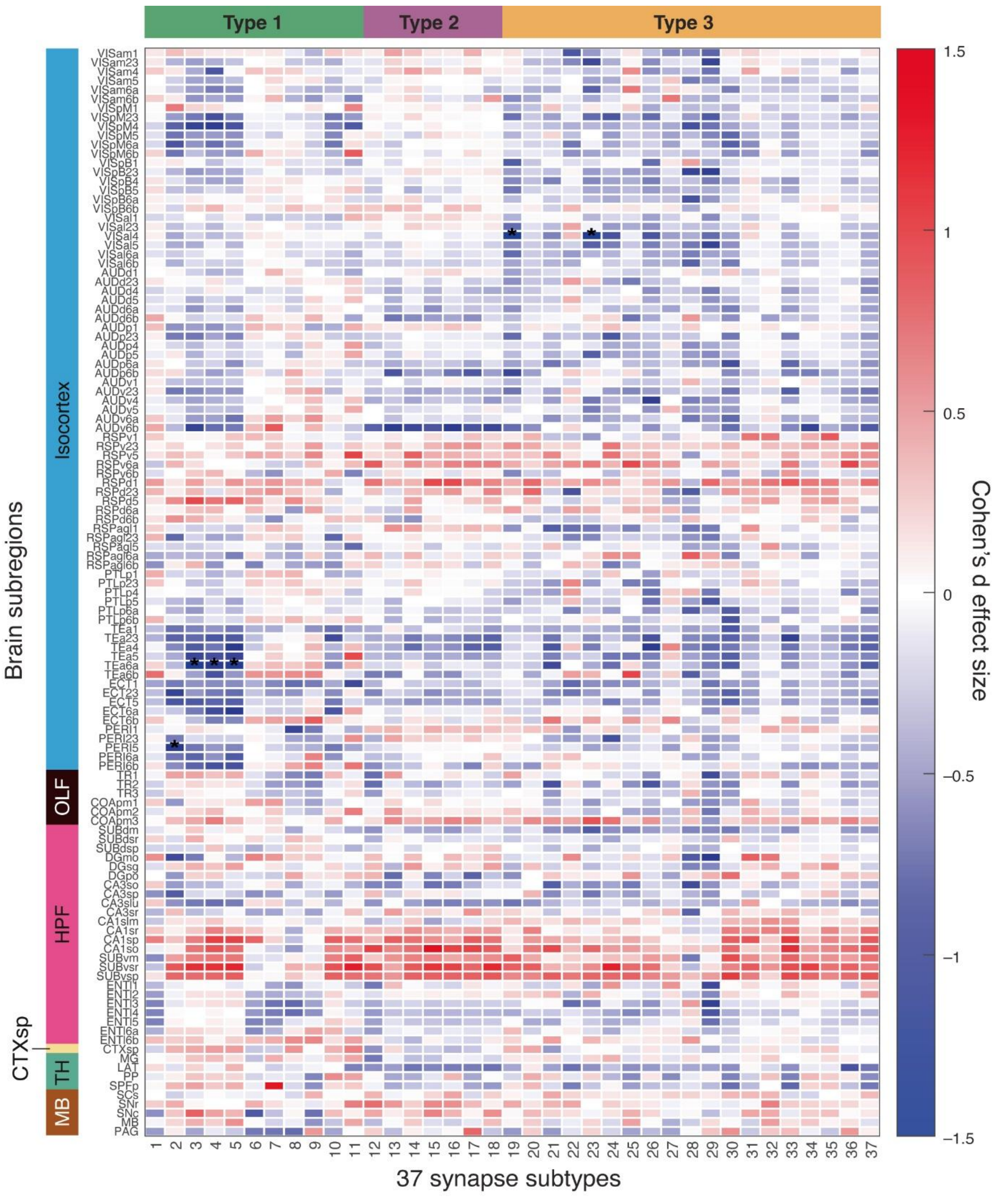

Fig. S15) Cohen's *d* effect size values comparing inter-hemispheric differences in subtype densities, normalised by summed hemisphere values, between MD and control mice. Red indicates greater asymmetry in MD mice than in controls. Asterisk denotes statistical significance (*p < 0.05); no significance was retained after Benjamini–Hochberg correction applied to Bayesian posterior probabilities. MD-induced shifts in the two hemispheres reduced asymmetry in many isocortical regions, while increasing asymmetry in the CA1 and subiculum. For brain region abbreviations see supplementary material 'Brain region list'.

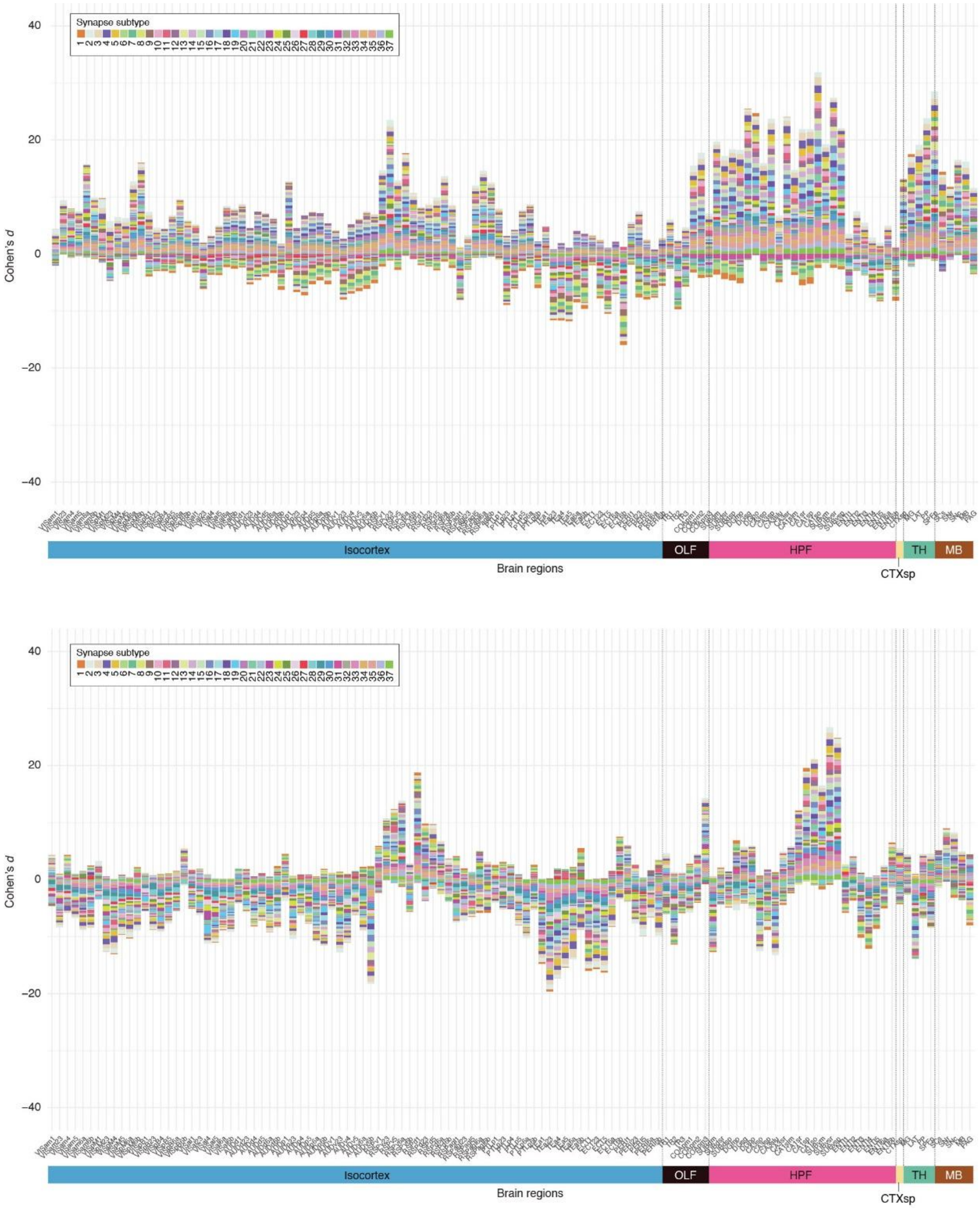

Fig. S16) Top: Stacked bar graph of Cohen's *d* values for 37 synapse subtypes in the contralateral (deprived) hemisphere, comparing MD and control mice (related to Fig. 2G). Subregion values were normalized by dividing contralateral densities by the sum of ipsilateral and contralateral densities. Positive values indicate increased relative contralateral density in MD. **Bottom:** Stacked bar graph of Cohen's *d* values for 37 synapse subtypes comparing interhemispheric differences between MD and control mice. The interhemispheric difference for each subtype and subregion was calculated by dividing the absolute difference between contralateral and ipsilateral densities by the sum of ipsilateral and contralateral densities. Positive values indicate increased interhemispheric difference with MD. For brain region abbreviations see supplementary material 'Brain region list'.

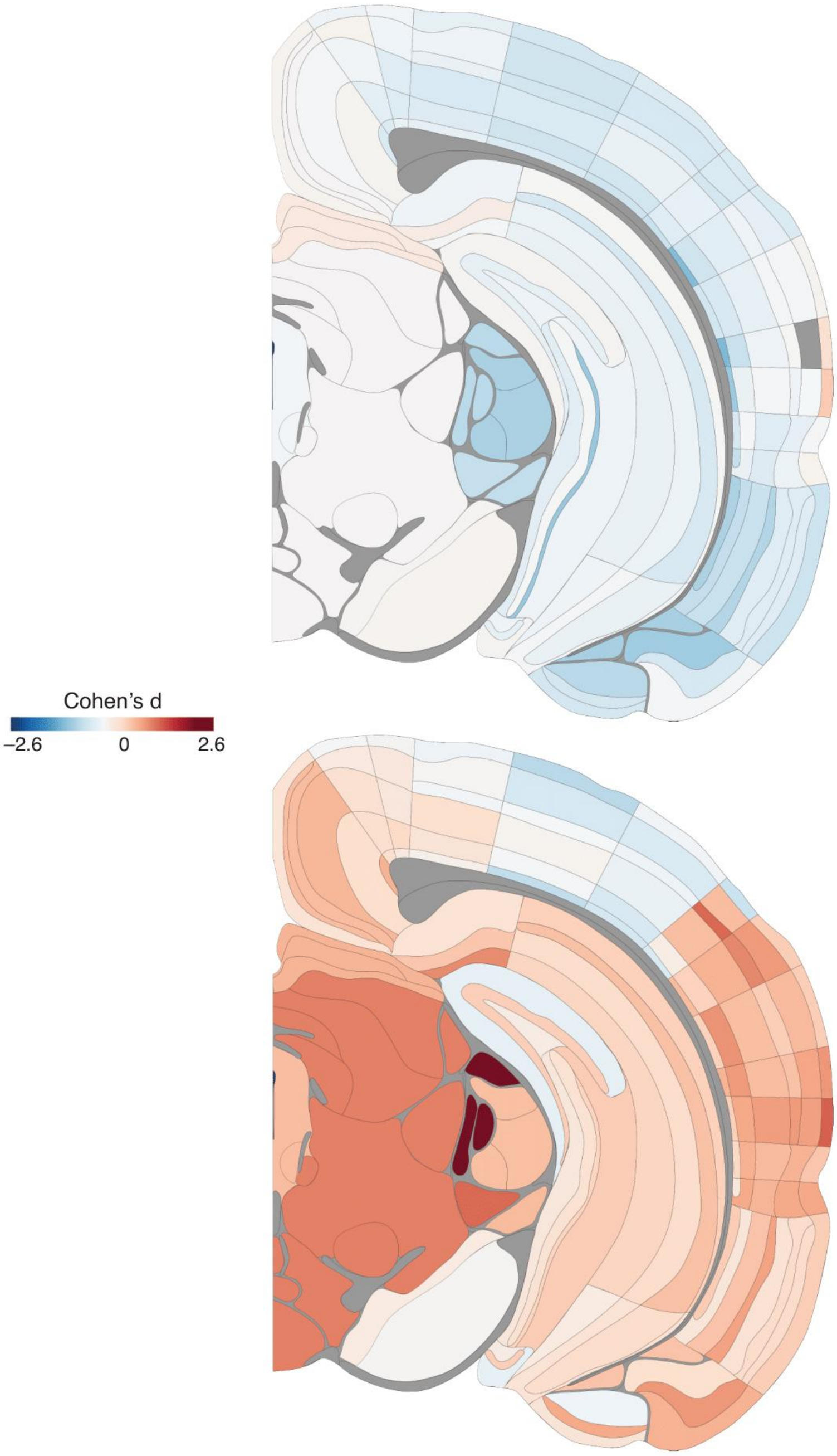

Fig. S17) Coronal brain maps comparing total synapse density in the contralateral hemisphere with Cohen's *d* effect size between control females and males (top), and MD females and males (bottom). Red indicates higher density in females. See Fig. S31 for region key.

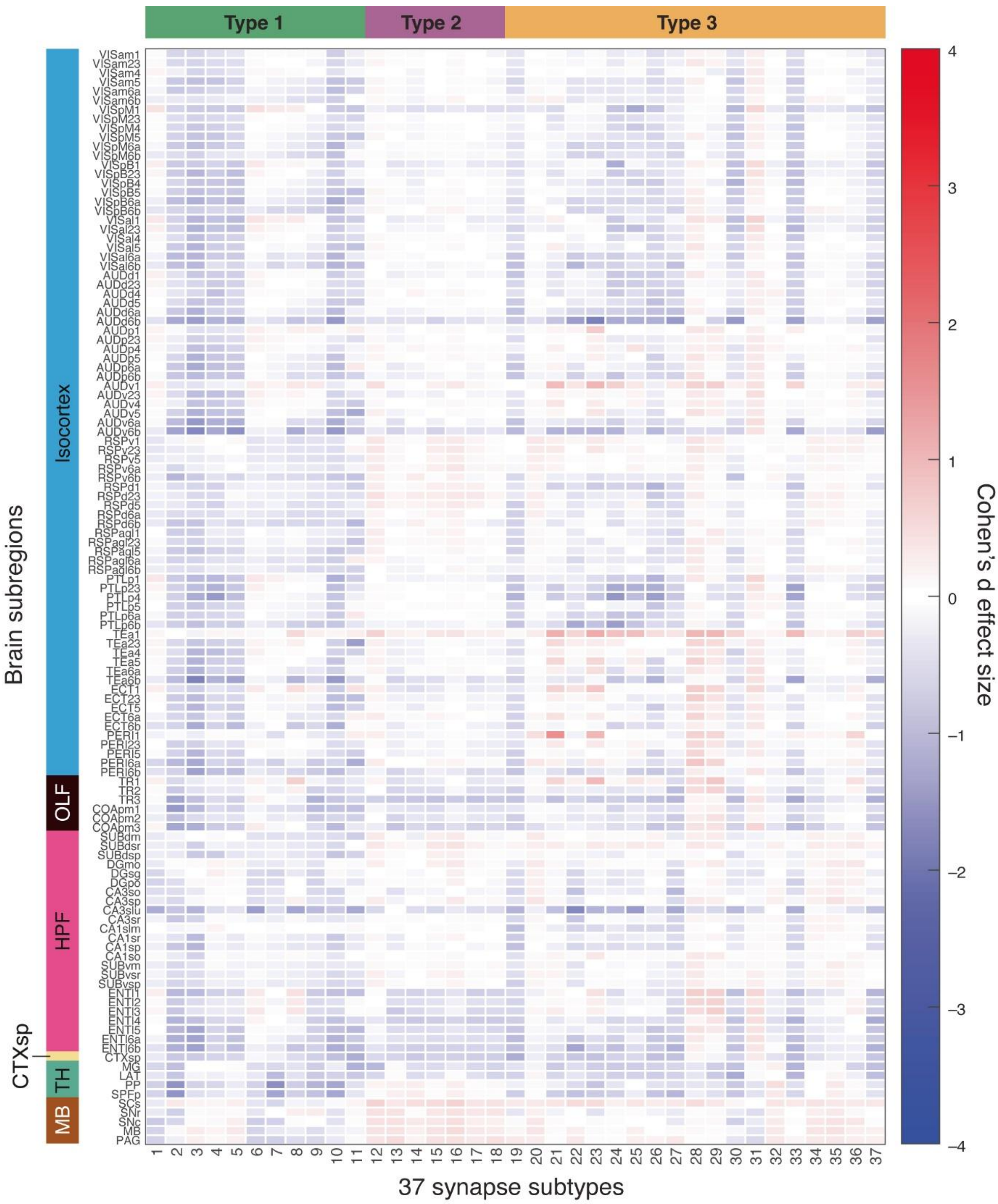

Fig. S18) Cohen's *d* effect size values comparing synapse subtype densities across the contralateral hemisphere between control females and males. Red indicates higher density in females. No significance before or after correction. A trend of lower Type 1 subtypes in the isocortex of female controls was observed. For brain region abbreviations see supplementary material 'Brain region list'.

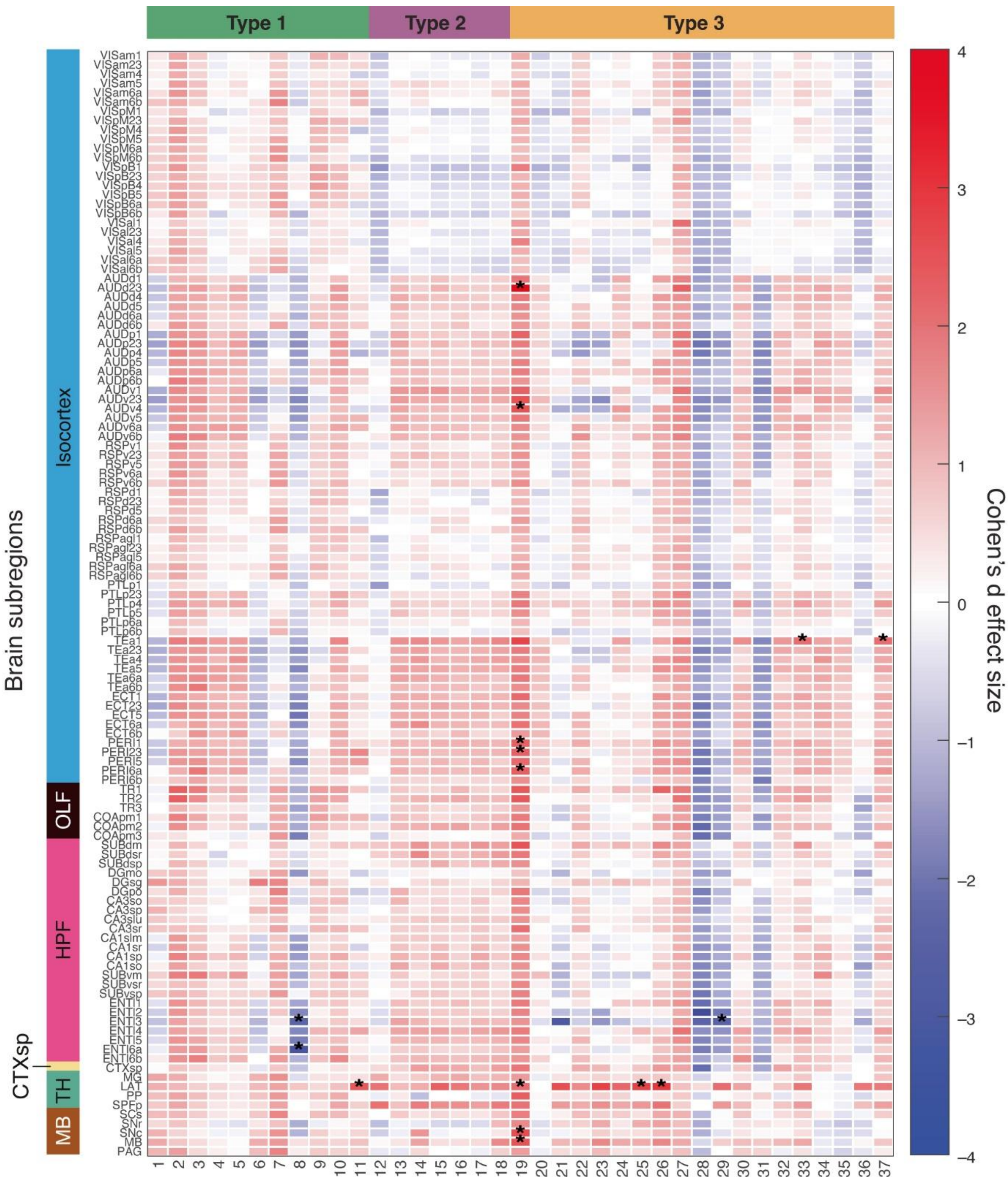

Fig. S19) Cohen's *d* effect size values comparing synapse subtype densities across the contralateral hemisphere between MD females and males. Red indicates higher density in females. Asterisk denotes statistical significance (*p < 0.05); no significance was retained after Benjamini–Hochberg correction applied to Bayesian posterior probabilities. Subtypes higher in females were not restricted to specific synapse types. As in EE, MD appears to introduce sex-dependent synaptome shifts. For brain region abbreviations see supplementary material 'Brain region list'.

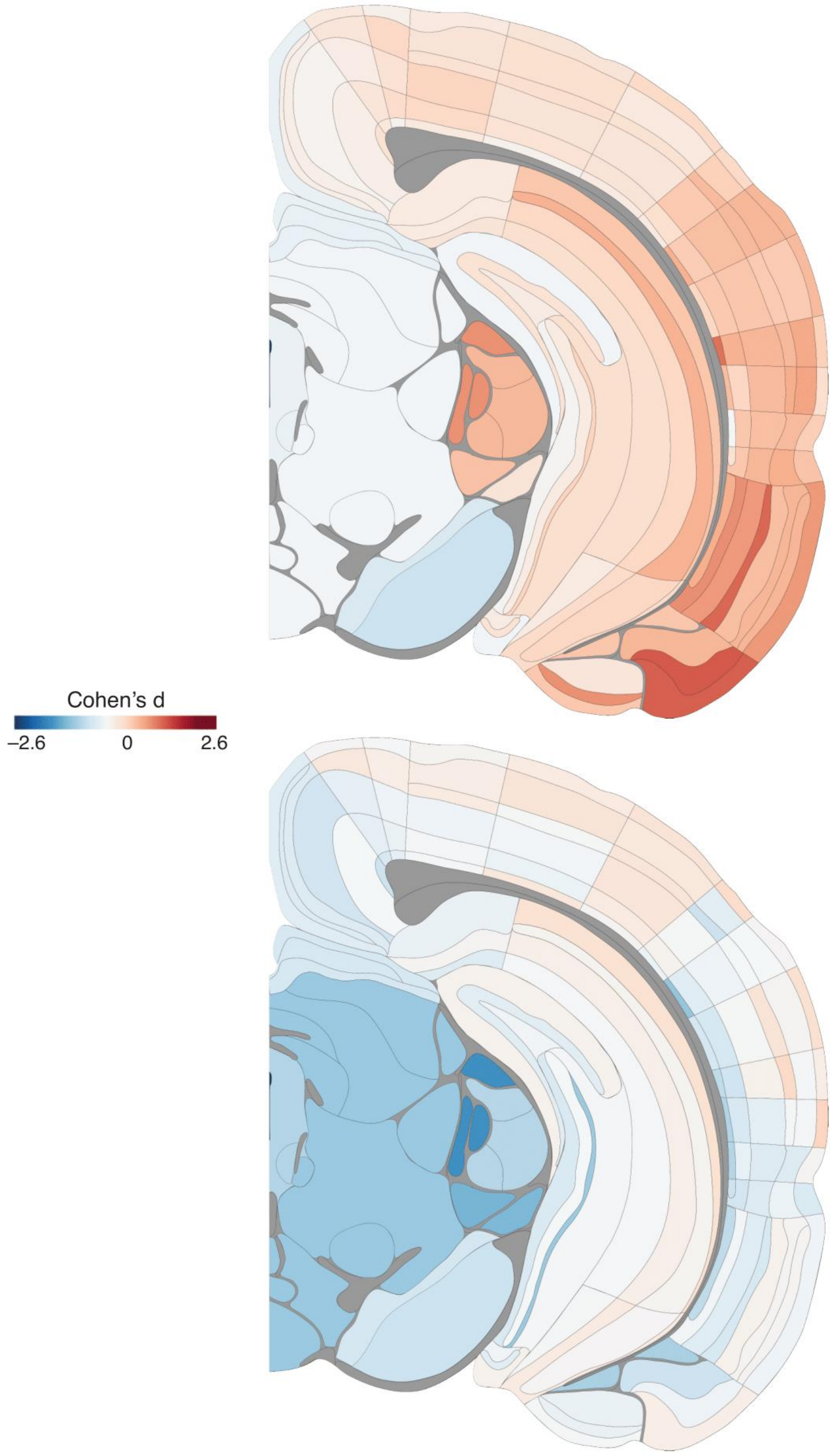

Fig. S20) Coronal brain maps comparing total synapse density with Cohen's *d* effect size between MD and control mice, split by sex. Top: MD vs. control females. Bottom: MD vs. control males. Red indicates higher density in MD mice. See Fig. S31 for region key.

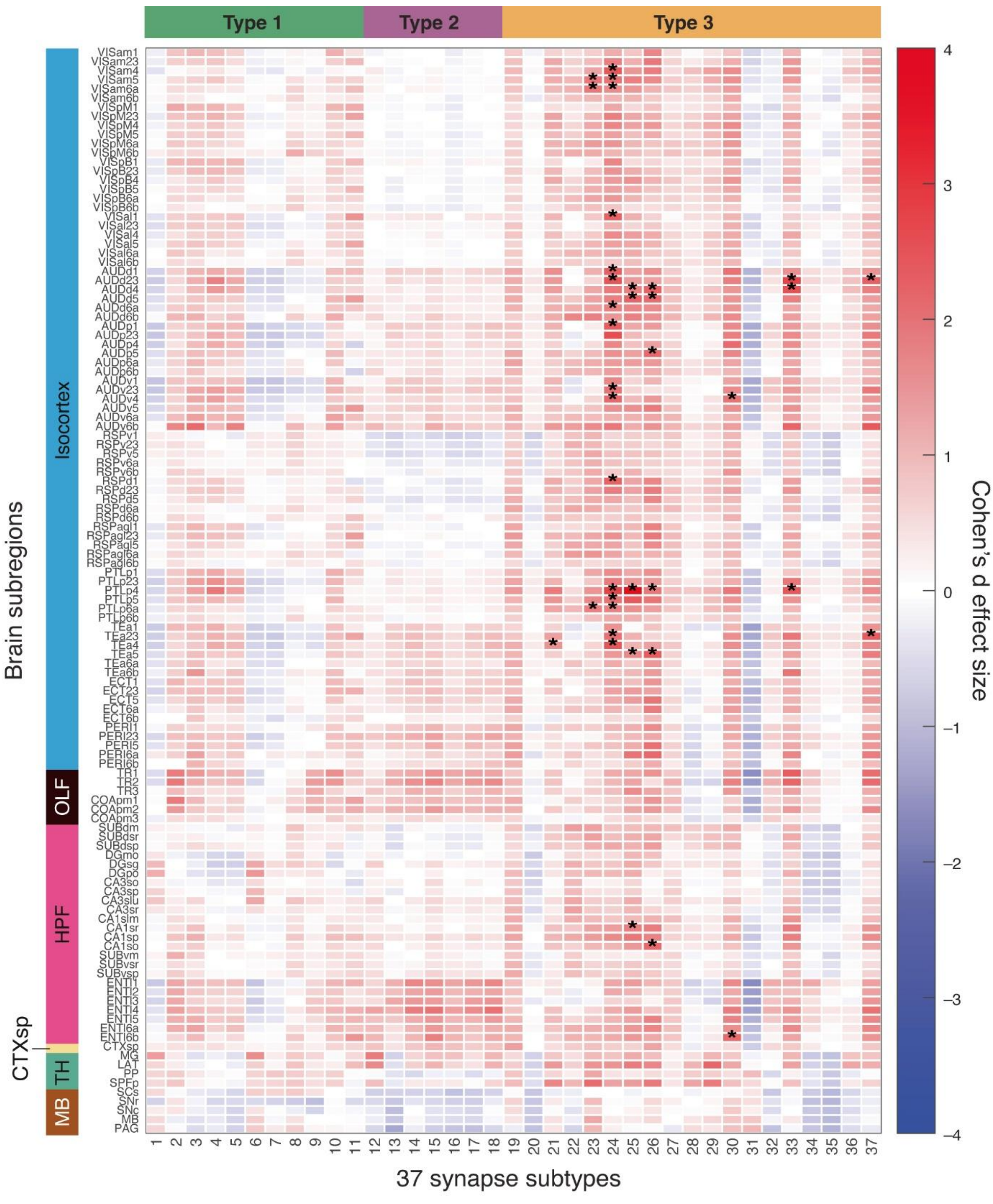

Fig. S21) Cohen's *d* effect size values comparing synapse subtype densities across the contralateral hemisphere between MD and control females. Red indicates higher density in MD mice. Asterisk denotes statistical significance (*p < 0.05); no significance was retained after Benjamini–Hochberg correction applied to Bayesian posterior probabilities. A subset of subtypes showed elevated density in MD females, independent of synapse type. For brain region abbreviations see supplementary material 'Brain region list'.

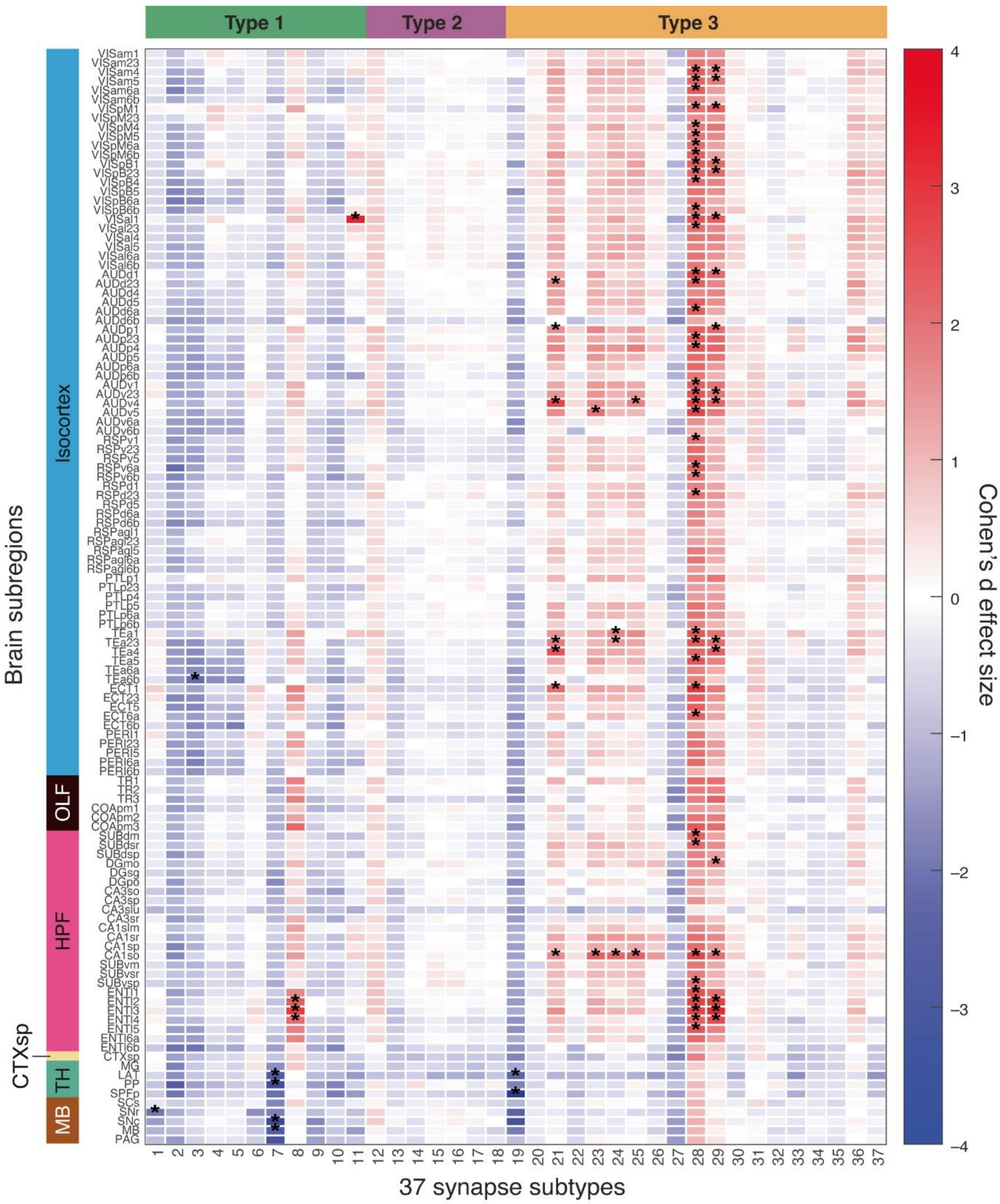

Fig. S22) Cohen's *d* effect size values comparing synapse subtype densities across the contralateral hemisphere between MD and control males. Red indicates higher density in MD mice. Asterisk denotes statistical significance (*p < 0.05); no significance was retained after Benjamini–Hochberg correction applied to Bayesian posterior probabilities. Subtypes 28 and 29 showed higher density in the cortex of MD males. MD males showed reduced density of several Type 1 subtypes across the brain, alongside significant increases in select Type 3 subtypes. For brain region abbreviations see supplementary material 'Brain region list'.

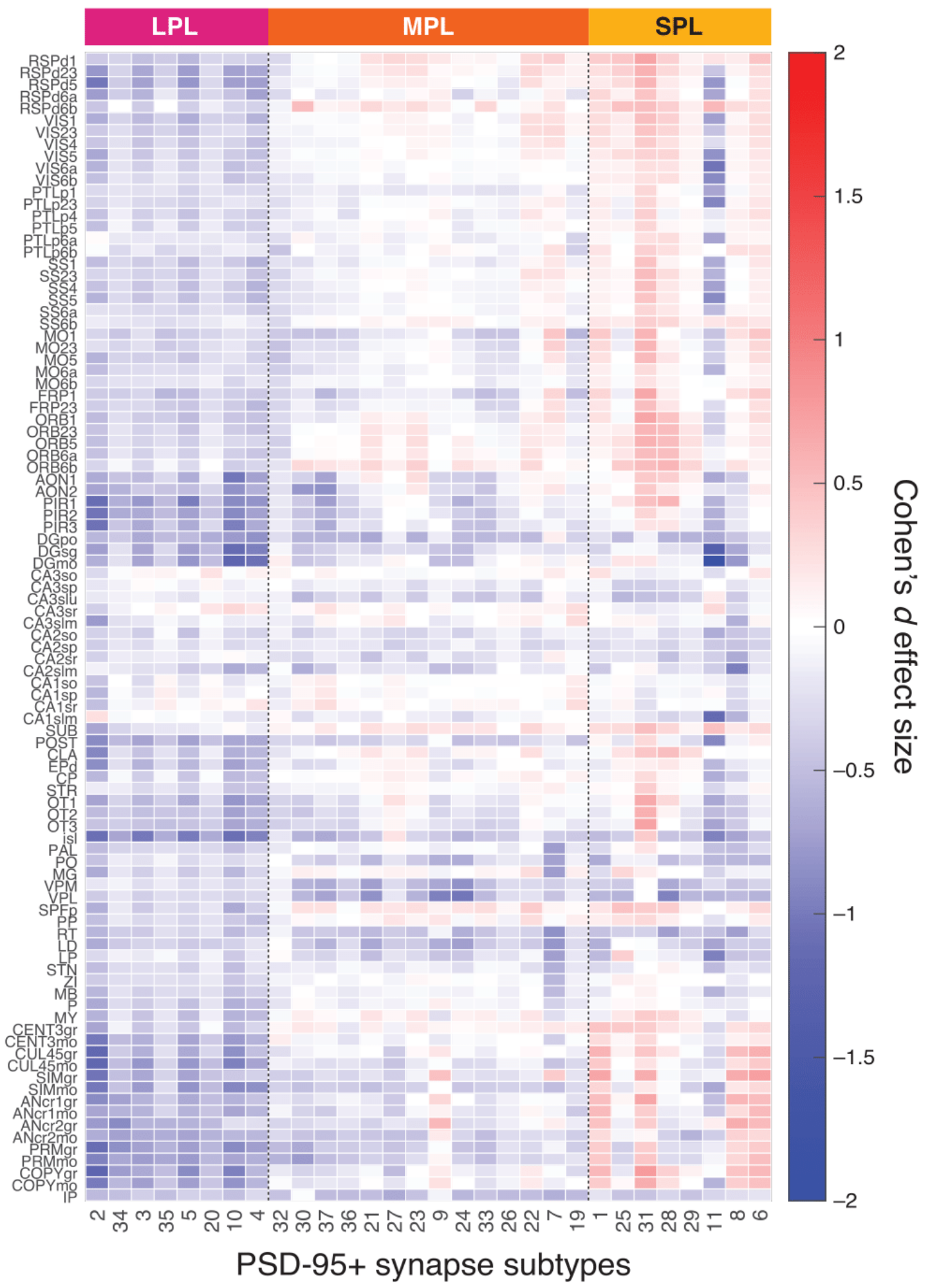

Fig. S23) Cohen's *d* effect size heatmaps showing EE-driven shifts in synapse subtypes categorised as long-protein lifetime (LPL) with adult EE paradigm (related to Fig. 3A). Similar to developmental EE, adult EE is associated with a preferential reduction in LPL synapses. For brain region abbreviations see supplementary material 'Brain region list'.

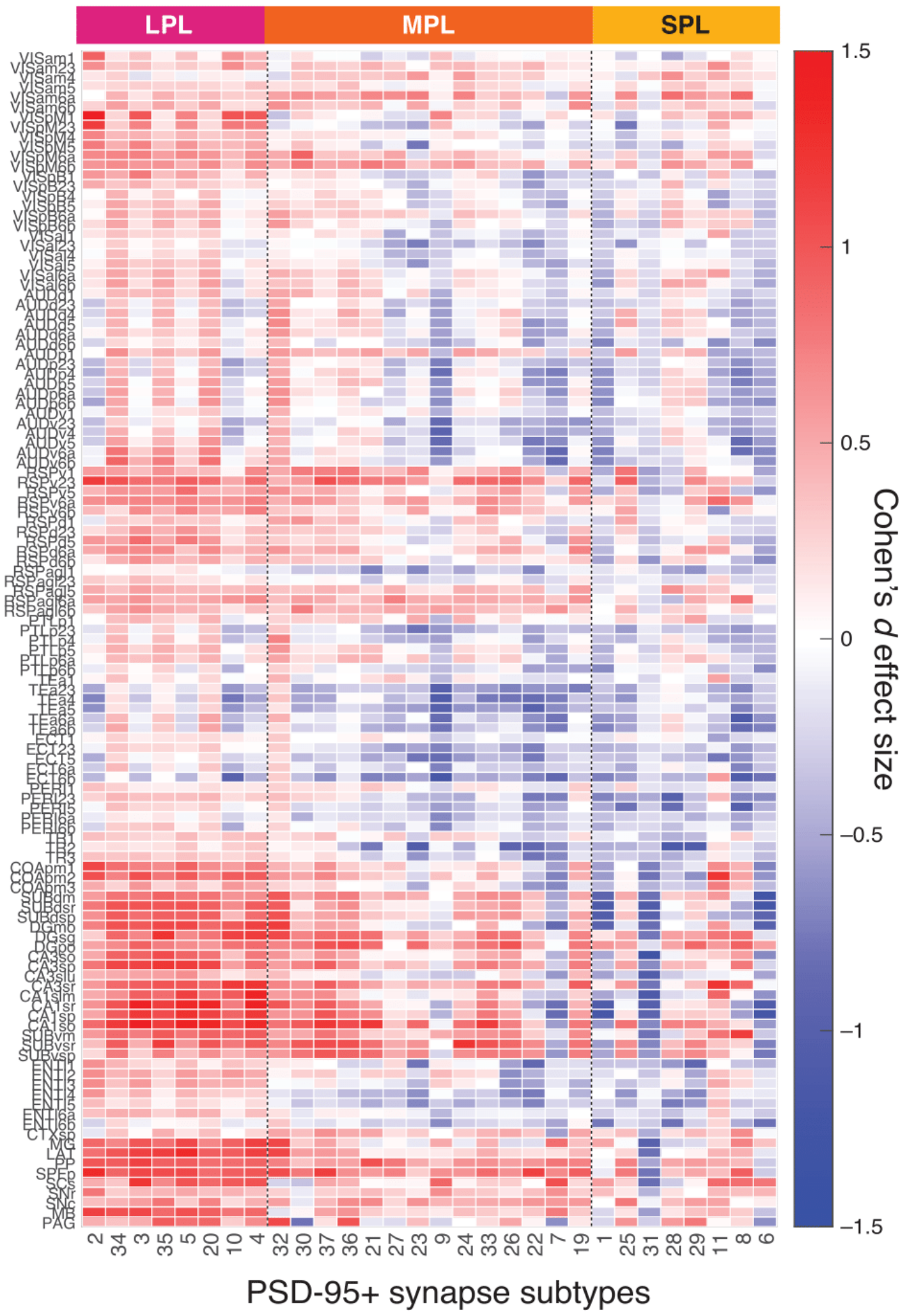

Fig. S24) Cohen's *d* effect size heatmaps showing hemisphere-specific, MD-driven shifts in synapse subtypes along a gradient of synapse protein lifetime (related to Fig. 3B). MD resulted in a higher density of long protein lifetime (LPL) synapses in the monocular visual area, retrosplenial areas, the hippocampus and the thalamus in the contralateral hemisphere, when normalised by summed hemisphere values. This lateralised reorganisation is reminiscent of changes observed following sleep deprivation[1]. For brain region abbreviations see supplementary material 'Brain region list'.

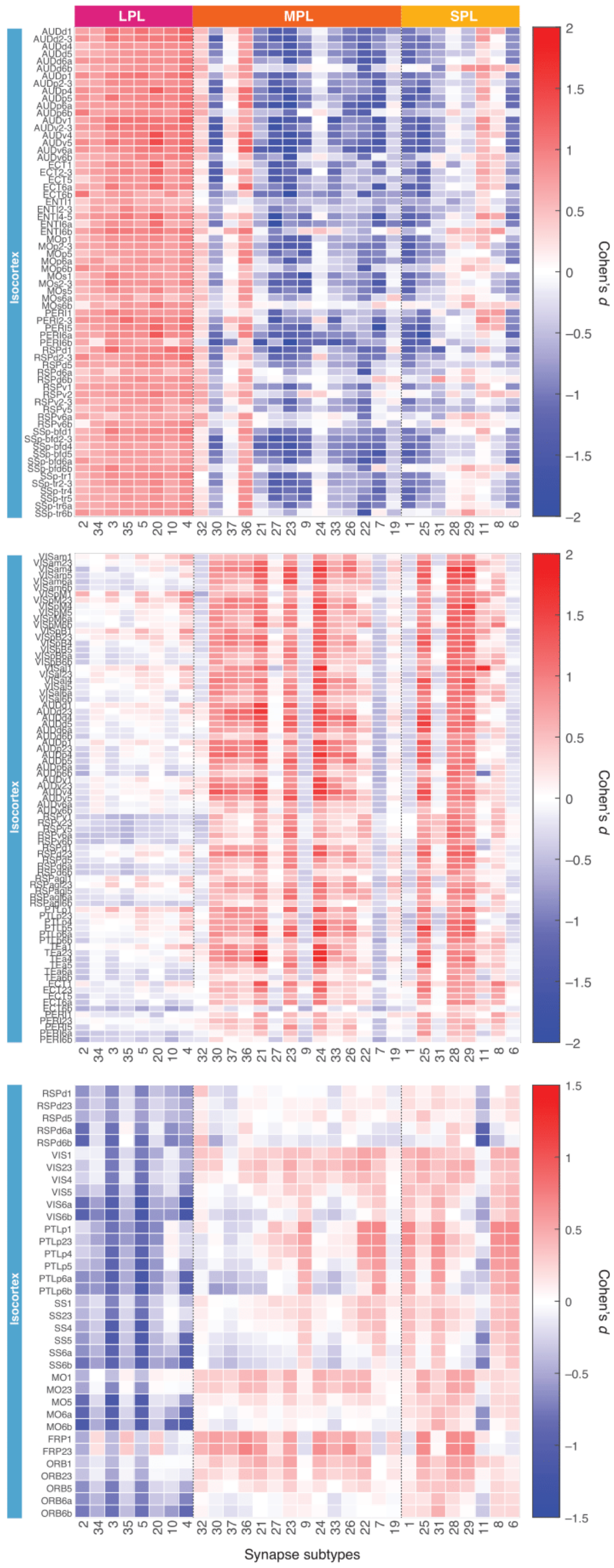

Fig. S25) Regional effect sizes (Cohen's *d*) for 30 PSD95$^+$ synapse subtypes across the isocortex, ordered by protein lifetime, comparing sleep-deprived mice and controls (top), MD mice and non-deprived controls (middle), and developmental EE and SC control mice (bottom). The sleep deprivation graph was reproduced from previously published data[1]. For brain region abbreviations see supplementary material 'Brain region list'.

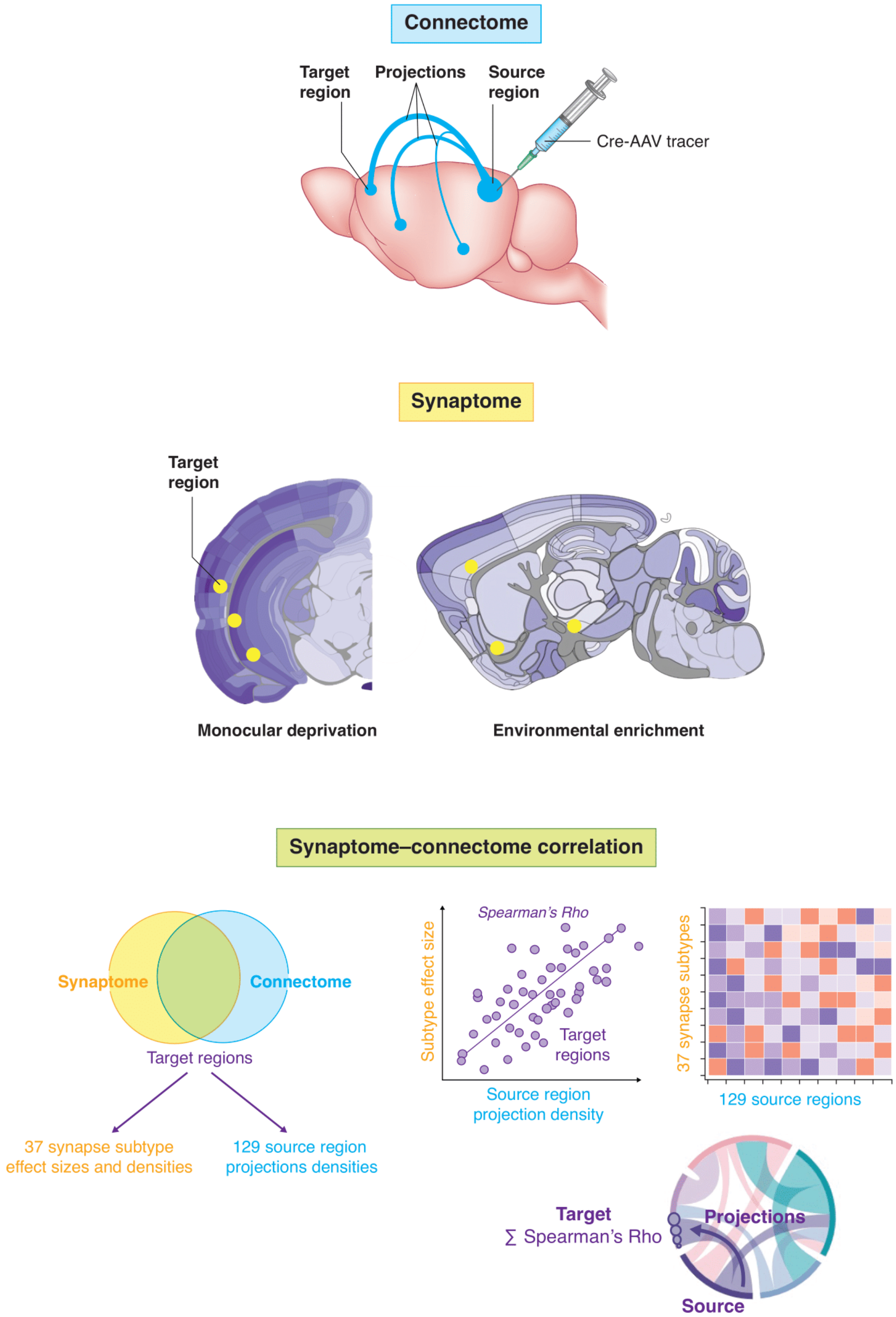

Fig. S26) Overview of the synaptome–connectome correlation analysis. **Top:** Illustration of the tracing methodology used to produce the connectome datasets[2], showing anterograde projection densities from each injected source region to all delineated target regions. **Middle:** Synaptome mapping provides regional synapse subtype density profiles and experimental effect sizes. **Bottom:** The two datasets are integrated across matched target regions to correlate projection strength from 129 source regions with either baseline subtype densities or Cohen's *d* effect sizes for 37 subtypes. Representative outputs—scatter plot, heatmap, and network diagram—demonstrate result formats.

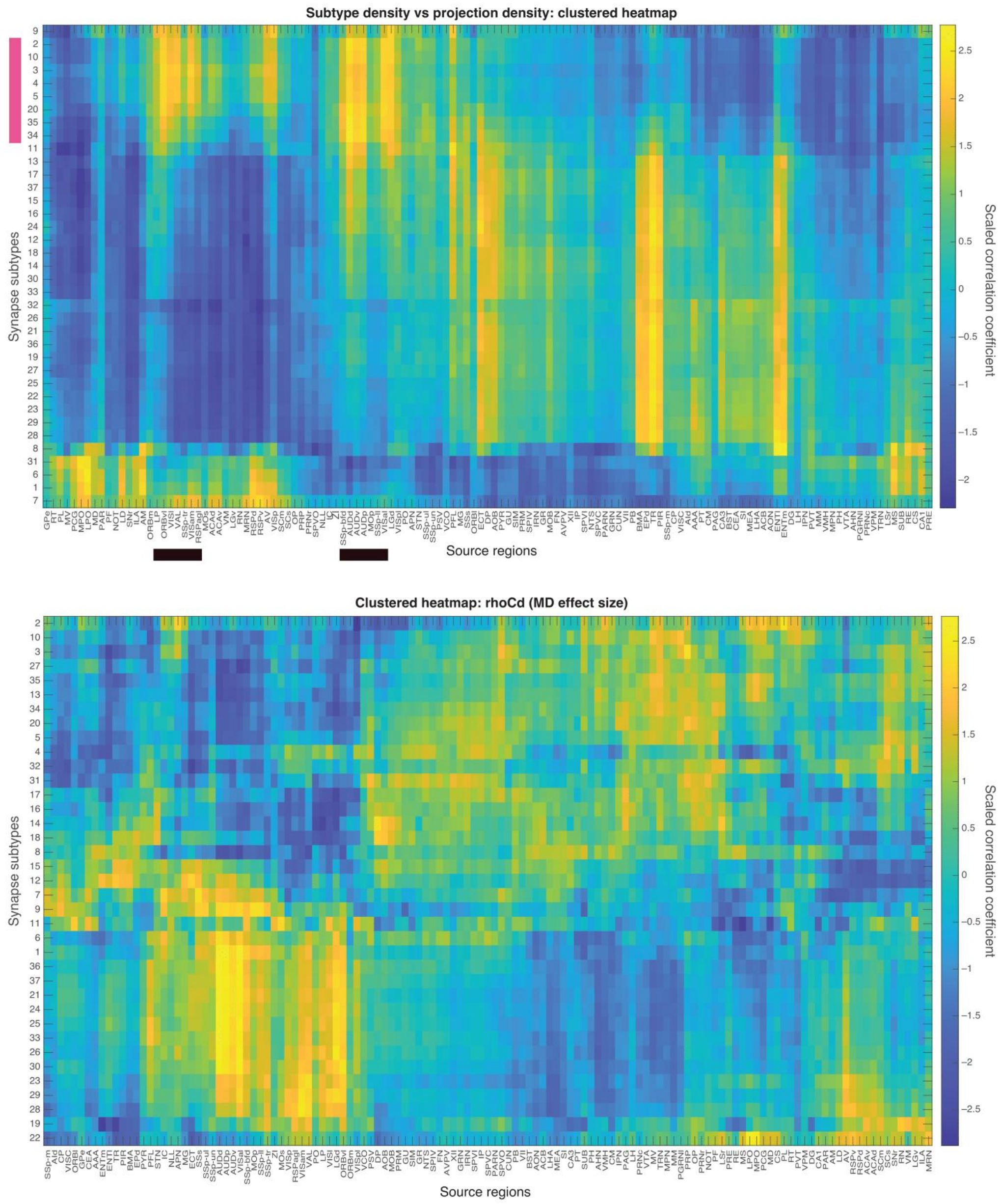

Fig. S27) Top: Hierarchically clustered heatmaps of Spearman's rho values correlating subtype densities (1-month control mice) with source region projection densities across target regions. The pink bar on the y-axis marks the cluster of long-protein lifetime synapses which positively correlate with several thalamocortical sensory areas, as indicated with the black bar on the x-axis. Bottom: Hierarchically clustered heatmaps of Spearman's rho values correlating subtype effect size (MD paradigm) with source region projection densities across target regions (related to Fig. 4A). Rows represent 37 synapse subtypes; columns are anatomically defined source regions. For brain region abbreviations see supplementary material 'Brain region list'.

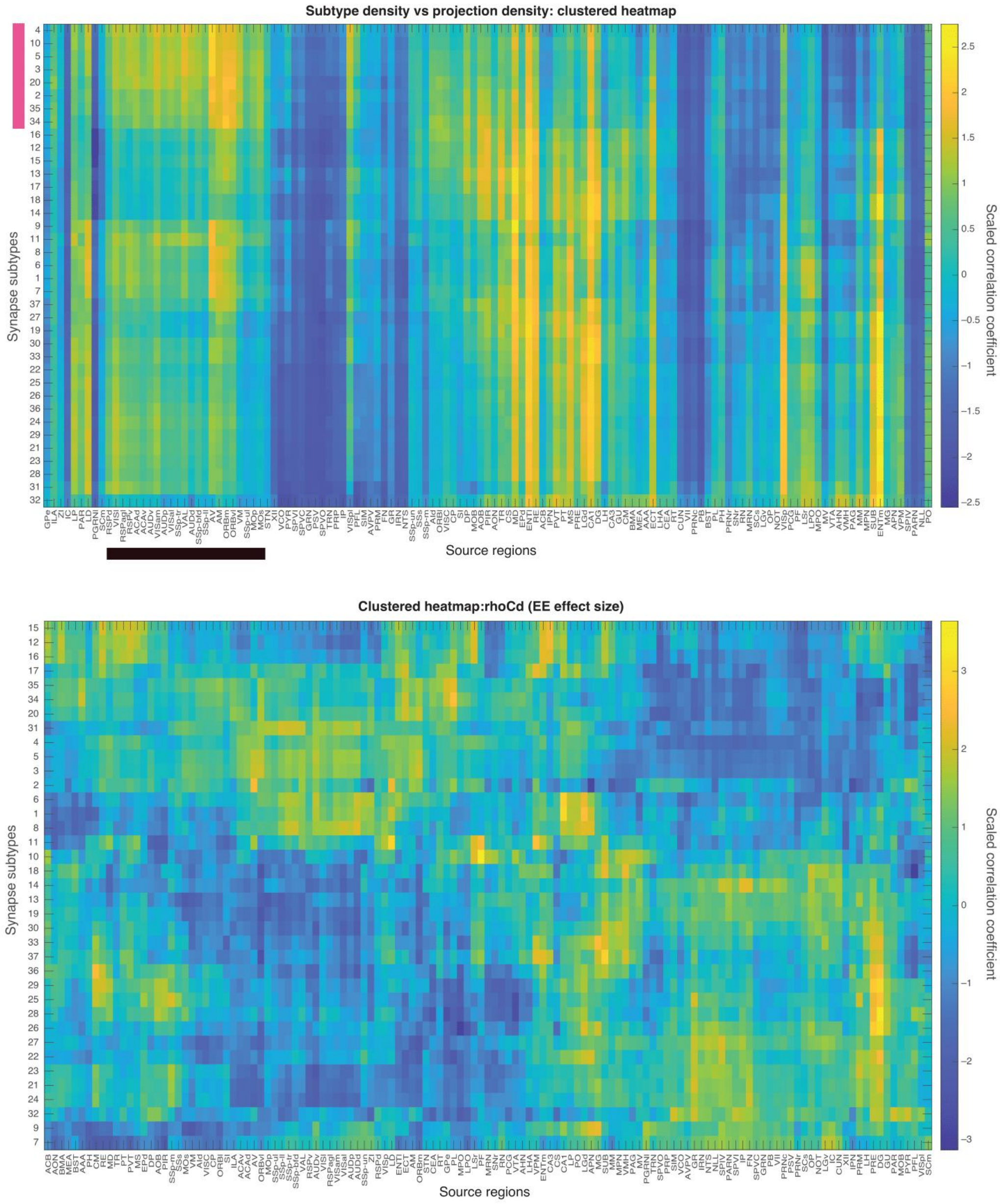

Fig. S28) Top: Hierarchically clustered heatmaps of Spearman's rho values correlating subtype densities (3-month control mice) with source region projection densities across target regions. The pink bar on the y-axis marks the cluster of long-protein lifetime synapses which positively correlate with several thalamocortical sensory areas, as indicated with the black bar on the x-axis. Bottom: Hierarchically clustered heatmaps of Spearman's rho values correlating subtype effect size (developmental EE paradigm) with source region projection densities across target regions (related to Fig. 4G). Rows represent 37 synapse subtypes; columns are anatomically defined source regions. For brain region abbreviations see supplementary material 'Brain region list'.

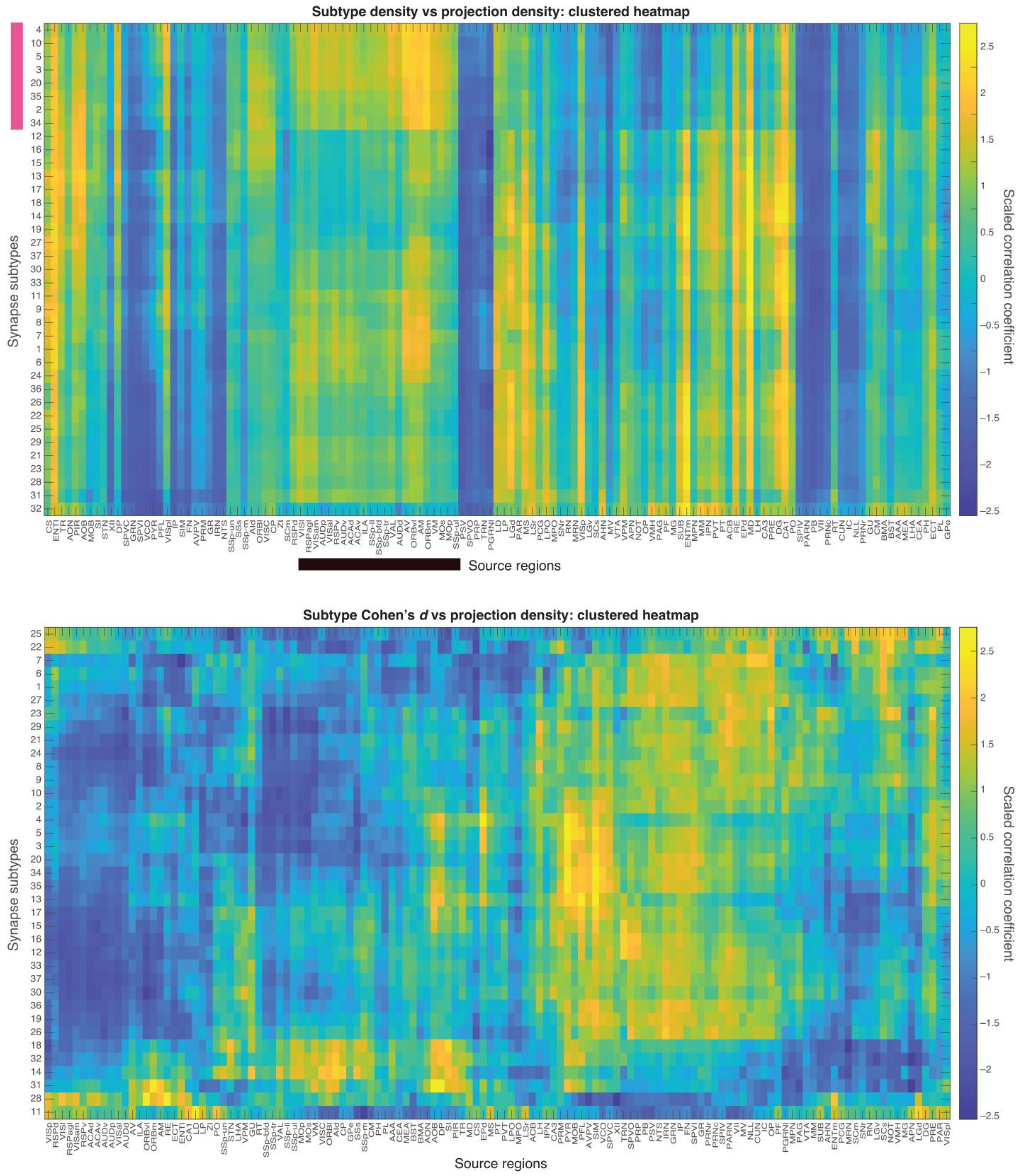

Fig. S29) Top: Hierarchically clustered heatmaps of Spearman's rho values correlating subtype densities (6- to 12-month control mice) with source region projection densities across target regions. The pink bar on the y-axis marks the cluster of long-protein lifetime synapses which positively correlate with several thalamocortical sensory areas, as indicated with the black bar on the x-axis. Bottom: Hierarchically clustered heatmaps of Spearman's rho values correlating subtype effect size (adult EE paradigm) with source region projection densities across target regions. Rows represent 37 synapse subtypes; columns are anatomically defined source regions. For brain region abbreviations see supplementary material 'Brain region list'.

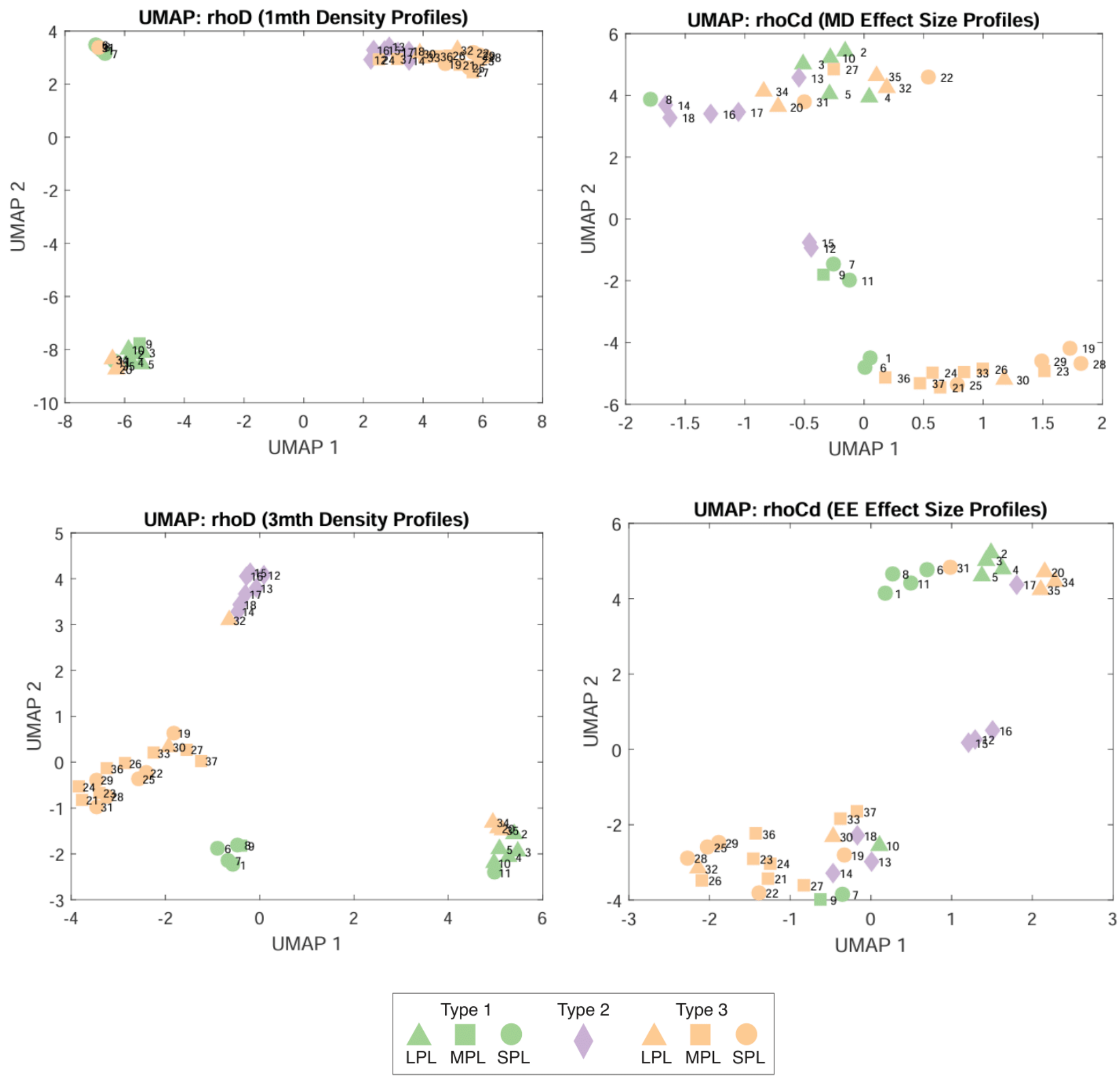

Fig. S30) UMAP projections of synapse subtype correlation profiles with anatomical connectivity. UMAP was applied to z-scored Spearman's rho correlation matrices linking projection density to either baseline subtype density (ρ^D; top-left, bottom-left) or Cohen's *d* effect size (ρ^{cd}; top-right, bottom-right). (Top-left, bottom-left): UMAP projections of ρ^D in 1-month (top) and 3-month (bottom) control mice. (Top-right, bottom-right): UMAP projections of ρ^{cd} in MD (top) and developmental EE (bottom). Marker colour and shape indicate synapse type and protein lifetime class. UMAP parameters (k = 10 for MD, 8 for EE) were selected based on preservation of global structure and local fidelity. Full embedding metrics for both UMAP and t-SNE are provided in the Source Data files.

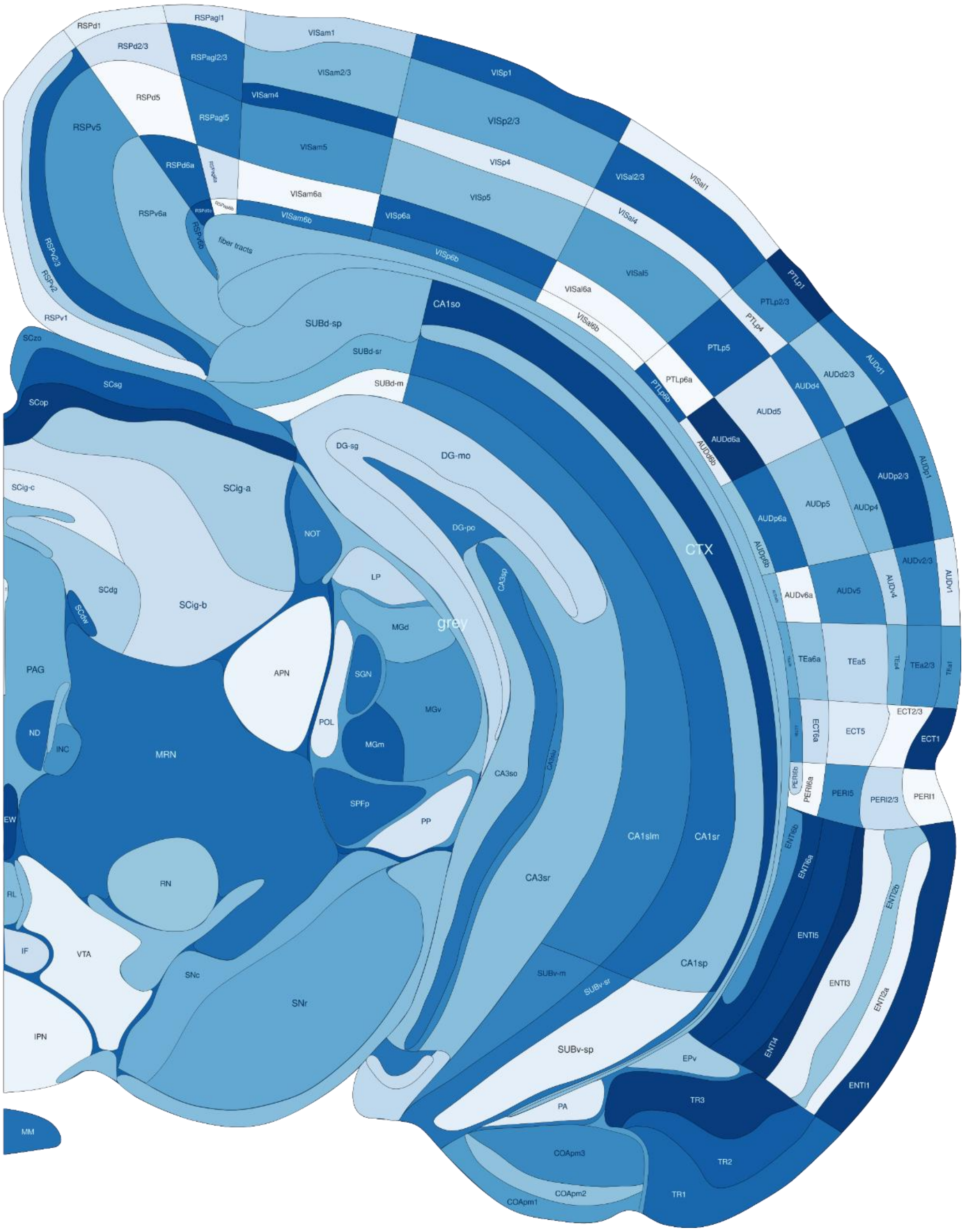

Fig. S31) Coronal brain map with subregion labels. This figure was generated using a mapping tool developed in house based on brain atlases from the Allen Institute[3]. For brain region abbreviations see supplementary material 'Brain region list'.

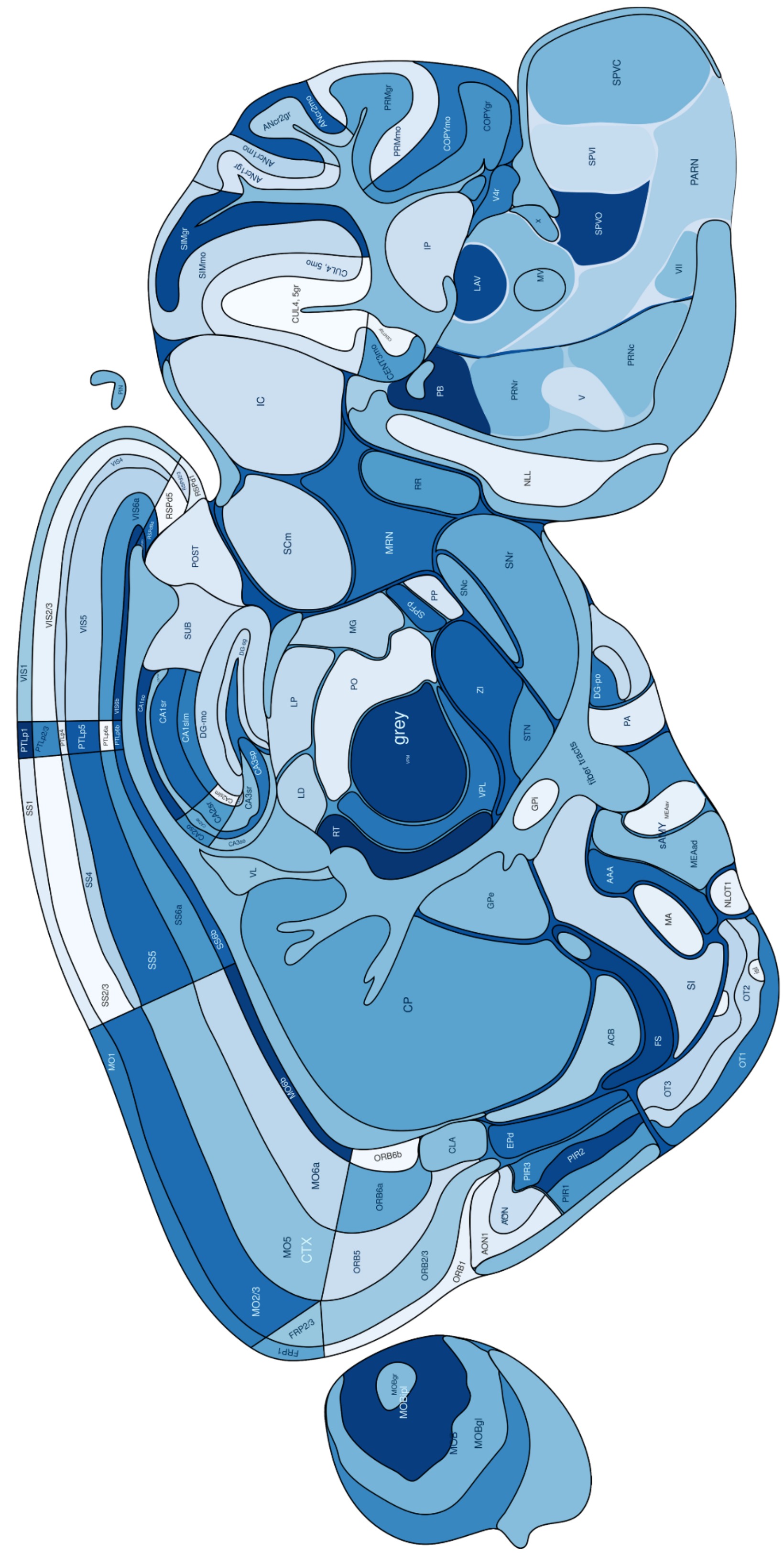

Fig. S32) Parasagittal brain map with subregion labels. This figure was generated using a mapping tool developed in house based on brain atlases from the Allen Institute[3]. For brain region abbreviations see supplementary material 'Brain region list'.

Brain Region List

Acronym	Region name
ACB	Nucleus accumbens
AAA	Anterior amygdalar area
ACAd	Anterior cingulate area, dorsal part
ACAv	Anterior cingulate area, ventral part
ACB	Nucleus accumbens
AHN	Anterior hypothalamic nucleus
AId	Agranular insular area, dorsal part
AM	Anteromedial nucleus of the thalamus
ANcr1gr	Crus 1, granular layer
ANcr1mo	Crus 1, molecular layer
ANcr2gr	Crus 2, granular layer
ANcr2mo	Crus 2, molecular layer
AOB	Accessory olfactory bulb
AON	Anterior olfactory nucleus
APN	Anterior pretectal nucleus
AUDd	Dorsal auditory area
AUDp	Primary auditory area
AUDv	Ventral auditory area
AV	Anteroventral nucleus of the thalamus
AVPV	Anteroventral periventricular nucleus
BMA	Basomedial amygdalar nucleus
BS	Brainstem
BST	Bed nuclei of the stria terminalis
CA1	Field CA1
CA1so	Field CA1, stratum oriens
CA1sp	Field CA1, stratum pyramidale
CA1sr	Field CA1, stratum radiatum
CA3	Field CA3
CA3slu	Field CA3, stratum lucidum
CA3so	Field CA3, stratum oriens
CA3sp	Field CA3, stratum pyramidale
CA3sr	Field CA3, stratum radiatum
CB	Cerebellum
CEA	Central amygdalar area
CENTgr	Lobule III, granular layer
CENTmo	Lobule III, molecular layer
CLA	Claustrum
CM	Central medial nucleus of the thalamus
COApm	Cortical amygdalar area, posterior part, medial zone
COPYgr	Copula pyramidis, granular zone
COPYmo	Copula pyramidis, molecular zone
CP	Caudate putamen
CS	Superior central nucleus raphe
CTX	Isocortex
CTXsp	Cortical subplate
CUL45gr	Lobules IV-V, granular layer

CUL45mo	Lobules IV-V, molecular layer
CUN	Cuneiform nucleus
DG	Dentate gyrus
DGmo	Dentate gyrus, molecular layer
DGpo	Dentate gyrus, polymorph layer
DGsg	Dentate gyrus, granule layer
DP	Dorsal peduncular area
ECT	Ectorhinal area
ENTl	Entorhinal area, lateral part
ENTm	Entorhinal area, medial part
EPd	Endopiriform nucleus, dorsal part
FN	Fastigial nucleus
FRP	Frontal pole
GPe	Global pallidus, external segment
GR	Gracile nucleus
GRN	Gigantocellular reticular nucleus
GU	Gustatory areas
HPF	Hippocampal formation
HY	Hypothalamus
IC	Inferior colliculus
ILA	Infralimbic area
IP	Interposed nucleus
IPN	Interpeduncular nucleus
IRN	Intermediate reticular nucleus
Isl	Islands of Cajella
LAT	Lateral group of the dorsal thalamus
LD	Lateral dorsal nucleus of the thalamus
LGd	Dorsal part of the lateral geniculate complex
LGv	Ventral part of the lateral geniculate complex
LH	Lateral habenula
LHA	Lateral hypothalamuc area
LP	Lateral posterior nucleus of the thalamus
LPO	Lateral preoptic area
LSr	Lateral septal nucleus, rostral part
LTD	Long-term depression
LTP	Long-term potentiation
MB	Midbrain
MD	Mediudorsal nucleus of the thalamus
MEA	Medial amygdalar area
MG	Medial geniculate complex
MM	Medial mammillary nucleus
MO	Somatomotor areas
MOB	Main olfactory bulb
MOp	Primary motor area
MOs	Secondary motor area
MPN	Medial preoptic nucleus
MPO	Medial preoptic area
MRN	Midbrain reticular nucleus

MS	Medial septal nucleus
MV	Medial vestibular nucleus
MY	Medulla
NLL	Nucleus of the lateral lemniscus
NOT	Nucleus of the optic tract
NTS	Nucleus of the solitary tract
OLF	Olfactory areas
OP	Olivary pretectal nucleus
ORB	Orbital cortex
ORBl	Orbital area, lateral part
ORBm	Orbital area, medial part
ORBvl	Orbital area, ventrolateral part
OT	Olfactory tubercle
P	Pons
PAG	Periaqueductal gray
PAL	Pallidum
PAR	Parasubiculum
PARN	Parvicellular reticular nucleus
PB	Parabrachial nucleus
PCG	Pontine central gray
PERI	Perirhinal area
PF	Parafascicular nucleus
PFL	Paraflocculus
PGRNl	Paragigantocellular reticular nucleus, lateral part
PH	Posterior hypothalamic nucleus
PIR	Piriform area
PL	Prelimbic area
PO	Posterior complex of the thalamus
POST	Post-subiculum
PP	Peripeduncular nucleus
PRE	Presubiculum
PRM	Paramedian lobule
PRMgr	Paramedian lobule, granular layer
PRMmo	Paramedian lobule, molecular layer
PRNc	Pontine reticular nucleus, caudal part
PRNr	Pontine reticular nucleus
PRP	Nucleus prepositus
PSV	Principal sensory nucleus of the trigeminal
PT	Parataenial nucleus
PTLp	Posterior parietal association areas
PVT	Paraventricular nucleus of the thalamus
PYR	Pyramus (VIII)
RE	Nucleus of reunions
RN	Red nucleus
RSPagl	Retrosplenial area, lateral agranular part
RSPd	Retrosplenial area, dorsal part
RSPv	Retrosplenial area, ventral part
RT	Reticular nucleus

RT	Reticular nucleus of the thalamus
sAMY	Striatum-like amygdalar nuclei
SCm	Superior colliculus, motor related
SCs	Superior colliculus, sensory related
SI	Substantia innominata
SIM	Simple lobule
SIMgr	Simple lobule, granular layer
SIMmo	Simple lobule, molecular layer
SNr	Substantia nigra, reticular part
SPFp	Subparafascicular nucleus, parvicellular part
SPIV	Spinal vestibular nucleus
SPVC	Spinal nucleus of the trigeminal, caudal part
SPVI	Spinal nucleus of the trigeminal, interpolar part
SPVO	Spinal nucleus of the trigeminal, oral part
SS	Somatosensory cortex
SSp-bfd	Primary somatosensory area, barrel field
SSp-ll	Primary somatosensory area, lower limb
SSp-m	Primary somatosensory area, mouth
SSp-tr	Primary somatosensory area, trunk
SSp-ul	Primary somatosensory area, upper limb
SSp-un	Primary somatosensory area, unassigned
SSs	Supplemental somatosensory area
STN	Subthalamic nucleus
SUB	Subiculum
SUBdm	Subiculum, dorsal part, molecular layer
SUBdsp	Subiculum, dorsal part, pyramidal layer
SUBdsr	Subiculum, dorsal part, stratum radiatum
SUBvm	Subiculum, ventral part, molecular layer
SUBvsp	Subiculum, ventral part, pyramidal layer
SUBvsr	Subiculum, ventral part, stratum radiatum
Tea	Temporal association areas
TH	Thalamus
TR	Postpiriform transition area
TRN	Tegmental reticular nucleus
VAL	Ventral anterior-lateral complex of the thalamus
VCO	Ventral cochlear nucleus
VII	Facial motor nucleus
VIS	Visual cortex
VISal	Anterolateral visual area
VISam	Anteromedial visual area
VISC	Visceral area
VISl	Lateral visual area
VISp	Primary visual area
VISpB	Primary visual cortex, binocular zone
VISpl	Posterolateral visual area
VISpM	Primary visual cortex, monocular zone
VM	Ventral medial nucleus of the thalamus
VMH	Ventromedial hypothalamic nucleus

VPL	Ventral posterolateral nucleus of the thalamus
VPM	Ventral posteromedial nucleus of the thalamus
VTA	Ventral tegmental area
XII	Hypoglossal nucleus
ZI	Zona Incerta

Supplementary References

1 Koukaroudi, D. *et al*. Sleep maintains excitatory synapse diversity in the cortex and hippocampus. *Current Biology* **34**, 3836-3843. e3835 (2024).

2 Science, A. I. f. B. *Allen Mouse Brain Connectivity Atlas*, <https://connectivity.brain-map.org/> (

3 Science, A. I. f. B. *Allen Reference Atlas – Mouse Brain*, <https://atlas.brain-map.org/> (
