# Supplementary material for: Experience modifies the synaptome architecture in the developing and adult mouse brain
Source: Nat Commun. 2026 Nov 11;17:11204. doi: 10.1038/s41467-026-78702-7 (PMC13754844; doi:10.1038/s41467-026-78702-7)
Supplement: Supplementary file 2 [file 41467_2026_78702_MOESM2_ESM.pdf]

nature portfolio

Corresponding author(s): Prof Seth Grant

Last updated by author(s): Sep 2, 2026

Reporting Summary

Nature Portfolio wishes to improve the reproducibility of the work that we publish. This form provides structure for consistency and transparency in reporting. For further information on Nature Portfolio policies, see our Editorial Policies and the Editorial Policy Checklist.

Statistics

For all statistical analyses, confirm that the following items are present in the figure legend, table legend, main text, or Methods section.

n/a	Confirmed	
☐	☒	The exact sample size (*n*) for each experimental group/condition, given as a discrete number and unit of measurement
☐	☒	A statement on whether measurements were taken from distinct samples or whether the same sample was measured repeatedly
☐	☒	The statistical test(s) used AND whether they are one- or two-sided<br>*Only common tests should be described solely by name; describe more complex techniques in the Methods section.*
☐	☒	A description of all covariates tested
☐	☒	A description of any assumptions or corrections, such as tests of normality and adjustment for multiple comparisons
☐	☒	A full description of the statistical parameters including central tendency (e.g. means) or other basic estimates (e.g. regression coefficient) AND variation (e.g. standard deviation) or associated estimates of uncertainty (e.g. confidence intervals)
☐	☒	For null hypothesis testing, the test statistic (e.g. *F*, *t*, *r*) with confidence intervals, effect sizes, degrees of freedom and *P* value noted<br>*Give P values as exact values whenever suitable.*
☐	☒	For Bayesian analysis, information on the choice of priors and Markov chain Monte Carlo settings
☐	☒	For hierarchical and complex designs, identification of the appropriate level for tests and full reporting of outcomes
☐	☒	Estimates of effect sizes (e.g. Cohen's *d*, Pearson's *r*), indicating how they were calculated

Our web collection on statistics for biologists contains articles on many of the points above.

Software and code

Policy information about availability of computer code

Data collection	Imaging was acquired using Nikon NIS-Elements (v5.20.1-5.21.1) automated JOBS routines; whole-section imaging and metadata/tile handling used NIS-Elements Advanced Research (v5.21).
Data analysis	Image processing, montage reconstruction and synaptome mapping used MATLAB (R2020b–R2022a), Python (≥3.8 for the synaptome mapping pipeline; 3.13 for connectome analyses), ImageJ (v2.14.0), napari (v0.5.5) and TrackMate (ImageJ, v2.0.0) with custom scripts. Statistical analyses used MATLAB and R (v4.4.0), with Bayesian models implemented in JAGS via runjags (R) and matjags (MATLAB). Code developed for synaptome mapping and analyses is available in Git and BioStudies repositories cited in the Key Resources Table. Source data for main and supplementary figures are provided with this paper. Additional information required to re-analyse the data is available from the lead contact upon request.

For manuscripts utilizing custom algorithms or software that are central to the research but not yet described in published literature, software must be made available to editors and reviewers. We strongly encourage code deposition in a community repository (e.g. GitHub). See the Nature Portfolio guidelines for submitting code & software for further information.

Data

Policy information about availability of data

All manuscripts must include a data availability statement. This statement should provide the following information, where applicable:

- Accession codes, unique identifiers, or web links for publicly available datasets
- A description of any restrictions on data availability
- For clinical datasets or third party data, please ensure that the statement adheres to our policy

All data have been deposited on BioStudies and are publicly available; DOIs are listed in the Key Resources Table, including https://www.ebi.ac.uk/biostudies/studies/S-BSST2147, with associated files available via the BioStudies FTP directory. All imaging data, posterior distributions (MCMC draws), PCA outputs, projection matrices, synaptome–connectome mapping tables, and embedding outputs are deposited there. Source data for main and supplementary figures are provided with this paper.

Research involving human participants, their data, or biological material

Policy information about studies with human participants or human data. See also policy information about sex, gender (identity/presentation), and sexual orientation and race, ethnicity and racism.

Reporting on sex and gender	n/a
Reporting on race, ethnicity, or other socially relevant groupings	n/a
Population characteristics	n/a
Recruitment	n/a
Ethics oversight	n/a

Note that full information on the approval of the study protocol must also be provided in the manuscript.

Field-specific reporting

Please select the one below that is the best fit for your research. If you are not sure, read the appropriate sections before making your selection.

☒ Life sciences ☐ Behavioural & social sciences ☐ Ecological, evolutionary & environmental sciences

For a reference copy of the document with all sections, see nature.com/documents/nr-reporting-summary-flat.pdf

Life sciences study design

All studies must disclose on these points even when the disclosure is negative.

Sample size	Sample sizes were: MD n=13 vs control n=14; developmental EE n=23 vs SC n=29; adult EE n=20 vs SC n=20. No statistical method was used to pre-determine sample size.
Data exclusions	Some animals/brains were not processed through imaging/analysis due to pre-analysis technical issues (e.g., dissection/sectioning damage preventing atlas-matched sections, or persistent out-of-focus scans), or because surplus control animals were not processed (pre-sectioning downsampling) to keep group sizes comparable; final analysed n are reported in the Methods/Results. Imaging scans with extensive out-of-focus regions were re-acquired using adjacent sections prior to analysis. During synaptome mapping, puncta were excluded if <4 pixels or >140 pixels, if classifier confidence was <10%, or if puncta intersected tile edges (to minimise border artefacts). Some subregions were not available for analysis in some animals due to small size or delineation constraints; subregion-level n are provided in the Source Data.
Replication	All key analyses were performed across independent biological replicates (individual mice) within each experimental condition and matched controls. EE animals were collected across multiple days (dependent on birth date). MD was performed in multiple independent cohorts; data were analysed jointly and results were consistent across cohorts. Analyses were performed at the animal level, with puncta aggregated within brain subregions per animal.
Randomization	For adult enrichment, female mice were randomly assigned to enriched or standard housing. For monocular deprivation, mice were assigned to achieve balanced male–female ratios across MD and control groups. For developmental enrichment, pregnant dams were distributed into EE or SC housing 7 days pre-parturition, and offspring remained in their assigned condition post-weaning.
Blinding	For generation of the puncta-detection training dataset, five independent raters manually annotated sub-tiles blinded to the study aims. For adult EE and MD cohorts, the experimenter was blinded to group allocation during sectioning, imaging and analysis. Blinding was maintained using coded sample identifiers. For developmental EE, the experimenter was not blinded. All dissections were performed unblinded.

Reporting for specific materials, systems and methods

We require information from authors about some types of materials, experimental systems and methods used in many studies. Here, indicate whether each material, system or method listed is relevant to your study. If you are not sure if a list item applies to your research, read the appropriate section before selecting a response.

Materials & experimental systems

n/a	Involved in the study
☒	☐ Antibodies
☒	☐ Eukaryotic cell lines
☒	☐ Palaeontology and archaeology
☐	☒ Animals and other organisms
☒	☐ Clinical data
☒	☐ Dual use research of concern
☒	☐ Plants

Methods

n/a	Involved in the study
☒	☐ ChIP-seq
☒	☐ Flow cytometry
☒	☐ MRI-based neuroimaging

Animals and other research organisms

Policy information about studies involving animals; ARRIVE guidelines recommended for reporting animal research, and Sex and Gender in Research

Laboratory animals	Mouse lines included C57BL/6 backgrounds expressing Psd95eGFP/eGFP; Sap102mKO2/mKO2, with developmental EE/SC cohorts additionally crossed with a 129S5 Glun1TAP/TAP strain as described. MD and developmental EE included male and female mice; adult EE used female C57BL/6J mice aged 6–12 months.
Wild animals	No wild animals were used.
Reporting on sex	Sex was reported per cohort (e.g., MD: 7/13 female; controls: 7/14 female; developmental EE: 12/23 female; SC: 15/29 female). Adult EE included female mice only (male mice were not in experimental design due to aggression risk in late transfer to enriched group housing). Sex was modelled in factorial Bayesian analyses where applicable.
Field-collected samples	No field-collected samples were used.
Ethics oversight	Animal experiments were carried out according to UK Home Office regulations and authorised by the Edinburgh University Director of Biological Services and the Joint Biological Services of Cardiff University.

Note that full information on the approval of the study protocol must also be provided in the manuscript.

Plants

Seed stocks	n/a
Novel plant genotypes	n/a
Authentication	n/a
