# Supplementary material for: Experience modifies the synaptome architecture in the developing and adult mouse brain
Source: Nat Commun. 2026 Nov 11;17:11204. doi: 10.1038/s41467-026-78702-7 (PMC13754844; doi:10.1038/s41467-026-78702-7)
Supplement: Supplementary file 3 [file 41467_2026_78702_MOESM3_ESM.pdf]

nature portfolio

Peer Review File

Experience and sensory input reconfigure synaptome architecture of the developing and mature brain

Corresponding Author: Professor Seth Grant

This file contains all reviewer reports in order by version, followed by all author rebuttals in order by version.

Version 1:

Reviewer comments:

Reviewer #1

(Remarks to the Author)
This manuscript investigates 'synaptome architecture' and the impact of experience in mice. The authors report that environmental enrichment (EE) and monocular visual deprivation (MD) produce widespread and subtype-selective reorganisation of excitatory synapses in the mouse brain, including >100 brain regions. They find that experience selectively alters subtypes of synapses, and associate this with protein turnover rates and connectome architecture. This experience-dependent 'synaptome plasticity' occurs during development and adulthood. They propose a population-selection model whereby such experiences specifically alter selective synapse varieties, and thus create a distributed trace of past experience.

These are novel findings. The manuscript is generally well written and the data clearly presented. I have suggestions below as to how the manuscript could be improved.

1. The title states that 'Experiences are encoded'. It is not clear that the authors have demonstrated that the changes they observe are encoding the experiences. More evidence would required to demonstrate a causative relationship. Or the title could be changed to reflect exactly what has been demonstrated in the present manuscript.

2. The last sentence of the Abstract states that 'Our results also point to synaptome architecture as a shared framework integrating experience, lifespan changes, sleep, genetic variation and disease'. Whilst the present manuscript is highly informative regarding the effects of experience on synaptome architecture, it does not show that this shared framework is involved in sleep, genetic variation, disease, etc. Some rewording of this last sentence of the Abstract could draw the focus back to what has been demonstrated in the present manuscript.

3. In the Monocular Deprivation (MD) experiment, did the authors provide sham anaesthesia, analgesia to the control mice? If not, could some of the observed changes be due to extra handling, anaesthesia, analgesia, the stress of surgery, etc.?

4. Regarding the above point, could more analysis be done in the MD mouse brains comparing the two hemispheres for relevant visual areas, to establish what impacts are due to visual deprivation per se, and which might be due to differential handling, anaesthesia, algesia, surgical stress, etc.?

5. Following up the above points on MD, it is stated that there were many changes induced by MD outside the visual areas, and not specific to contralateral hemisphere. This could be explained if the controls were not 'sham controls' and were not exposed to anaesthesia, analgesia and surgical stress/handling. This is a key issue the authors need to address. If the controls were not exposed to anaesthesis, analgesia, etc., it may also be that they were more active in the home cage (relative to MD mice recovering post-surgery) and had altered circadian/sleep rhythms, etc.

6. The authors describe Bayesian Analysis and need to provide more methodological details of exactly what this involved.

7. It is not exactly clear how the 37-subtype classification system was established. More detail would be welcome.

8. The EE or SC conditions were for 3 months from a week before birth (including EE of both dams and embryos/pups until weaning). It is inaccurate to call it juvenile EE, as the juvenile period in mice is often considered somewhere in the range of

4-10 weeks, depending on the definition. However, most researchers routinely define mice as adult after two months of age.

9. When EE was initiated approximately 7 days before birth (according to the protocol provided), did this disrupt dam-pup interactions? Could some of the effects be due to stress of imposing such an environmental change during the last week of pregnancy, and throughout weaning period? Could some effects be indirect, due to changes in maternal care, lactation, suckling, etc., rather than impacts on the offspring (from neonatal to young adult stages) themselves? Could these disruptive effects on the dams have disrupted brain development (including synaptogenesis) in the last week of pregnancy, and into the postnatal period?

10. How did the authors determine the time frame of EE (three months for young cohort and one month for the older cohort)?

11. Do the authors have information on how much the EE mice (both developmental and adult EE) interacted with the EE objects (in the mazes, etc.), and how much physical activity they had (including on running wheels), relative to SC controls?

12. EE protocols are intrinsically variable and difficult to reproduce. Even superficially similar EE protocols do not always have the same effects on brain and behaviour, so references to the effects of EE in prior publications may not reflect the protocol used in this study. It would be good to know if the authors, using the EE in this manuscript, had observed specific changes in behaviour (and/or cellular/molecular changes beyond the reported synaptome mapping), including cognitive performance, for example.

13. The authors state that 'These findings suggest that EE does not broadly promote global synaptogenesis'. However, this was from one EE protocol (commercial Marlau cages), started at birth and continued to 3 months of age. For all of the reasons listed above (including the possibility that starting EE at birth stressed both the dams and pups and altered development) I don't think this one finding demonstrates that EE does not promote global synaptogenesis. I suggest such language be toned down a little.

14. Furthermore, do the authors have more molecular data (e.g. transcriptomics or proteomics) that might inform molecular mechanisms mediating the observed synaptome plasticity.

15. The authors state that they excluded males due to fighting in male cages in the 'adult EE' experiments (consistent with some previous literature in this field). This leads to a limitation in being unable to address sex effects in this female-only adult EE experiment. Is there a way around this limitation (for example, not pooling adult males that are not littermates, to avoid the fighting that might be result of establishing new dominance heirarchies amongst stranger mice)?

16. How did the authors control for any variability in image background between batches (which might affect detectability of synapses at threshold, etc.)? Were there batch effects?

17. In the Discussion, there are broad statements made regarding the effects of EE, but it should be noted that the authors used a very specific type of EE, Marlau cages (sold commercially). They provide access to 3 running wheels per Marlau cage, and different configurations of a maze that was changed every two days. It is possible that mice spent most of their time on running wheels. And the novelty of the changes to this one maze may have worn out after a week or two. So the relative novelty and complexity in the EE cages (over time) in the present study, and the relative amounts of physical activity (versus sensory and cognitive stimulation) may vary widely from other published EE studies (where the developmental starting points and duration of EE also vary widely) with which the authors are make their comparisons. The 'devil may be in the detail' in such EE protocols, and these findings of synaptome changes (over space and time) may not generalise to other protocols.

(Remarks on code availability)

Reviewer #2

(Remarks to the Author)
This study examines whether experience alters brain-wide "synaptome architecture" by quantifying the distribution of excitatory synapses classified by PSD95 and SAP102 expression. Using environmental enrichment and monocular deprivation paradigms, the authors report widespread but subtype-specific changes across more than 100 mouse brain regions. Contrary to earlier literature, total synapse density is largely unchanged or modestly reduced. Instead, experience appears to redistribute predefined synapse subtypes. These effects are reported during development and adulthood. Subtype sensitivity correlates with PSD95 protein lifetime and, more weakly, with connectome projections. The authors propose a population-selection model in which experience modifies synapse subtype frequencies rather than synaptic strength. The work is technically ambitious and data-rich, but interpretation rests heavily on two scaffold proteins. Conceptual overreach and limited mechanistic depth weaken the broader claims. Overall, the conclusions substantially exceed what the data convincingly demonstrate.

Major comments

1. Overall conceptual concern: overinterpretation from two proteins

A central issue is that the paper repeatedly frames its findings as "single-synapse resolution synaptome mapping", which is misleading. The entire classification rests on PSD95 and SAP102 only, two postsynaptic scaffold proteins. While important, these represent a tiny fraction of synaptic molecular diversity. Claims about "synapse proteomes," "synapse varieties," or "synaptome architecture" therefore substantially overreach the data. This terminology should be reworded throughout to

avoid implying molecular completeness.
2. Heavy inferences from descriptive redistribution
The study is almost entirely descriptive: synapse subtype densities are compared across conditions using effect sizes and clustering. Yet the Discussion repeatedly infers encoding of experience, memory traces, and adaptive selection mechanisms. These are speculative and not directly tested. No functional readout (electrophysiology, behavior linked to subtype shifts, causality) is provided. Statements suggesting that experience is “recorded” in synaptome architecture should be toned down or explicitly framed as hypotheses.
3. Discrepancy with prior EE literature
The authors report no increase in synapse density after environmental enrichment, in contrast to many previous studies. This discrepancy is acknowledged but not seriously addressed. A hypothesis is required. Possibilities include:
• PSD95/SAP102-based detection missing other synapse populations
• Enrichment driving synaptic pruning with refinement, not net growth
• Age, duration, or enrichment protocol differences
• Over-reliance on puncta-based density rather than ultrastructural synapse counts
Without such discussion, the result appears either underinterpreted or method-dependent.
4. Methods and representation are very hard to follow
The paper is exceptionally difficult to read, even for specialists. Core concepts—synapse types, subtypes, protein lifetime classes, PCA clustering, effect size heatmaps—are introduced piecemeal and assumed knowledge from prior publications. This manuscript must be self-contained.
Specifically:
• Synapse subtype classification should be explained from first principles in the main text, not as a follow-up shorthand.
• The reader should not need to know previous synaptome papers to understand what is being measured.
5. Statistical representation: Cohen’s d
Cohen’s d is used extensively but never properly explained. For a biology audience, this is problematic. What does a given effect size correspond to in biological terms? Why is Cohen’s d preferable to absolute or percentage change here? The authors should include a brief intuitive explanation and justify its use for cross-region comparisons.
6. Conceptual issue: comparing synapse subtypes across animals
It remains conceptually unclear how synapse subtypes are meaningfully compared across animals and conditions. Are subtypes assumed to be fixed molecular entities with stable identity? How robust is subtype assignment to variability in expression levels, imaging conditions, or normalization? This is a foundational assumption of the entire study and needs explicit justification.
7. Protein lifetime analysis: interesting but opaque
The protein lifetime result is arguably the most interesting aspect of the paper. However:
• It is poorly explained how PSD95 lifetime was estimated and linked to current data.
• Figures 3A and 3B are extremely hard to interpret, and the legends are insufficient.
• The logic connecting lifetime, subtype stability, and experience-driven change needs clearer exposition.
This section should be substantially rewritten for clarity.
8. Connectome alignment: correlation without causation
The integration with Allen connectivity data is technically impressive but conceptually weak. Correlation between projection density and subtype changes does not establish mechanistic alignment. The analysis risks appearing as post hoc pattern matching. The authors should more explicitly acknowledge limitations and avoid causal language.
9. Trivial or overstated conclusions
Several claims are either obvious or overstated:
• That experience activates distributed networks is not novel.
• That different experiences produce different molecular signatures is expected.
• The claim that subtype redistribution challenges classical synaptic plasticity models is incorrect; these frameworks are not mutually exclusive.
These points weaken the Discussion by overstating novelty.
10. Development vs genetics
The statement that synaptome architecture is primarily genome-encoded during development is misleading. Experience-dependent plasticity is central to developmental synapse organization. This section should be revised to better reflect the balance between genetic programs and experience.
Minor comments and presentation issues
• Figures are visually dense, difficult to decode, and insufficiently annotated.
• Heatmaps lack intuitive guidance for non-expert readers.
• The manuscript would benefit from summary schematics explaining the pipeline.
• Abbreviations and subtype numbers are overwhelming and poorly motivated.
• Language frequently inflates descriptive findings into conceptual claims.
Overall assessment and recommendation
This is a technically ambitious, data-heavy study that extends a previously established synaptome-mapping framework. However, the manuscript suffers from poor clarity, conceptual overreach, and limited mechanistic insight. Too many strong claims are based on redistribution of synapse classes defined by only two proteins. The most compelling result—the link to protein lifetime—is underdeveloped and obscured by presentation.

(Remarks on code availability)

Reviewer #3

(Remarks to the Author)
The article by Woods et al. reports the effects of enriched environment and 7 days of monocular deprivation during the critical period on the synaptic proteome of PSD95 and SAP102, which was monitored through fluorescent-tagged proteins from knock-in mouse lines. They report that either manipulation caused brain-wide changes, which were similar for the EE groups when performed throughout development or just for a month in adult mice. It is interesting to see so widespread changes, especially for a supposedly restricted manipulation of the visual system. This supports previous reports that plasticity does not occur just locally but rather brain-wide. This result further addresses the need for proper controls when, e.g., assigning specific brain areas to specific behaviors.
While the aim of the study is very laudable, several concerns remain.

1) Several confounding factors could influence the brain-wide changes. This includes but is not limited to the switch to single housing after MD surgery (this was not specified in the methods). Removing mice one by one for analysis leads to arousal of the remaining ones, which could influence brain-wide activity.

2) For the MD control, a sham surgery should be performed. It was not clear from the methods whether this was the case.

3) The EE mice were analyzed at an age of P81 to P96. These are mature mice. Thus, the claim of a conserved mechanism of synaptome plasticity across the lifespan appears overstated, as critical periods of the sensory areas end much earlier with likely different plasticity.

4) It is concerning that no clear effects of MD on contra- versus ipsilateral visual cortex were described. Differences have been widely reported, including on the proteome level. The results between the contra and ipsi visual cortex should be reported. The normalized data with comparisons between MD and control mice make it difficult to reveal such differences. Fig S9 and S10 are very similar, meaning after MD, contralateral and ipsilateral went through a similar reorganization process, but the brain reactions to external stimuli are very different in the contra vs ipsi hemisphere due to MD. Thus, it does not represent the physiological changes broadly observed.

5) The results were interpreted as experience-dependent plasticity. However, as presented, the conclusion is weak, as the changes might have a homeostatic origin, which would be equally likely. Manipulation such as EE or dark rearing affects E-I balance, which could lead to differences in network activity. E.g., it could be tested whether these changes prevail after mice are returned to SC or after the eyes are opened. Experience-dependent changes should prevail, while homeostatic ones should relax.

6) Fig 4 uses the baseline connectome to align with the change in synaptome. But if some connections are low at baseline but enhanced after EE or MD, using only the control connectome will miss this correlation. Fig 4A shows the correlation coefficient; if the negative number means negative correlation, that is also a strong relation. Both of these aspects need to be addressed.

7) More than 30 subtypes of synapses were distinguished. Specific changes in the density of some subtypes were reported. This should then lead to complementary changes in other subtypes. One wonders whether those numbers add up or whether just some change by a significant amount, and the rest is diluted in the many other types.

8) If these synaptome changes are characteristic of a behavioral manipulation, then one should be able to predict from the pattern of changes what experience the mouse had. Thus, one could test how accurately the synaptome of single mice would predict whether they belong to the control, MD, or EE group of the averaged dated of the other mice.

9) In the discussion, it is stated that the results “offer a new perspective on the classical model of synaptic plasticity”. The changes in synaptome could also represent different synaptic strengths, thus in accordance with classical synaptic plasticity models. With the data presented, I do not see how a firm conclusion could be drawn. Is the leading idea here that synapse type shifts occur through shifts in proteostasis? How would processes like synaptogenesis or pruning factor in?

10) The distribution of SAP102 and PSD95 does not solely occur through genetic mechanisms during development, as stated. Dr. Constantin-Paton and others have shown the activity/experience-dependent shift of PSD95 in and out of synapses. Thus, even initially, interactions between genetic factors and activity for synaptic proteins have been described. This part requires more elaboration.

11) Statements in lines 370-377 are not clear as it one hand highlights the effect on LPL subtypes showing their strongest associations with sensory pathways and then, in the next sentence, describe how, in the visual pathway, changes were driven in SPL and MPL subtypes. May need revision to be better understood.

(Remarks on code availability)

Version 2:

Reviewer comments:

Reviewer #1

(Remarks to the Author)
The authors have extensively revised the manuscript and adequately addressed almost all of my comments.

One comment, raised by myself and Reviewer 3, that for the MD experiment a sham-surgery control should have been used, has not yet been adequately addressed. The authors refer to the fact that in the 'MD field', sham surgeries are not often used. However, these studies (going back to Hubel and Weisel, and colleagues) usually only involved visual neurophysiology and used the unmanipulated eye/visual circuitry as an internal control. As the present MD experiment had a different design and different hypothesis (where the sham-surgery control becomes more crucial) the absence of this sham-surgery control remains a limitation.

Reviewer #2

(Remarks to the Author)
The authors have done a great jiob at improving their manuscript which is now much clearer and provide important insight. I have no additional comments

Reviewer #3

(Remarks to the Author)
I thank the authors for their detailed and largely constructive point-by-point response. The revision addresses most of my concerns satisfactorily, and I want to acknowledge specific improvements before addressing what remains outstanding.
The clarification on housing (point 1) is a straightforward factual correction that removes that confound from consideration. The expanded treatment of contralateral vs. ipsilateral effects (point 4) is a substantial improvement. The distinction between within-hemisphere effect-size heatmaps and inter-hemispheric balance analyses is now much clearer. I accept that partial convergence between hemispheres is a plausible outcome of unilateral deprivation rather than a failure of the method. I also appreciate the direct engagement with the subtype "conservation of mass" question (point 7) and the acknowledgment that PSD95/SAP102-negative synapses complicate any expectation of a fixed total. The response to point 10, incorporating literature on activity-dependent PSD95 redistribution, appropriately corrects the genetic/experiential framing.
Three of my original points are addressed by acknowledgment rather than resolution.
Sham-surgery controls (point 2). The authors' response, that the surgery was brief, performed six days before collection, and that one prior study using sham controls reported similarly broad effects, is a reasonable holding position, but it is not a resolution. It rests on a single external citation standing in for a control the present study itself lacks. Nonetheless, large-scale resource papers of this kind are valuable even before every confound is excluded. But I'd like to note that this leaves an open question about how much of the "brain-wide" MD signature reflects sensory deprivation specifically versus surgery, anaesthesia, or handling more generally. That distinction matters more here than in a typical single-region physiology study, because "brain-wide" is one of the paper's headline claims.
Homeostatic vs. experience-dependent origin (point 5). Similarly, the authors agree this is a live alternative explanation and defer the discriminating experiment (testing whether the effects relax after return to standard housing or after eye-opening) to future work. That is an honest and appropriately scoped response, but it means the paper's central interpretation, that this is "experience-dependent plasticity" in the sense usually invoked in the EE/MD literature, remains an assumption rather than a demonstrated result.
Predicting condition from single-mouse synaptome pattern (point 8). This is a missed opportunity, which could have been done with the existing data and strengthen the specificity of the observed changes.
With the revisions, the paper is now more accurately positioned as what it convincingly is: a technically ambitious, single-synapse-resolution atlas of brain-wide, subtype-selective synaptic reorganization following two well-chosen experiential manipulations. That is a substantial and citable contribution in its own right, and the revised framing does it justice rather than overselling it.
However, the three open points above are precisely the ones that would be needed to move this paper from a descriptive resource to a more causally and mechanistically one. As it stands, readers and future citers should treat the brain-wide MD findings as associated with, rather than demonstrated to be caused by, sensory deprivation per se, and the "experience-dependent" framing throughout should be understood as the authors' working interpretation rather than as excluded from a homeostatic alternative. I'd encourage the authors to state this limitation with the same explicitness in the main text's "Limitations and future perspectives" section as they have in their rebuttal.

Open Access This Peer Review File is licensed under a Creative Commons Attribution 4.0 International License, which permits use, sharing, adaptation, distribution and reproduction in any medium or format, as long as you give appropriate credit to the original author(s) and the source, provide a link to the Creative Commons license, and indicate if changes were made.
In cases where reviewers are anonymous, credit should be given to 'Anonymous Referee' and the source.
The images or other third party material in this Peer Review File are included in the article's Creative Commons license, unless indicated otherwise in a credit line to the material. If material is not included in the article's Creative Commons license and your intended use is not permitted by statutory regulation or exceeds the permitted use, you will need to obtain permission directly from the copyright holder.
To view a copy of this license, visit https://creativecommons.org/licenses/by/4.0/

Response to Reviewers

We thank the reviewers for their thoughtful and constructive evaluations of our manuscript and for their suggestions on revisions. In accordance with their advice, we revised the manuscript to address their concerns, improve clarity, and temper interpretations. Below we respond point-by-point to their comments, which are reproduced in italics.

We have inserted line numbers into the main manuscript and refer to these line numbers in our responses below.

Reviewer #1:

This manuscript investigates 'synaptome architecture' and the impact of experience in mice. The authors report that environmental enrichment (EE) and monocular visual deprivation (MD) produce widespread and subtype-selective reorganisation of excitatory synapses in the mouse brain, including >100 brain regions. They find that experience selectively alters subtypes of synapses, and associate this with protein turnover rates and connectome architecture. This experience-dependent 'synaptome plasticity' occurs during development and adulthood. They propose a population-selection model whereby such experiences specifically alter selective synapse varieties, and thus create a distributed trace of past experience.

These are novel findings. The manuscript is generally well written and the data clearly presented. I have suggestions below as to how the manuscript could be improved.

---

1. The title states that 'Experiences are encoded'. It is not clear that the authors have demonstrated that the changes they observe are encoding the experiences. More evidence would required to demonstrate a causative relationship. Or the title could be changed to reflect exactly what has been demonstrated in the present manuscript.

We have removed "encoded" and simplified the title to "Experience and sensory input reconfigure synaptome architecture of the developing and mature brain".

---

2. The last sentence of the Abstract states that 'Our results also point to synaptome architecture as a shared framework integrating experience, lifespan changes, sleep, genetic variation and disease'. Whilst the present manuscript is highly informative regarding the effects of experience on synaptome architecture, it does not show that this shared framework is involved in sleep, genetic variation, disease, etc. Some rewording of

this last sentence of the Abstract could draw the focus back to what has been demonstrated in the present manuscript.

At lines 38-40 we address how the synaptome reconfiguration in response to experience and sensory input shares common features with aging, gene mutations, brain injury and sleep deprivation. This indicates that reorganisation of synaptome architecture by endogenous and exogenous factors is a unifying principle. As recommended, we have reworded the last sentence of the abstract.

---

3. *In the Monocular Deprivation (MD) experiment, did the authors provide sham anaesthesia, analgesia to the control mice? If not, could some of the observed changes be due to extra handling, anaesthesia, analgesia, the stress of surgery, etc.?*

We did not perform sham anaesthesia in control mice. The anaesthesia and surgery were brief (<15 minutes) and performed 6 days prior to tissue collection, making it unlikely that acute effects account for the observed changes. Moreover, there was no difference in the handling of the MD and control animals (all animals were handled daily to check their eyes) and all were co-housed. We could find only one study in the last 60 years that used sham-operated controls and they reported similar brain-wide effects to our findings (

https://www.jneurosci.org/content/44/19/e1019232024).

We have brought this issue to the reader's attention in the revised Methods and in the new Discussion section "Limitations and future perspectives" [lines 522-533].

---

4. *Regarding the above point, could more analysis be done in the MD mouse brains comparing the two hemispheres for relevant visual areas, to establish what impacts are due to visual deprivation per se, and which might be due to differential handling, anaesthesia, algesia, surgical stress, etc.?*

As noted in point 3 above, there was no difference in handling of MD and control animals. In the original manuscript we compared the two hemispheres (see Fig. 2G and Fig. S14-16). We show region- and subtype-specific differences between the hemispheres. We have expanded the results [lines 236-251] accordingly.

---

5. Following up the above points on MD, it is stated that there were many changes induced by MD outside the visual areas, and not specific to contralateral hemisphere. This could be explained if the controls were not 'sham controls' and were not exposed to anaesthesia, analgesia and surgical stress/handling. This is a key issue the authors need

to address. If the controls were not exposed to anaesthesis, analgesia, etc., it may also be that they were more active in the home cage (relative to MD mice recovering post-surgery) and had altered circadian/sleep rhythms, etc.

We refer to our response to point 3 and 4, above. A recent MD imaging study that included a sham-surgery cohort also reported brain-wide effects, suggesting that distributed responses are not unique to the present design: https://www.jneurosci.org/content/44/19/e1019232024. We have added this study to the Discussion as relevant context, while emphasising that future experiments incorporating sham-operated controls will be needed to distinguish deprivation-specific from procedural effects more definitively [lines 522-533].

---

6. The authors describe Bayesian Analysis and need to provide more methodological details of exactly what this involved.

We thank the reviewer for pointing this out. While the original manuscript outlined the Bayesian framework used, we agree that the original Methods description of the Bayesian analyses was too compressed and did not provide sufficient detail for readers unfamiliar with this framework. We have therefore expanded the Methods to provide a clearer description of the model formulation, priors, MCMC settings, and the posterior inference procedure used to quantify evidence for group differences across regions and synapse parameters.

---

7. It is not exactly clear how the 37-subtype classification system was established. More detail would be welcome.

We thank the reviewer for this guidance and have now included a paragraph in the introduction that explains this [lines 70-83]. In addition, we have further expanded the description of subtype classification in the Methods.

---

8. The EE or SC conditions were for 3 months from a week before birth (including EE of both dams and embryos/pups until weaning). It is inaccurate to call it juvenile EE, as the juvenile period in mice is often considered somewhere in the range of 4-10 weeks, depending on the definition. However, most researchers routinely define mice as adult after two months of age.

We have now changed the term “juvenile EE” to “Developmental EE”, which we note is a description this reviewer has used in their point 11, below.

Regarding the comment that “most researchers routinely define mice as adult after two months of age”. In our lifespan study of the mouse synaptome architecture (Cizeron et al., *Science*, 2020) we found that the synaptome architecture continues to develop until the age of 3 months, which is why we chose this time point. To explain this, we have added text in the Methods and in the first paragraph of the results section [lines 99-100].

---

9. When EE was initiated approximately 7 days before birth (according to the protocol provided), did this disrupt dam-pup interactions? Could some of the effects be due to stress of imposing such an environmental change during the last week of pregnancy, and throughout weaning period? Could some effects be indirect, due to changes in maternal care, lactation, suckling, etc., rather than impacts on the offspring (from neonatal to young adult stages) themselves? Could these disruptive effects on the dams have disrupted brain development (including synaptogenesis) in the last week of pregnancy, and into the postnatal period?

These are all interesting questions and it is well beyond the scope of the current manuscript to disentangle all the variables that may contribute to the experiential changes of animals in different environments. We have raised these issues in the “Limitations and future perspectives” section of the discussion [lines 505-508].

Nevertheless, one of the striking findings is the high degree of similarity in synaptome architecture between “Developmental EE” and “Adult EE” (see Fig. 1C, 1G and 1H), consistent with the idea that much of the developmental synaptome response reflects the enriched environment itself.

---

10. How did the authors determine the time frame of EE (three months for young cohort and one month for the older cohort)?

As noted in point 8 above, we decided to use the first 3 months of postnatal life because our previous study (Cizeron et al, Science 2020) shows this is the period during which the synaptome architecture matures to adulthood. The same study showed that the synaptome architecture remains very stable between 3 to 12 months of age and it was during this age-window that we chose to expose the adult mice to 1 month of EE. This was noted in the results [lines 169-171] and methods sections to provide context for the chosen EE windows.

---

11. Do the authors have information on how much the EE mice (both developmental and adult EE) interacted with the EE objects (in the mazes, etc.), and how much physical activity they had (including on running wheels), relative to SC controls?

We did not record quantitative measures of interaction with individual enrichment features (e.g., maze use or object interaction), nor did we measure physical activity such as running-wheel use, in either the developmental or adult EE cohorts. We recognise the potential for future synaptome mapping studies that dissect the role of activity, sensory novelty, cognitive challenge, and social/environmental complexity, and have discussed this in "Limitations and future perspectives" section [lines 505-508].

---

12. EE protocols are intrinsically variable and difficult to reproduce. Even superficially similar EE protocols do not always have the same effects on brain and behaviour, so references to the effects of EE in prior publications may not reflect the protocol used in this study. It would be good to know if the authors, using the EE in this manuscript, had observed specific changes in behaviour (and/or cellular/molecular changes beyond the reported synaptome mapping), including cognitive performance, for example.

We agree with the reviewer that the variability in EE protocols is a general limitation in the field. It was for this reason that we chose to use the well-described commercial Marlau cages (https://pubmed.ncbi.nlm.nih.gov/23342033/), which have a standard design and can be reproduced by others. We highlighted our use of the Marlau cage in the Methods and Discussion under "Limitations and future perspectives" [lines 509-513]) and now include references to studies which also used the Marlau cage and recorded alternative measures of neuronal plasticity, as well as cognitive and behavioural effects.

---

13. The authors state that 'These findings suggest that EE does not broadly promote global synaptogenesis'. However, this was from one EE protocol (commercial Marlau cages), started at birth and continued to 3 months of age. For all of the reasons listed above (including the possibility that starting EE at birth stressed both the dams and pups and altered development) I don't think this one finding demonstrates that EE does not promote global synaptogenesis. I suggest such language be toned down a little.

We agree with this and have expanded the discussion of the results and their limitations [see lines 463-475].

14. Furthermore, do the authors have more molecular data (e.g. transcriptomics or proteomics) that might inform molecular mechanisms mediating the observed synaptome plasticity.

While our identification of a role for protein turnover provides some insight into molecular mechanisms, we agree that it would be informative to have transcriptomics and proteomics too. However, for these approaches to achieve the spatial resolution of brain regions and subregions of our synaptome approach, it would require spatial transcriptomics and proteomic laser capture dissection, which is beyond the scope of the present study. We note that our publicly available resource would enable anyone undertaking such studies to integrate their analyses with synaptome datasets.

---

15. The authors state that they excluded males due to fighting in male cages in the 'adult EE' experiments (consistent with some previous literature in this field). This leads to a limitation in being unable to address sex effects in this female-only adult EE experiment. Is there a way around this limitation (for example, not pooling adult males that are not littermates, to avoid the fighting that might be result of establishing new dominance heirarchies amongst stranger mice)?

We agree that the female-only design of the adult EE experiment limits our ability to assess sex-specific effects in this cohort, and we have now made this limitation clearer in the manuscript [lines 513-520]. We did consider whether adult males could be included under alternative grouping conditions, including transfer with familiar cage-mates or littermates to minimise aggression associated with the establishment of new dominance hierarchies. However, in our hands, adult male transfer to Marlau-cage EE remained problematic. The larger and more complex environment appeared to exacerbate aggressive interactions, and in a separate pilot attempt, this resulted in serious injury, requiring early termination of the male cohort on welfare grounds. We therefore judged that inclusion of adult males under these conditions would introduce substantial animal-welfare concerns and additional behavioural confounds, including injury, stress and altered social hierarchy effects, which would complicate interpretation of the synaptome data.

---

16. How did the authors control for any variability in image background between batches (which might affect detectability of synapses at threshold, etc.)? Were there batch effects?

To minimise variability in image background and detectability between imaging sessions, all scans were acquired using a standardised automated NIS-Elements JOBS workflow with fixed optical settings, including laser power, exposure time, aperture, emission filter, gain, bit depth, and autofocus procedure. High-magnification imaging was performed under matched acquisition settings across experiments, and imaging parameters were validated using saturation and signal-to-noise ratio analyses before large-scale acquisition.

In addition, puncta were detected using a classifier trained to distinguish signal from background on Nikon-acquired images spanning multiple ages, and downstream regional analyses showed consistent PSD95 and SAP102 density, size, and intensity patterning across animals despite the expected biological inter-animal variation. To make this clearer, we have expanded the Methods.

We agree that residual batch-related variation can never be excluded entirely in large-scale imaging experiments. However, we did not observe evidence of gross batch-specific distortion of the expected regional synaptome patterning, and the consistency of these region-wise patterns across animals supported inclusion of all samples in downstream analyses.

---

17. In the Discussion, there are broad statements made regarding the effects of EE, but it should be noted that the authors used a very specific type of EE, Marlau cages (sold commercially). They provide access to 3 running wheels per Marlau cage, and different configurations of a maze that was changed every two days. It is possible that mice spent most of their time on running wheels. And the novelty of the changes to this one maze may have worn out after a week or two. So the relative novelty and complexity in the EE cages (over time) in the present study, and the relative amounts of physical activity (versus sensory and cognitive stimulation) may vary widely from other published EE studies (where the developmental starting points and duration of EE also vary widely) with which the authors are make their comparisons. The 'devil may be in the detail' in such EE protocols, and these findings of synaptome changes (over space and time) may not generalise to other protocols.

We thank the reviewer for this important point, which is similar to point 11, above. Please see responses in point 11 and 13 above.

Reviewer #2 (Remarks to the Author):

This study examines whether experience alters brain-wide "synaptome architecture" by quantifying the distribution of excitatory synapses classified by PSD95 and SAP102 expression. Using environmental enrichment and monocular deprivation paradigms, the authors report widespread but subtype-specific changes across more than 100 mouse brain regions. Contrary to earlier literature, total synapse density is largely unchanged or modestly reduced. Instead, experience appears to redistribute predefined synapse subtypes. These effects are reported during development and adulthood. Subtype sensitivity correlates with PSD95 protein lifetime and, more weakly, with connectome projections. The authors propose a population-selection model in which experience modifies synapse subtype frequencies rather than synaptic strength. The work is technically ambitious and data-rich, but interpretation rests heavily on two scaffold proteins. Conceptual overreach and limited mechanistic depth weaken the broader claims. Overall, the conclusions substantially exceed what the data convincingly demonstrate.

Major comments

---

1. Overall conceptual concern: overinterpretation from two proteins
A central issue is that the paper repeatedly frames its findings as "single-synapse resolution synaptome mapping", which is misleading. The entire classification rests on PSD95 and SAP102 only, two postsynaptic scaffold proteins. While important, these represent a tiny fraction of synaptic molecular diversity. Claims about "synapse proteomes," "synapse varieties," or "synaptome architecture" therefore substantially overreach the data. This terminology should be reworded throughout to avoid implying molecular completeness.

Our method images individual synaptic puncta and therefore operates at single-synapse resolution. It is important to make this point because most studies that have examined the effects of EE or MD have not resolved individual synapses, but instead examine population averages. This issue is discussed in detail in Grant and Fransén (https://doi.org/10.3389/fnsyn.2020.590403).

We agree with the reviewer that two proteins reveal a limited fraction of excitatory synaptic molecular diversity, which we discussed in detail when the approach was first described (Zhu et al., *Neuron* 2018). We recognize that it would be helpful to bring this issue back to the current manuscript and have discussed it in the new section "Limitations and future perspectives" [lines 550-552].

The term “synaptome architecture” has been previously defined (Zhu et al, *Neuron* 2018; Cizeron et al, *Science* 2020) and is used here in that established sense to describe the spatial organisation of synapse populations classified by molecular features.

We agree that the term “varieties” is imprecise and have replaced it with the more accurate terms “types and subtypes” throughout.

Finally, we have carefully reviewed the use of the term “proteome” and revised the text accordingly.

---

2. Heavy inferences from descriptive redistribution
The study is almost entirely descriptive: synapse subtype densities are compared across conditions using effect sizes and clustering. Yet the Discussion repeatedly infers encoding of experience, memory traces, and adaptive selection mechanisms. These are speculative and not directly tested. No functional readout (electrophysiology, behavior linked to subtype shifts, causality) is provided. Statements suggesting that experience is “recorded” in synaptome architecture should be toned down or explicitly framed as hypotheses.

Directly linking molecularly defined synapse type and subtype changes to electrophysiological or behavioural readouts would require experiments capable of measuring synaptic physiology, morphology, and molecular subtype identity at the level of individual synapses, ideally before, during, and after EE or MD. Additionally, to identify which synapses in any of the highly distributed regions are contributing to the behavioral changes would require selective manipulation of specific synapses in any or all regions of the mouse brain together with a behavioural assay. Such multimodal and longitudinal experiments are not presently feasible and therefore beyond the scope of the present study.

However, there is an abundance of literature that describes synaptic physiological changes in response to EE and MD, and we discuss how the observed changes in types and subtypes may underpin those physiological changes and at the same time point out that those studies measure synapse population averages and not individual synapse physiological properties.

We have removed the term “recorded”, and we now refer instead to experience-associated reconfiguration of synapse type and subtype distributions and discuss the possible functional implications of these changes.

---

3. Discrepancy with prior EE literature. The authors report no increase in synapse density after environmental enrichment, in contrast to many previous studies. This discrepancy is acknowledged but not seriously addressed. A hypothesis is required. Possibilities include:

• *PSD95/SAP102-based detection missing other synapse populations*
• *Enrichment driving synaptic pruning with refinement, not net growth*
• *Age, duration, or enrichment protocol differences*
• *Over-reliance on puncta-based density rather than ultrastructural synapse counts*
Without such discussion, the result appears either underinterpreted or method-dependent.

As requested, we have edited the manuscript to provide several hypotheses including those suggested by the reviewer [see lines 463-475]

---

4. Methods and representation are very hard to follow
The paper is exceptionally difficult to read, even for specialists. Core concepts—synapse types, subtypes, protein lifetime classes, PCA clustering, effect size heatmaps—are introduced piecemeal and assumed knowledge from prior publications. This manuscript must be self-contained.
Specifically:
• *Synapse subtype classification should be explained from first principles in the main text, not as a follow-up shorthand.*
• *The reader should not need to know previous synaptome papers to understand what is being measured.*

We agree that the manuscript needed to be more self-contained and that several core concepts were not explained clearly enough for readers unfamiliar with the synaptome-mapping literature. In the revised manuscript, we have expanded the main text, Methods, and figure legends to explain from first principles the synapse type/subtype classification framework, the protein lifetime groupings, and the interpretation of the PCA, clustering, and effect-size heatmap analyses. We have also added bridging text throughout the Results to clarify how these analyses relate to one another and how the corresponding figures should be interpreted. In addition, to guide the reader through the overall approach more clearly, we have included a schematic overview of the synaptome mapping and analysis pipeline (Fig. S1). While the scale of the dataset necessarily requires data-dense visualisation and statistical summary, we have revised the text to make the analytical

framework substantially easier to follow without changing the core structure of the paper. We hope these revisions make the manuscript more self-contained and accessible.

5.Statistical representation: Cohen's d
Cohen's d is used extensively but never properly explained. For a biology audience, this is problematic. What does a given effect size correspond to in biological terms? Why is Cohen's d preferable to absolute or percentage change here? The authors should include a brief intuitive explanation and justify its use for cross-region comparisons.

We thank the reviewer for this helpful comment. We agree that the original manuscript did not explain clearly enough why Cohen's *d* was used or how it should be interpreted in this context. In the revised manuscript, we now describe Cohen's *d* as a standardized measure of the magnitude of a group difference relative to within-group variability, and clarify that its value here is to permit comparison across brain regions and synapse parameters with very different absolute scales. This standardized representation has also been used in previous synaptome mapping studies, allowing consistency across analyses, but we agree that the rationale needed to be made explicit for readers unfamiliar with that literature. We have therefore added explanatory text to the Methods, Results and figure legends.

---

6. Conceptual issue: comparing synapse subtypes across animals
It remains conceptually unclear how synapse subtypes are meaningfully compared across animals and conditions. Are subtypes assumed to be fixed molecular entities with stable identity? How robust is subtype assignment to variability in expression levels, imaging conditions, or normalization? This is a foundational assumption of the entire study and needs explicit justification.

We agree that the basis for comparing synapse subtypes across animals and conditions could be made clearer. In this study, synapse subtypes are not presented as fixed molecular entities in an absolute sense. Instead, they are defined as reproducible analytical classes within the established synaptome-mapping framework. These classes are based on shared measurements of molecular composition, fluorescence intensity, size, and shape, extracted and classified in the same way across all samples.

The reason subtype densities can be compared across animals and conditions is that all synaptic puncta were processed using the same imaging pipeline, feature extraction steps, and predefined classification framework. This allowed each punctum to be assigned to a common subtype space. Therefore, subtype identity is defined relative to a

stable analytical framework, rather than being newly defined within each animal or experimental group.

We also agree that subtype assignment could, in principle, be affected by variation in expression levels, imaging conditions, or other technical factors. We have therefore revised the Methods to explain the steps taken to minimise this variability, including standardised imaging settings, Nikon-specific classifier training and adaptation, and quality-control assessment of regional synaptome patterning across animals. We have also clarified in the Methods and main text that the 37 subtypes come from a previously established synaptome taxonomy, and that the aim of the present study is to compare how the abundance of these consistently defined classes changes across animals and conditions.

---

7. Protein lifetime analysis: interesting but opaque
The protein lifetime result is arguably the most interesting aspect of the paper. However:
• It is poorly explained how PSD95 lifetime was estimated and linked to current data.
• Figures 3A and 3B are extremely hard to interpret, and the legends are insufficient.
• The logic connecting lifetime, subtype stability, and experience-driven change needs clearer exposition.
This section should be substantially rewritten for clarity.

We agree that the protein-lifetime analysis was under-explained in the original manuscript and that the logic linking subtype-specific lifetime, synapse stability, and experience-associated remodelling was not presented clearly enough. In the revised manuscript, we have substantially clarified this section in three ways. First, we now explain more explicitly in the Methods that the PSD95 protein-lifetime estimates assigned to each subtype were derived from a previous synaptome protein-turnover study, in which subtype-specific average PSD95 lifetimes were measured and found to be reproducible across brain regions and animals. Second, we have rewritten the relevant Results text and expanded the figure legends to explain more clearly how subtype effect sizes were ordered by lifetime class and how the resulting heatmap patterns should be interpreted. Third, we have clarified the inferential step linking these prior lifetime measurements to the present data, so that the analysis is presented as a test of whether experience-associated subtype changes are associated with previously defined subtype-level proteostatic properties, rather than as a direct measurement of protein turnover in the current experiment.

---

8. Connectome alignment: correlation without causation
The integration with Allen connectivity data is technically impressive but conceptually

weak. Correlation between projection density and subtype changes does not establish mechanistic alignment. The analysis risks appearing as post hoc pattern matching. The authors should more explicitly acknowledge limitations and avoid causal language.

We thank the reviewer for this important comment. We agree that the connectome analysis is correlational and does not establish causation or mechanistic alignment. We have revised the manuscript to remove causal wording and to clarify that this analysis was intended to test for large-scale spatial association between baseline connectivity architecture and experience-associated synaptome remodelling. We have also added text in the Discussion that addresses future directions for linking connectome and synaptome data in studies where remodelling occurs [lines 535-542].

9.Trivial or overstated conclusions
Several claims are either obvious or overstated:
• *That experience activates distributed networks is not novel.*
• *That different experiences produce different molecular signatures is expected.*
• *The claim that subtype redistribution challenges classical synaptic plasticity models is incorrect; these frameworks are not mutually exclusive.*
These points weaken the Discussion by overstating novelty.
We have revised the first part of the Discussion to address these points.

We agree with the reviewer that the activation of distributed networks by experience is well established from electrophysiological, calcium imaging, and fMRI studies, and we ensured that examples of prior findings were cited. The statement was made to set the stage for the argument that followed in relation to why we see changes in the synaptome across many brain regions.

We respectfully disagree with the comment that “different experiences produce different molecular signatures is expected” and with the view that this is “trivial”. There has been no prior demonstration of brain-wide, single-synapse-resolution changes in synapse type and subtype composition across defined behavioural paradigms. We were very surprised by the results, especially for MD.

We agree that synaptome changes are not mutually exclusive with classical models of synaptic plasticity and removed all reference to this. We have explained how changes in molecular composition (as reflected in types and subtypes) may underpin changes in synaptic physiology [see lines 377-395].

---

10. Development vs genetics
The statement that synaptome architecture is primarily genome-encoded during development is misleading. Experience-dependent plasticity is central to developmental synapse organization. This section should be revised to better reflect the balance between genetic programs and experience.

The reviewer may have misinterpreted our Discussion, as we did not state that "synaptome architecture is primarily genome-encoded during development." Rather, we wrote: "Synaptome architecture is established during development by a hierarchical cascade of genome-encoded molecular mechanisms, including transcriptional, translational, and post-translational processes that control the synthesis and spatial distribution of protein complexes across diverse synapse types in the dendritic arbor".

We acknowledge that we had not adequately explained that these genome-encoded mechanisms are also the direct targets of activity-dependent and experience-dependent modulation and have revised the Discussion accordingly [see lines 441-458].

Minor comments and presentation issues
• *Figures are visually dense, difficult to decode, and insufficiently annotated.*
• *Heatmaps lack intuitive guidance for non-expert readers.*
• *The manuscript would benefit from summary schematics explaining the pipeline.*
• *Abbreviations and subtype numbers are overwhelming and poorly motivated.*
• *Language frequently inflates descriptive findings into conceptual claims.*

Regarding the figures. The main Figures do contain many panels and some of them are small, which is an unfortunate trend in many large-scale papers in recent years. We have now ensured that all such figures have been reproduced into the supplementary figures with all detailed annotations. The reader is directed to these corresponding figures via the figure legends. We have now included a summary schematic explaining the pipeline in Fig. S1.

In the revised manuscript, we have expanded the main text, Methods, and figure legends to explain more clearly the synapse classification framework, the protein-lifetime groupings, and the interpretation of the heatmaps, PCA, and clustering analyses. We have also sought to minimise the use of abbreviations and would be happy to replace these with full terms throughout, in line with the Editor's preference.

Finally, we have revised the language throughout to avoid inflating descriptive findings into conceptual claims. We hope these changes make the manuscript substantially easier to navigate while preserving the depth of the dataset.

Overall assessment and recommendation
This is a technically ambitious, data-heavy study that extends a previously established synaptome-mapping framework. However, the manuscript suffers from poor clarity, conceptual overreach, and limited mechanistic insight. Too many strong claims are based on redistribution of synapse classes defined by only two proteins. The most compelling result–the linke to protein lifetime–is underdeveloped and obscured by presentation.

We hope that the modified manuscript has satisfactorily addressed these issues

---

Reviewer #3 (Remarks to the Author):

The article by Woods et al. reports the effects of enriched environment and 7 days of monocular deprivation during the critical period on the synaptic proteome of PSD95 and SAP102, which was monitored through fluorescent-tagged proteins from knock-in mouse lines. They report that either manipulation caused brain-wide changes, which were similar for the EE groups when performed throughout development or just for a month in adult mice. It is interesting to see so widespread changes, especially for a supposedly restricted manipulation of the visual system. This supports previous reports that plasticity does not occur just locally but rather brain-wide. This result further addresses the need for proper controls when, e.g., assigning specific brain areas to specific behaviors. While the aim of the study is very laudable, several concerns remain.

We thank the reviewer for their supportive comments.

---

1) Several confounding factors could influence the brain-wide changes. This includes but is not limited to the switch to single housing after MD surgery (this was not specified in the methods). Removing mice one by one for analysis leads to arousal of the remaining ones, which could influence brain-wide activity.

Mice were not switched to single housing after MD surgery. The MD animals remained co-housed with their littermate controls throughout the experiment. Animals were also not removed for analysis in a fixed condition-specific order. Rather, tissue collection was performed in random order across groups, so any transient arousal effects on the remaining cage-mates would not be expected to bias one condition systematically over the other. We have now clarified these point in the revised Methods section.

---

2) For the MD control, a sham surgery should be performed. It was not clear from the methods whether this was the case.

We did not perform sham surgery in control mice. We could find only one study in the last 60 years that used sham-operated controls, and they reported similar brain-wide effects to our findings; https://www.jneurosci.org/content/44/19/e1019232024. We also note that many studies focus solely on the hemisphere contralateral to the deprived eye. The anaesthesia and surgery were brief (<15 minutes) and performed 6 days prior to tissue collection, making it unlikely that acute effects account for the observed changes. In the new "Limitations and future perspectives" section, we raise these issues and discuss them in relation to published literature [see lines 522-533].

---

3) The EE mice were analysed at an age of P81 to P96. These are mature mice. Thus, the claim of a conserved mechanism of synaptome plasticity across the lifespan appears overstated, as critical periods of the sensory areas end much earlier with likely different plasticity.

Although distinct in detail, we observed a high degree of similarity in the modification to the synaptome architecture between "Juvenile EE" (now renamed "Developmental EE" as suggested by Reviewer 1) and "Adult EE," indicating that conserved principles operate in the developing and adult brain.

We agree with the reviewer that our adult EE cohort does not address early critical periods directly. However, this was addressed by our MD experiments, performed during the critical period of visual development, which show that synaptome remodelling at this stage yields a qualitatively distinct signature to that obtained with EE, consistent with the reviewer's suggestion. We have modified the Discussion to clarify and further this point [see lines 372-375]

4) It is concerning that no clear effects of MD on contra- versus ipsilateral visual cortex were described. Differences have been widely reported, including on the proteome level. The results between the contra and ipsi visual cortex should be reported. The normalized data with comparisons between MD and control mice make it difficult to reveal such differences. Fig S9 and S10 are very similar, meaning after MD, contralateral and ipsilateral went through a similar reorganization process, but the brain reactions to external stimuli are very different in the contra vs ipsi hemisphere due to MD. Thus, it does not represent the physiological changes broadly observed.

We thank the reviewer for asking for further description of the contralateral and ipsilateral effects of monocular deprivation, particularly in visual cortical regions. We have therefore revised the Results to better guide the reader through the hemisphere-specific analyses already presented in the main and supplementary figures [see lines 236-251].

We respectfully disagree, however, that the similarity between the ipsilateral and contralateral effect-size heatmaps necessarily indicates a failure to capture physiologically relevant MD effects. These heatmaps show MD-control effect sizes within each hemisphere, whereas the normalised contralateral analysis and absolute inter-hemispheric difference analyses more directly assess hemispheric balance. Considered together, these analyses suggest that 6 days of MD produces a mixed pattern of shared bilateral synaptome reorganisation and region-specific shifts in hemispheric balance.

MD does not require all synaptome changes to diverge in opposite directions between hemispheres. Following unilateral deprivation, shared cortical adaptation, reduced inter-hemispheric asymmetry, and region-specific increases in asymmetry are all plausible outcomes, depending on synapse subtype, region, and time point. Consistent with this, several subtype-region combinations showed increased relative contralateral representation in MD mice, while many regions showed reduced absolute inter-hemispheric differences, suggesting partial convergence between hemispheres after altered visual input. Although these trends did not survive corrected significance testing, they indicate hemisphere-specific structure in the MD response across subtypes and regions.

We recognise that previous MD studies have reported contra–ipsi differences in visual cortical physiology and molecular composition. Our study addresses this question at a different scale and measurement level, using molecularly classified excitatory postsynaptic puncta across many brain regions after 6 days of MD. To our knowledge, direct proteomic comparisons of contralateral and ipsilateral hemispheres in MD mice at this synapse-subtype and brainwide anatomical scale have not previously been reported.

We hope that the expanded text around these points provides sufficient clarity on this interesting issue.

---

5) The results were interpreted as experience-dependent plasticity. However, as presented, the conclusion is weak, as the changes might have a homeostatic origin, which would be equally likely. Manipulation such as EE or dark rearing affects E-I balance, which could lead to differences in network activity. E.g., it could be tested whether these changes prevail after mice are returned to SC or after the eyes are opened. Experience-dependent changes should prevail, while homeostatic ones should relax.

We thank the reviewer for this interesting comment. The reviewer raises the possibility that homeostatic mechanisms might be responsible for the observed changes in synaptome architecture and suggests that they could be downstream of changes in excitability or inhibition. Secondary changes in the distribution of excitatory synapse types and subtypes might occur if EE/MD produced non-synaptic changes (e.g. changes in action potential thresholds, resting membrane potential). Arguably, this would still be a form of "experience-dependent plasticity", but not the classical form of Hebbian synaptic plasticity that is usually invoked in the EE/MD literature.

The reviewer also makes a good suggestion for future experiments which we now incorporate into the "Limitations and future perspectives" section [see lines 547-550].

---

6) Fig 4 uses the baseline connectome to align with the change in synaptome. But if some connections are low at baseline but enhanced after EE or MD, using only the control connectome will miss this correlation. Fig 4A shows the correlation coefficient; if the negative number means negative correlation, that is also a strong relation. Both of these aspects need to be addressed.

The reviewer raises the interesting point that experiential changes might alter long-range connections. Unfortunately, there are no experience-dependent connectome datasets available that allow this issue to be directly addressed, a limitation that we discuss in lines 535-542. Nevertheless, we consider the observed baseline correlations to be biologically informative because they suggest that pre-existing connectivity architecture may be related to regional sensitivity to synaptome remodelling.

As recommended, we have revised the Results text to further explain the positive and negative correlations [lines 326-330].

7) More than 30 subtypes of synapses were distinguished. Specific changes in the density of some subtypes were reported. This should then lead to complementary changes in other subtypes. One wonders whether those numbers add up or whether just some change by a significant amount, and the rest is diluted in the many other types.

We agree that, if some synapse subtype densities increase while others decrease, it is important to consider whether these changes balance within the overall synapse population. In the present study, this issue is addressed in part by the brain-wide maps of summed synapse subtype density shown in Figs. 1B, 1F, and 2B, which summarise the net effect across the full subtype population rather than only the individually significant subtype changes. These analyses indicate that the reported subtype-specific effects do not simply cancel completely across the total population. It is worth noting that although most excitatory synapses express either PSD95 or SAP102, there are some that do not (for example, there are some that express PSD93 but neither PSD95 or SAP102). So, we would not necessarily expect that the total number of PSD95 and SAP102 positive synapses would remain the same after an experience-dependent reconfiguration.

At the same time, we agree that the present data do not allow us to determine whether increases in some subtypes reflect direct transitions from other subtype classes, or whether additional changes may occur in synapse populations not captured by the PSD95/SAP102-defined framework. We have addressed this limitation in the “Limitation and future directions section” [see lines 544-547].

---

8) If these synaptome changes are characteristic of a behavioral manipulation, then one should be able to predict from the pattern of changes what experience the mouse had. Thus, one could test how accurately the synaptome of single mice would predict whether they belong to the control, MD, or EE group of the averaged dated of the other mice.

This is a great idea. In the “Limitations and future perspectives” section, we raised this as a future direction [see lines 552-557].

---

9) In the discussion, it is stated that the results “offer a new perspective on the classical model of synaptic plasticity”. The changes in synaptome could also represent different synaptic strengths, thus in accordance with classical synaptic plasticity models. With the data presented, I do not see how a firm conclusion could be drawn. Is the leading idea

here that synapse type shifts occur through shifts in proteostasis? How would processes like synaptogenesis or pruning factor in?

We have removed the paragraph about synaptic plasticity and rewritten the early part of the discussion to explain the relationship between synapse diversity and synaptic plasticity [see lines 377-407]

---

10) The distribution of SAP102 and PSD95 does not solely occur through genetic mechanisms during development, as stated. Dr. Constantin-Paton and others have shown the activity/experience-dependent shift of PSD95 in and out of synapses. Thus, even initially, interactions between genetic factors and activity for synaptic proteins have been described. This part requires more elaboration.

We agree that the distribution of SAP102 and PSD95 does not solely occur through genetic mechanisms during development. We wrote: “Synaptome architecture is established during development by a hierarchical cascade of genome-encoded molecular mechanisms, including transcriptional, translational, and post-translational processes that control the synthesis and spatial distribution of protein complexes across diverse synapse types in the dendritic arbor (Zhu et al., 2018)”. Our point was that the molecular mechanisms governing synaptic protein expression are genetically encoded. However, we did not explain that each one of these mechanisms is known to be modulated by experience (e.g. activity-dependent mRNA expression) and thus the hierarchical framework can be modulated to produce changes in synapse diversity. We have revised this section to better reflect the interaction between genetic programs and activity-/experience-dependent processes in shaping synaptome architecture, and now cite prior work from Constantine-Paton showing activity-dependent redistribution of PSD95 in and out of synapses [see lines 441-458].

---

11) Statements in lines 370-377 are not clear as it one hand highlights the effect on LPL subtypes showing their strongest associations with sensory pathways and then, in the next sentence, describe how, in the visual pathway, changes were driven in SPL and MPL subtypes. May need revision to be better understood.

We agree that this passage was not sufficiently clear. The first statement refers to correlations between baseline projection architecture and baseline (control) subtype densities, whereas the second refers to correlations between projection architecture and MD-induced changes in subtype abundance quantified by Cohen’s *d*. We have revised the text to distinguish these two analyses more explicitly and to clarify that the apparent contrast arises because one concerns the spatial organisation of subtype abundance

under baseline conditions, while the other concerns the spatial pattern of experience-associated remodelling [see lines 316-330]

RESPONSE TO REVIEWER COMMENTS

We thank the reviewer's for the support and constructive suggestions.

Below we have addressed the remaining comments, which are reproduced in italics.

Reviewer #1 (Remarks to the Author):

The authors have extensively revised the manuscript and adequately addressed almost all of my comments.

One comment, raised by myself and Reviewer 3, that for the MD experiment a sham-surgery control should have been used, has not yet been adequately addressed. The authors refer to the fact that in the 'MD field', sham surgeries are not often used. However, these studies (going back to Hubel and Weisel, and colleagues) usually only involved visual neurophysiology and used the unmanipulated eye/visual circuitry as an internal control. As the present MD experiment had a different design and different hypothesis (where the sham-surgery control becomes more crucial) the absence of this sham-surgery control remains a limitation.

We have acknowledged this limitation in lines 534-543: Our contralateral-versus-ipsilateral normalisation, adjusted using internal control data, was adapted from the inter-hemispheric normalisation approaches used in classical MD studies to derive ocular dominance indices [89,90]. This metric isolates deprivation-biased effects and revealed changes across multiple brain regions beyond the visual cortex. However, because it relies on an internal inter-hemispheric comparison, it cannot resolve bilateral changes shared equally across both hemispheres, which therefore remain potentially conflated with procedural effects. While such internal-control approaches are well established for classical, region-focused MD analyses, brain-wide interrogation of both unilateral and bilateral networks would benefit from explicit sham controls.

Reviewer #2 (Remarks to the Author):

The authors have done a great jiob at improving their manuscript which is now much clearer and provide important insight. I have no additional comments

We thank the reviewer for their supportive comments.

Reviewer #3 (Remarks to the Author):

I thank the authors for their detailed and largely constructive point-by-point response. The revision addresses most of my concerns satisfactorily, and I want to

acknowledge specific improvements before addressing what remains outstanding. The clarification on housing (point 1) is a straightforward factual correction that removes that confound from consideration. The expanded treatment of contralateral vs. ipsilateral effects (point 4) is a substantial improvement. The distinction between within-hemisphere effect-size heatmaps and inter-hemispheric balance analyses is now much clearer. I accept that partial convergence between hemispheres is a plausible outcome of unilateral deprivation rather than a failure of the method. I also appreciate the direct engagement with the subtype "conservation of mass" question (point 7) and the acknowledgment that PSD95/SAP102-negative synapses complicate any expectation of a fixed total. The response to point 10, incorporating literature on activity-dependent PSD95 redistribution, appropriately corrects the genetic/experiential framing.

We thank the reviewer for their supportive comments.

Three of my original points are addressed by acknowledgment rather than resolution. Sham-surgery controls (point 2). The authors' response, that the surgery was brief, performed six days before collection, and that one prior study using sham controls reported similarly broad effects, is a reasonable holding position, but it is not a resolution. It rests on a single external citation standing in for a control the present study itself lacks. Nonetheless, large-scale resource papers of this kind are valuable even before every confound is excluded. But I'd like to note that this leaves an open question about how much of the "brain-wide" MD signature reflects sensory deprivation specifically versus surgery, anaesthesia, or handling more generally. That distinction matters more here than in a typical single-region physiology study, because "brain-wide" is one of the paper's headline claims.

We have acknowledged this limitation in lines 534-543: Our contralateral-versus-ipsilateral normalisation, adjusted using internal control data, was adapted from the inter-hemispheric normalisation approaches used in classical MD studies to derive ocular dominance indices [89,90]. This metric isolates deprivation-biased effects and revealed changes across multiple brain regions beyond the visual cortex. However, because it relies on an internal inter-hemispheric comparison, it cannot resolve bilateral changes shared equally across both hemispheres, which therefore remain potentially conflated with procedural effects. While such internal-control approaches are well established for classical, region-focused MD analyses, brain-wide interrogation of both unilateral and bilateral networks would benefit from explicit sham controls.

Homeostatic vs. experience-dependent origin (point 5). Similarly, the authors agree this is a live alternative explanation and defer the discriminating experiment (testing whether the effects relax after return to standard housing or after eye-opening) to future work. That is an honest and appropriately scoped response, but it means the paper's central interpretation, that this is "experience-dependent plasticity" in the sense usually invoked in the EE/MD literature, remains an assumption rather than a demonstrated result.

We do not think this point needs to be addressed by revising the manuscript for several reasons. First, we respectfully disagree with the implication that homeostatic plasticity is not experience-dependent. While the original MD studies by Hubel & Wiesel and others were certainly interpreted in the context of Hebbian plasticity, many more recent papers frame experience-dependent changes in the visual cortex in the context of homeostatic regulation. Some references:

Murphy, K. M., et al. (2004). "Experience-dependent changes in NMDAR1 expression in the visual cortex of an animal model for amblyopia." Vis.Neurosci **21**(4): 653-670.

Mrsic-Flogel, T. D., et al. (2007). "Homeostatic Regulation of Eye-Specific Responses in Visual Cortex during Ocular Dominance Plasticity." Neuron **54**(6): 961-972.

Goel, A. and H. K. Lee (2007). "Persistence of Experience-Induced Homeostatic Synaptic Plasticity through Adulthood in Superficial Layers of Mouse Visual Cortex." Journal of Neuroscience **27**(25): 6692-6700.

Kaneko, M., et al. (2008). "Tumor Necrosis Factor-[alpha] Mediates One Component of Competitive, Experience-Dependent Plasticity in Developing Visual Cortex." Neuron **58**(5): 673-680.

Maffei, A. and G. G. Turrigiano (2008). "Multiple Modes of Network Homeostasis in Visual Cortical Layer 2/3." Journal of Neuroscience **28**(17): 4377-4384.

Kanold, P. O., et al. (2009). "Co-regulation of ocular dominance plasticity and NMDA receptor subunit expression in glutamic acid decarboxylase-65 knock-out mice." The Journal of Physiology **587**(12): 2857-2867.

Ranson, A., et al. (2012). "Homeostatic plasticity mechanisms are required for juvenile, but not adult, ocular dominance plasticity." Proceedings of the National Academy of Sciences **109**(4): 1311-1316.

Second, the term homeostatic plasticity is typically referred to as an electrophysiological property (see above papers as examples) and we are not measuring electrophysiology, we are measuring the molecular properties of synapses that are responsible for controlling electrophysiological changes.

Third, the changes we describe come about by complex biochemical mechanisms, which have numerous regulatory and feedback mechanisms, which include numerous homeostatic molecular mechanisms (e.g. kinase/phosphatase balances).

Predicting condition from single-mouse synaptome pattern (point 8). This is a missed opportunity, which could have been done with the existing data and strengthen the specificity of the observed changes.

We do not think this point needs to be addressed further.

With the revisions, the paper is now more accurately positioned as what it convincingly is: a technically ambitious, single-synapse-resolution atlas of brain-wide, subtype-selective synaptic reorganization following two well-chosen experiential manipulations. That is a substantial and citable contribution in its own right, and the revised framing does it justice rather than overselling it.

We thank the reviewer for their supportive comments.

However, the three open points above are precisely the ones that would be needed to move this paper from a descriptive resource to a more causally and mechanistically one. As it stands, readers and future citers should treat the brain-wide MD findings as associated with, rather than demonstrated to be caused by, sensory deprivation per se, and the "experience-dependent" framing throughout should be understood as the authors' working interpretation rather than as excluded from a homeostatic alternative. I'd encourage the authors to state this limitation with the same explicitness in the main text's "Limitations and future perspectives" section as they have in their rebuttal.

These limitations have been addressed in the preceding comments
